# Supplementary material for: Identification and functional evaluation of GRIA1 missense and truncation variants in individuals with ID: An emerging neurodevelopmental syndrome
Source: Am J Hum Genet. 2022 Jun 7;109(7):1217–41. doi: 10.1016/j.ajhg.2022.05.009 (PMC9300760; doi:10.1016/j.ajhg.2022.05.009)
Supplement: Document S1. Supplemental note, Figures S1–S7, Table S1, and supplemental methods [file mmc1.pdf]

The American Journal of Human Genetics, Volume *109*

Supplemental information

Identification and functional evaluation of *GRIA1* missense and truncation variants in individuals with ID: An emerging neurodevelopmental syndrome

Vardha Ismail, Linda G. Zachariassen, Annie Godwin, Mane Sahakian, Sian Ellard, Karen L. Stals, Emma Baple, Kate Tatton Brown, Nicola Foulds, Gabrielle Wheway, Matthew O. Parker, Signe M. Lyngby, Miriam G. Pedersen, Julie Desir, Allan Bayat, Maria Musgaard, Matthew Guille, Anders S. Kristensen, and Diana Baralle

Supporting Information

Supplemental Note: Case Reports

Individual 1 (p.Arg377Ter) is a 10-year-old female born to consanguineous parents. She is diagnosed with severe ID and delayed motor development. Although she walked at 14 months of life, her fine motor skills are delayed compared to peers, and she remains non-verbal. In addition, she displays self-injurious behavior and sleeping difficulties. She experienced her first epileptic seizure at six months of life and had recurrent seizures despite being treated with a wide range of anti-seizure medications. She is currently on lamotrigine and ethosuximide. An electroencephalogram showed diffuse background slowing with frequent bursts of spikes and spike-wave discharges across the posterior regions. We were unable to obtain further data in order to classify the seizure or epilepsy types. She also has precocious puberty, feeding difficulties requiring a gastrostomy, constipation, and an intermittent divergent squint.

Individual 2 (p.Ala636Thr) is a 6-year-old female. She is diagnosed with severe ID. She displayed an early developmental delay in gross motor skills. She sat unsupported at 1 year old and began walking at 2 years old. She is non-verbal at the present age. She is reported to have unspecified behavioral issues. No additional medical issues were reported aside from recurrent airway infections. She has not experienced epileptic seizures so far. The Individual was identified via the GeneMatcher platform[1] and is described in a previously reported study of 100 Individuals with severe ID (classed as an IQ below 50). As described in the original report, Individual and unaffected parents were subjected to diagnostic trio-WES analysis[2], which yielded the *GRIA1* missense variant, p.Ala636Thr, as a primary candidate (Table 1).

Individual 3 (p.Ala636Thr) is a 12-year-old male diagnosed with moderate ID. He displayed normal motor development. He sat unsupported at 6 months, walked at 18 months, and can currently run and ride a bike. He is non-verbal at the present age. He has autistic features and is hyperactive. He has poor sleep requiring melatonin and is mildly dysmorphic with telecanthus and full lips. He has not experienced epileptic seizures so far. The Individual was identified from the 100,000 Genomes Project (http://www.genomicsengland.co.uk) following WGS analysis that yielded the *GRIA1* missense variant, p.Ala636Thr, as the primary pathogenic variant candidate (Table 1).

Individual 4 (p.Ala636Thr) is a 26-year-old female with moderate ID. She has some gross motor delay and behavioral problems, including ADHD. Although she used her first word at 1 year of life and spoke in sentences at 2 years of life, she communicates using short and simple sentences and has dysarthria. Additionally, she had astigmatism requiring glasses at 11 months of life. This Individual was identified via the GeneMatcher platform[1]. A clinical exome of approximately 4000 genes was completed, which yielded the *GRIA1* missense variant p.Ala636Thr (Table 1).

Individual 5 (p.Ile627Thr) is a male of unknown age diagnosed with unclassified ID. Only limited access to clinical information was obtained for Individual 5, and further cognitive or motor impairment could not be reported. Other medical issues were reported to be migrainous headaches and a bicuspid aortic valve. Dysmorphic features described were full lips and a high arched palate. This Individual was identified from a study where targeted sequencing was completed for 20 recurrent sites of missense variants in 17,688 Individuals with NDD[3]. As described in the original report, single-molecule molecular inversion probes (smMIP) were designed to capture the regions of interest and the *GRIA1* p.Ile627Thr missense variant was identified (Table 1).

Individual 6 (p.Gly745Asp) is a 20-year-old female diagnosed with moderate-severe ID. She started walking at 13 months of life, but her fine motor skills are delayed compared to peers, and she was found to be hypermobile. She has a speech impairment, with regression from the age of 6 years old. She currently communicates using simple verbal language. She is diagnosed with ASD, bipolar disorder, and a sleeping disorder in addition to polycystic ovarian syndrome and hypothyroidism. The Individual was identified from a previously reported WES study of 213 Individuals with autism spectrum disorders[4] and via the GeneMatcher platform. As detailed in the original report, sequencing was performed on the Illumina HiSeq X Ten platform, and the *GRIA1* missense variant p.Gly745Asp was identified. This variant is also deposited in the ClinVar database (National Center for Biotechnology Information. ClinVar; [VCV000585059.2], https://www.ncbi.nlm.nih.gov/clinvar/variation/VCV000585059.2)[5].

Individual 7 (R345Q) is of unknown sex and age and was identified in the ClinVar database[5] (https://www.ncbi.nlm.nih.gov/clinvar/variation/VCV000983361.1) carrying the *GRIA1* missense variant

(p.Arg345Gln). Unfortunately, aside from being diagnosed with ID, there was limited clinical information available for this Individual at the time of publication.

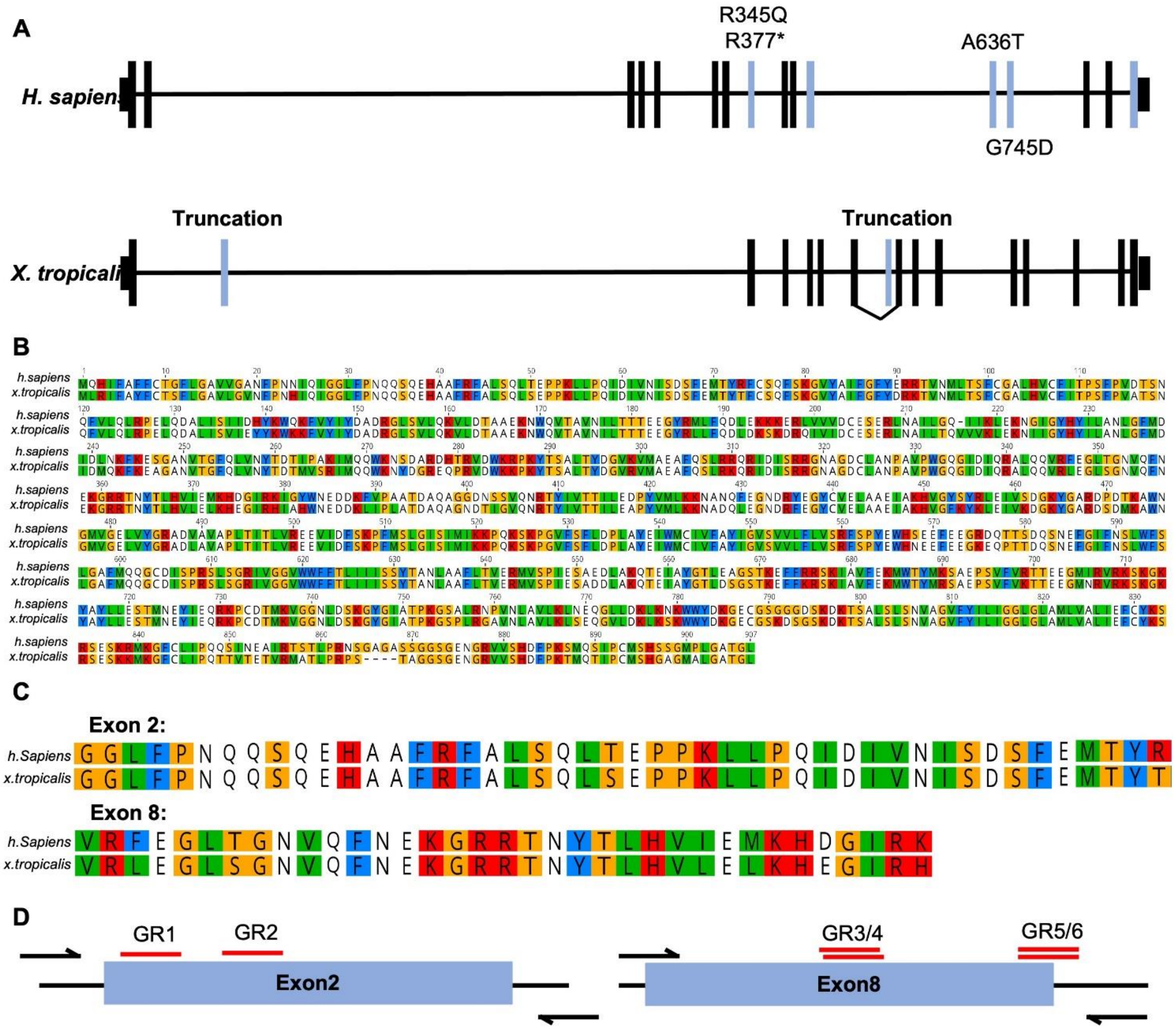

Figure S1. **Glutamate Ionotropic Receptor AMPA Type Subunit 1: gene structure, protein conservation and *X. tropicalis* target sites for disruption by CRISPR/Cas9.**

Xenopus tropicalis gria1, like *Homo sapiens gria1*, has 16 exons. The identified variants of unknown significance described causing GRIA1 Syndrome (bold) are shown spatially alongside the additional six variants listed on ClinVar. (A) The target exons for gene editing are highlighted in blue on the *X. tropicalis* model. (B-C) The *gria1* amino-acid sequence of *H. sapiens* and *X. tropicalis* aligned using Geneious Primer 2020 software (Biomatters, Ltd., Auckland, New Zealand) shows significant sequence conservation (>87%), particularly in the areas of interest in exons 2 and 8 (C). (D) The partial *X. tropicalis* exon schematics depict the position of the sgRNAs and genotyping primers for both the CRISPR screens.

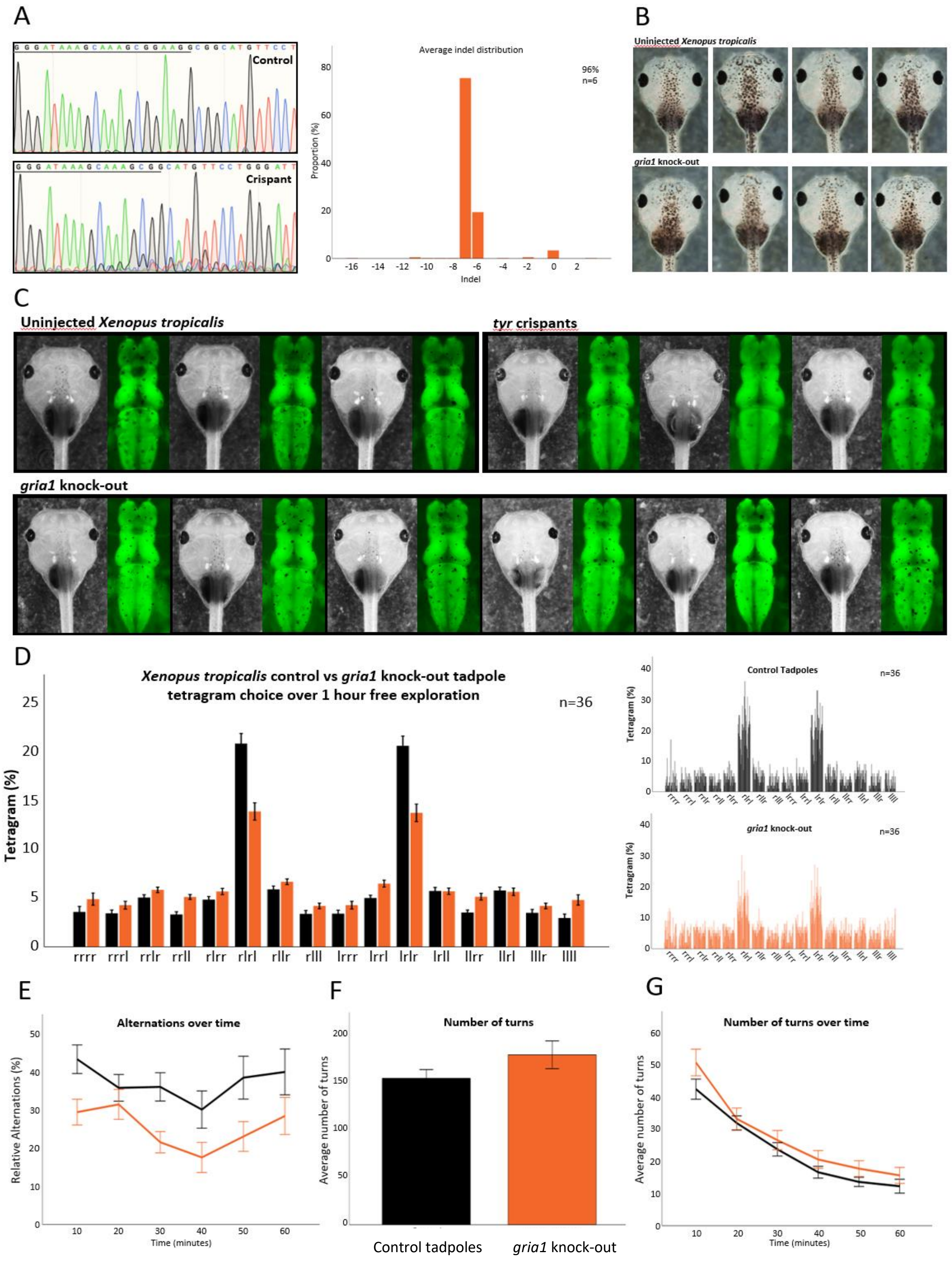

Figure S2. Characterization of tadpoles bearing CRISPR/Cas9 mediated insertion and deletion changes to *gria1* exon 2.

Fertilized *X. tropicalis* eggs were obtained in two mixed batches from three females and injected with sgRNA-GR2 targeting exon 2 of *gria1* and Cas9 protein. (A) Genomic amplicons were Sanger sequenced to confirm the presence of indels (*left*). The ratio of indels was assessed by Synthego ICE across six individual embryos, and the frequency is summarised as an average indel distribution in the mutant revealing a predominant 7 bp deletion. (B-C) Micrograph images of wild-type (*uninjected*), *tyrosinase* crispant (*tyr*), and *gria1* knock-out (*gria1*) tadpoles show head structural morphology under bright-field conditions (B) and whole brains in GFP fluorescence images. No morphological differences were observed in the forebrain, midbrain, and hindbrain regions of transgenic tadpoles [Xtr.Tg(tubb2b:GFP)Amaya, RRID: EXRC_3001]. *Gria1* knock-out tadpoles were generally indistinguishable from age-matched controls (*uninjected* and *tyr* crispant) both in their craniofacial appearance and brain length (forebrain to telencephalon: control Mean 2.24 mm, SD 0.3; knock-out Mean 2.14 mm, SD 0.29 [t_{70}=1.47; p=0.147], n = 36). (D) Relative frequency distribution plots of the free movement patterns of wild-type (*black bars*) and *gria1* knock-out (*orange bars*) tadpoles in the FMP Y-maze. Shown are summative (*left*) and individual (*right*) frequency distributions. For control animals, the alternating search patterns (LRLRL and RLRL) dominate, whereas for knock-out tadpoles, this dominance was significantly reduced. The data in the summative plot represents the mean ± SEM of all animals. (E) The number of alternations observed during the test period for wild-type (*black*) and *gria1* knock-out (*orange*) are shown in 10-minute time bins. *Gria1* knock-out tadpoles were observed to perform significantly fewer alternations than stage-matched control animals (ANCOVA: F(1, 69) = 18.9, p<0.001, n=36) throughout the trial. Data points represent mean ± SEM for all animals. (F & G) Overall, there was no significant difference in the number of turns performed by the control and *gria1* knock-out tadpoles (F: $t_{58.934}$ = -1.438, p=0.155), and all tadpoles were observed to perform fewer turns as the length of the trial increased (G: mean ± SEM).

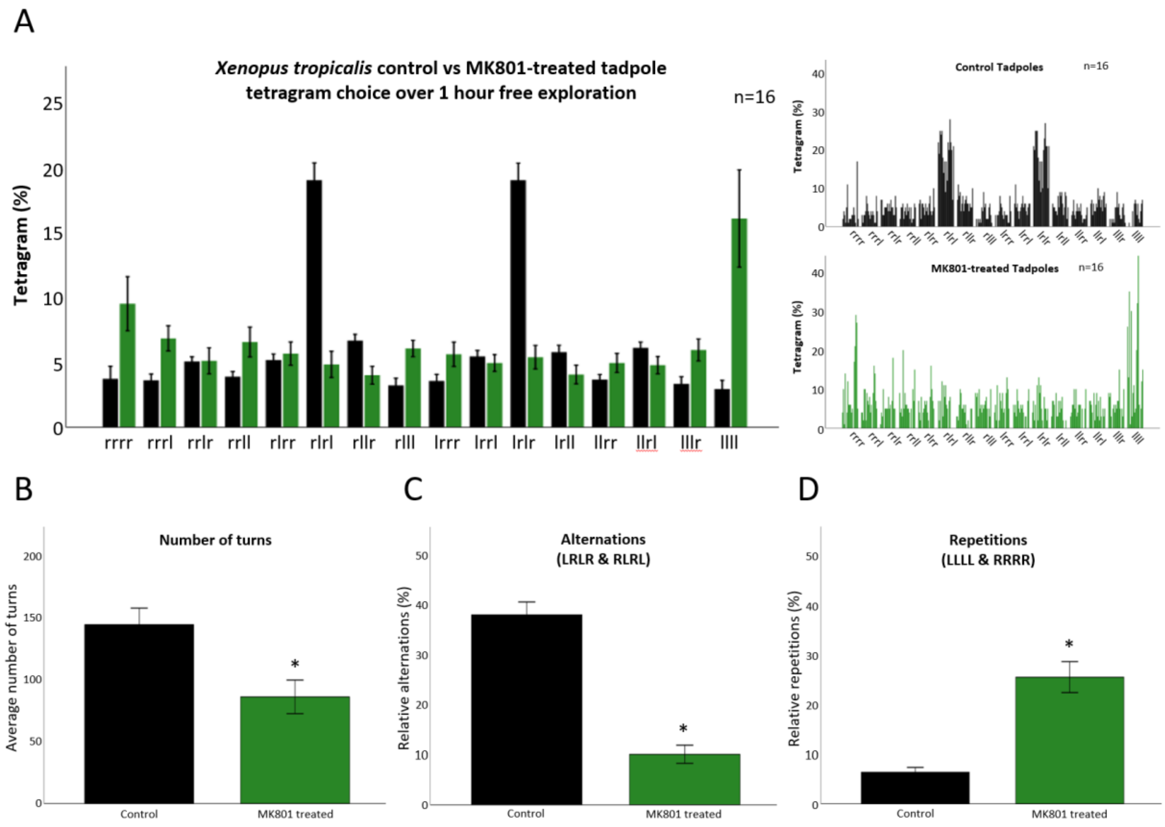

Figure S3. Implementation and pharmacological validation of the FMP Y-maze model for *Xenopus tropicalis* tadpoles.

Global tadpole search strategies in the FMP Y-maze were quantified as described in *Materials and methods*. The memory disrupting glutamate antagonist MK-801 was used to test whether search strategy patterns measure working memory in *Xenopus*. Wild-type tadpoles at stage NF50 were either treated with 0.75mg/L MK-801 (*green bars*) or remained untreated (*black bars*) in the two hours preceding behavioral analysis in the FMP Y-maze trial. (A) Relative frequency distribution plots of the free movement patterns of wild-type (*black bars*) and *gria1* knock-out (*green bars*) tadpoles in the FMP Y-maze. Shown are summative (*left*) and individual (*right*) frequency distributions. For control animals, the alternating search patterns (LRLRL and RLRL) dominate, whereas for MK-801 treated tadpoles, repetitive patterns (RRRR and LLLL) are dominant. The data in the summative plot represent the mean ± SEM of all animals. (B) The total number of turns during the trial for wild-type (*black bars*) and MK-801 treated (*green bars*) tadpoles. MK-801 treated tadpoles showed a significant decrease in the total number of turns performed across the trial ($t_{33.991}$ = 3.063, p=0.004). The data represent the mean ± SEM of all animals. (C - D) Direct comparison of the frequency of alternating search (LRLR and RLRL) and repetition (LLLL and RRRR) patterns between control and MK-801 treated tadpoles. Control tadpoles show a dominant search strategy of alternations (C: ANCOVA: F(1, 33) = 28.8, p<0.001; n=18), while MK-801 treated tadpoles display a dominant search strategy based on repetition (D: ANCOVA: F(1, 33) = 27.7, p<0.001; n=18). All data represent the mean ± SEM.

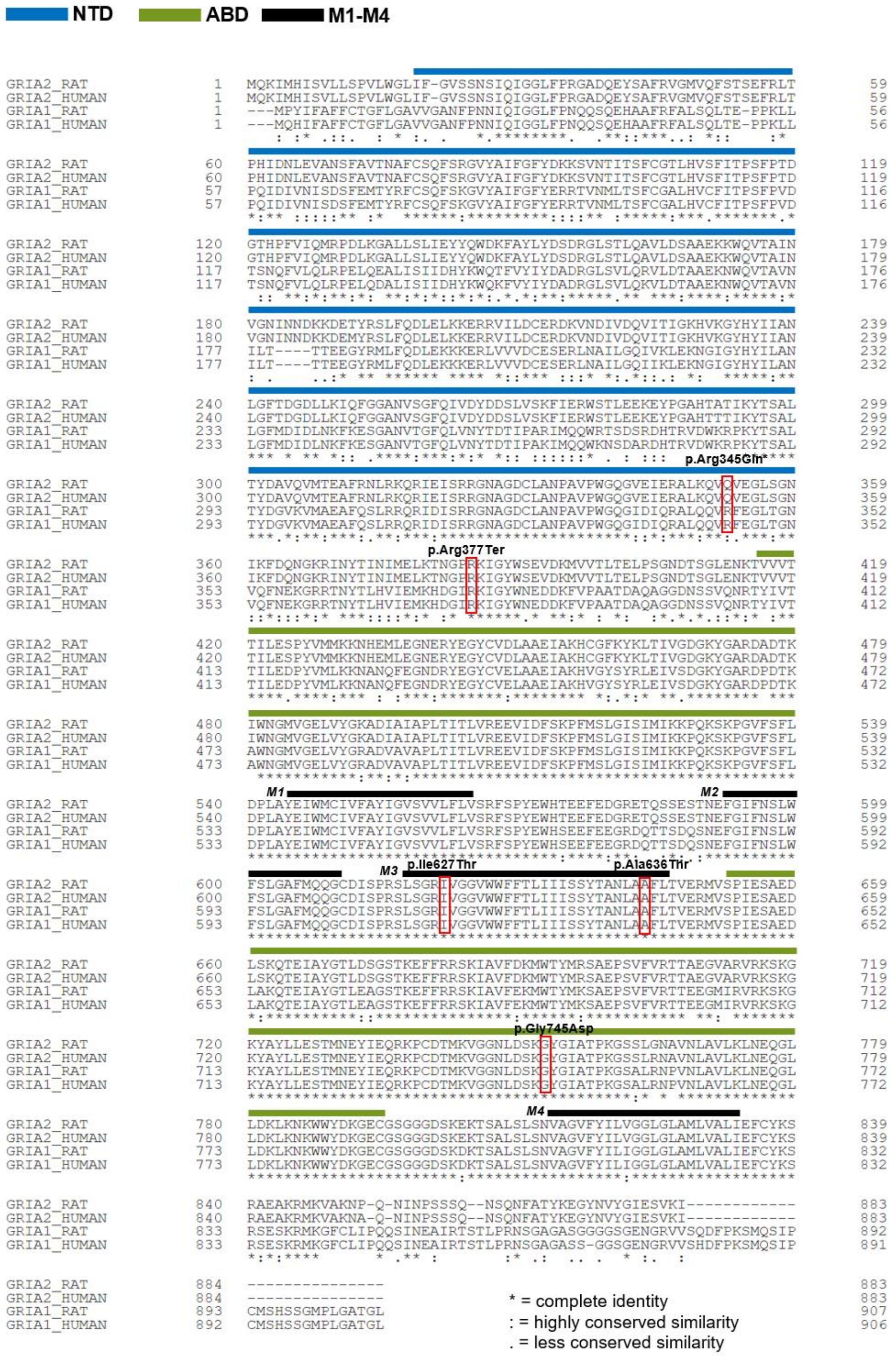

Figure S4. **Amino acid sequence alignment of human and rat GluA1 and GluA2 used for homology modeling of the human GluA1 receptor**.

The location of the NTD segment (*blue*), the ABD segments (*green*), and the transmembrane regions M1 to M4 (*black*) are depicted on top of the alignment. The residues affected by *GRIA1* variants are highlighted with red boxes.

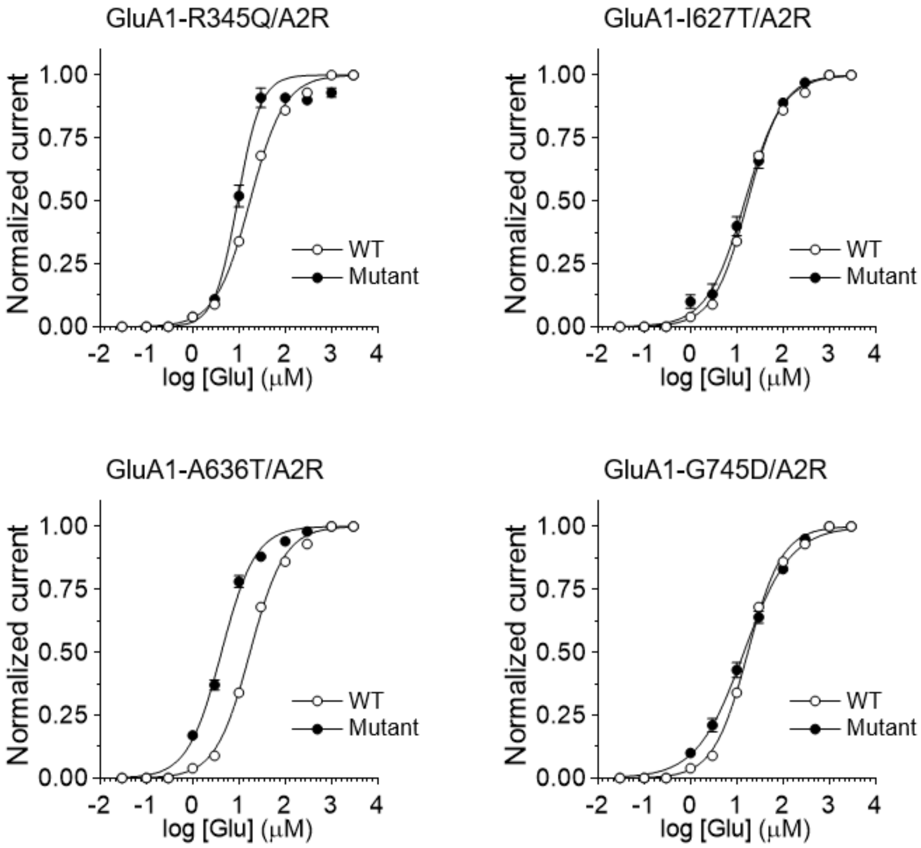

Figure S5. Glutamate concentration-response relationship for GluA1 mutants expressed as heteromeric GluA1/A2.

Composite concentration-response curves for glutamate at WT (○) and mutant (●) GluA1/A2 receptors were constructed using TEVC (*Materials and Methods*). Data represent the mean + SEM from 10-31 independent experiments.

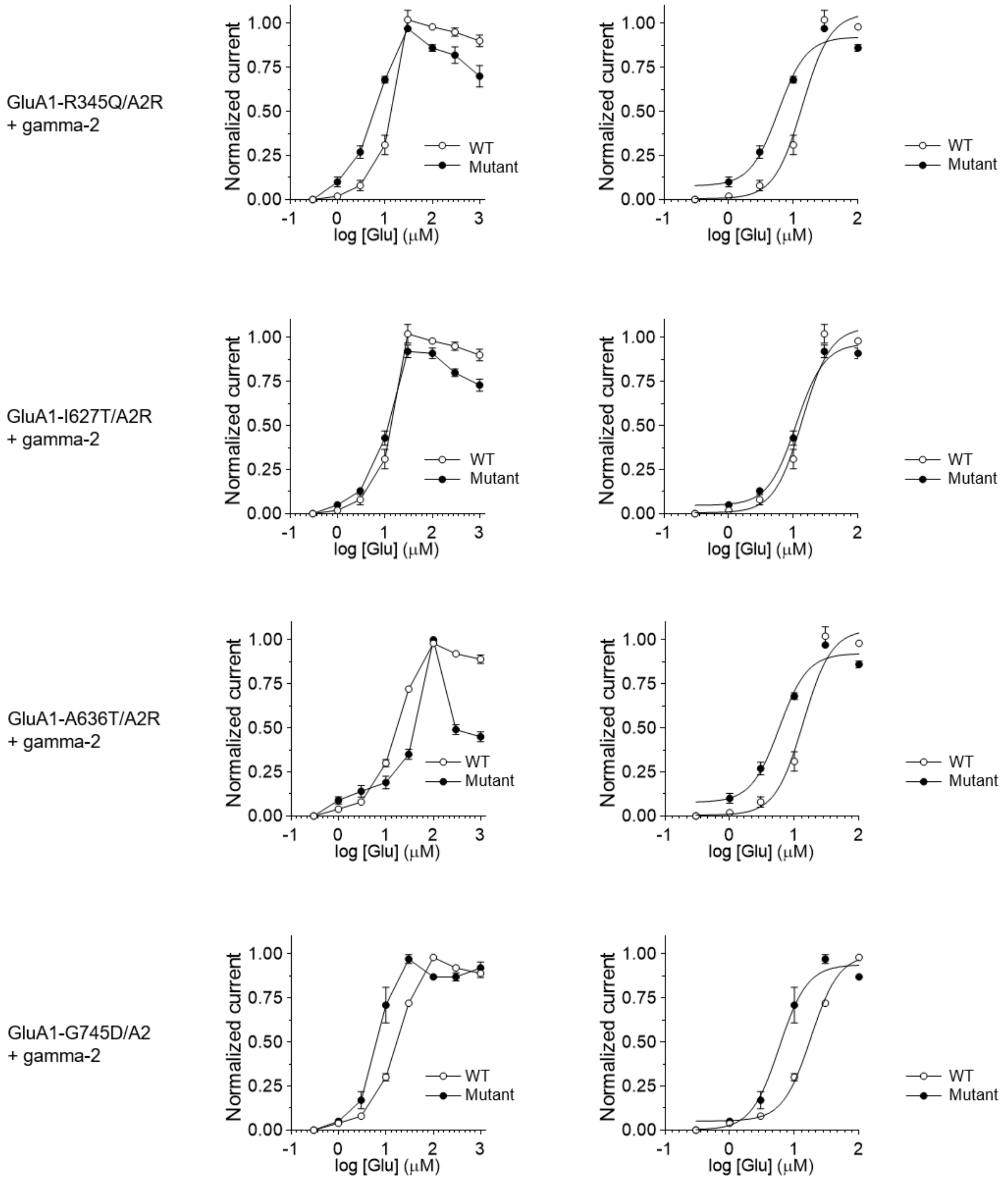

Figure S6. Glutamate concentration-response relationship for GluA1 mutants expressed as heteromeric GluA1/A2 in the presence of the TARP-class auxiliary subunit gamma-2.

Composite concentration-response curves for glutamate at WT (○) and mutant (●) GluA1/A2 receptors expressed with gamma-2 were constructed using TEVC (*Materials and Methods*). Data represent the mean + SEM from 10-31 independent experiments.

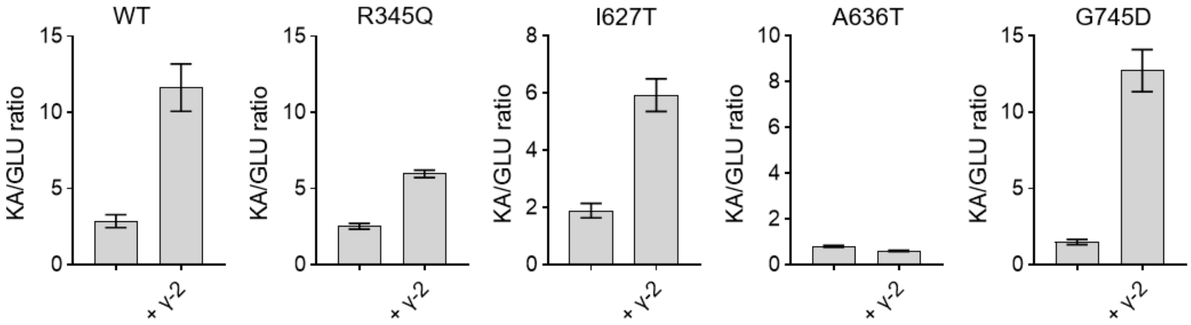

Figure S7. Control for the formation of gamma-2 complex for heteromeric GluA1/A2R receptors. The KA/Glu response ratio is increased upon co-expression with gamma-2 for all mutants and WT except for the A636T mutant. Data represent the mean + SEM from 10-31 independent experiments.

Gene	Target region	Primer sequence (gDNA)	#	CRISPR scan Score	Target sequence	Blast (v.10 *X. tropicalis*)	InDelphi Frameshift score
gria1	Exon 2: Gene knock-out	ACGACAGGAAAATAGCTGca AAGTGACCCCATTGCCTACA 435bp	GR1	75	GGTCCTGGGATTGCTGGTTT	16bp Chr8: pgk1 (non-coding), 16bp Chr4: non-coding	80.9% 64% +1, 17% +2
			GR2	61	GGATAAAGCAAAGCGGAAGG	17bp Chr1: non-coding, 16bp Chr1: LOC101730958 (non-coding), 16bp Chr7: non-coding	86.7% 17% +1, 70% +2
	Exon 8: p.Arg377* (homozygous)	ACTTCAAAGGTTCGGCTGGA CCTTTACCAGCTTAGTTGCCC 502bp	GR3	23	GGCGACCTTTCTCATTAAAC	17bp Chr2: non-coding, 17bp Chr1: ctxn1 (non-coding), 16bp Chr1: non-coding, 16bp Chr9: micall2 (non-coding), 16bp Chr8: non-coding, 16bp Chr5: non-coding, 16bp Chr3: non-coding	63.5% 34% +1, 29% +2
			GR4	33	GGTTTAATGAGAAAGGTCGT	17bp Chr3: non-coding, 17bp Chr1: ctxn1 (non-coding), 16bp Chr1: non-coding, 16bp Chr8: non-coding, 16bp Chr5: non-coding	61.1% 31% +1, 30% +2
tyr	Exon 2: Gene knock-out	CCTGCCGCTGACATATGGA GATGCCTCAGAGAGACGGAT 514bp	TY1	56	GGGGGTTCTGCTCCGATCG		84.7% 39% +1, 45% +2
			TY2	71	GGCTGTTGTAGGCAATCGGG	16bp Chr1: *rad9b* (non-coding)	74% 39% +1, 35% +2

Table S1. **Design considerations for selecting efficient CRISPR/Cas9 oligonucleotides for gene-editing *in vivo*.**

This table presents the *gria1* sgRNA CRISPR design considerations, providing an overview of each target (gene, target region, variant of unknown significance and genomic region of interest) and its associated oligonucleotide specificities (reference, CRISPRscan score, sequence, off-target blast hits, Indelphi frameshift frequency). Oligonucleotides were designed using CRISPRscan, which blasts the target genome (v9.1 *X. tropicalis*) against the reference sequence (c.50bp) and suggests constructs with on-target, efficient mutagenic potential (indicated by a high CRISPRscan score). All oligonucleotides were then manually blasted against either v10 (*X. tropicalis*) or v9.1 (*X. laevis*) of the *Xenopus* genome (Xenbase, e value 10), hits in grey represent genome locations lacking a suitable PAM (NGG) sequence, while hits in black show any potential off-target locations. Potential off-target hits differing more than 2bp from the original oligonucleotide sequence were also considered not viable. Fifty bp on either side of the expected CRISPR/Cas9 cut site was used to predict the likelihood of a deleterious frameshift variant (Indelphi). The frameshift score represents the overall proportion of variants that will generate an out-of-frame change, and this is then further subdivided into the proportion predicted to cause a +1 or +2 frameshift. The selected oligonucleotides above were synthesised by Invitrogen with a 5’ T7 promoter (taatacgactcactata) and 3’ overlapping Universal CRISPR segment (gttttagagctagaa).

SUPPLEMENTARY METHODS

Protein modeling – We modeled the flip isoform of the human homomeric GluA1 (hGluA1) receptor in the closed *apo* conformation (*resting*), the glutamate-bound, open-channel conformation (*active*), and the glutamate-bound, closed-channel desensitized conformation (*desensitized*). As templates for human GluA1 in these conformations, the following structures of the homomeric GluA2 receptor were used: Homomeric GluA2 in complex with the competitive antagonist ZK (PDB ID: 3RGK)[6] for the resting conformation, homomeric GluA2 with the auxiliary subunit gamma-2 in complex with glutamate and cyclothiazide (PDB ID: 5WEO)[7] for the open conformation, and the structure of homomeric GluA2 with the auxiliary subunit GSG1L in complex with the agonist *L*-quisqualate (PDB ID: 5VHZ)[8] for the desensitized conformation. Alignment of the rat GluA2 template structures to the hGluA1 sequence was aided by sequence alignment of the human and rat iGluA1 and GluA2 sequences (Figure S4). Homology modeling was performed using modeling software MODELLER (Sali and Blundell, 1993). For each confirmation of hGluA1, a total of 100 models were created and the best models were chosen based on Discrete Optimized Protein Energy (DOPE) scores calculated in MODELLER.

SUPPLEMENTARY REFERENCES

1. Sobreira, N., Schiettecatte, F., Valle, D., and Hamosh, A. (2015). GeneMatcher: a matching tool for connecting investigators with an interest in the same gene. Hum Mutat 36, 928-930.
2. de Ligt, J., Willemsen, M.H., van Bon, B.W., Kleefstra, T., Yntema, H.G., Kroes, T., Vulto-van Silfhout, A.T., Koolen, D.A., de Vries, P., Gilissen, C., et al. (2012). Diagnostic exome sequencing in persons with severe intellectual disability. The New England journal of medicine 367, 1921-1929.
3. Geisheker, M.R., Heymann, G., Wang, T., Coe, B.P., Turner, T.N., Stessman, H.A.F., Hoekzema, K., Kvarnung, M., Shaw, M., Friend, K., et al. (2017). Hotspots of missense mutation identify neurodevelopmental disorder genes and functional domains. Nat Neurosci 20, 1043-1051.
4. Guo, H., Duyzend, M.H., Coe, B.P., Baker, C., Hoekzema, K., Gerdts, J., Turner, T.N., Zody, M.C., Beighley, J.S., Murali, S.C., et al. (2019). Genome sequencing identifies multiple deleterious variants in autism Individuals with more severe phenotypes. Genetics in medicine : official journal of the American College of Medical Genetics 21, 1611-1620.
5. Landrum, M.J., Lee, J.M., Benson, M., Brown, G.R., Chao, C., Chitipiralla, S., Gu, B., Hart, J., Hoffman, D., Jang, W., et al. (2018). ClinVar: improving access to variant interpretations and supporting evidence. Nucleic Acids Res 46, D1062-D1067.
6. Sobolevsky, A.I., Rosconi, M.P., and Gouaux, E. (2009). X-ray structure, symmetry and mechanism of an AMPA-subtype glutamate receptor. Nature 462, 745-756.
7. Twomey, E.C., Yelshanskaya, M.V., Grassucci, R.A., Frank, J., and Sobolevsky, A.I. (2017). Channel opening and gating mechanism in AMPA-subtype glutamate receptors. Nature 549, 60-65.
8. Twomey, E.C., Yelshanskaya, M.V., Grassucci, R.A., Frank, J., and Sobolevsky, A.I. (2017). Structural Bases of Desensitization in AMPA Receptor-Auxiliary Subunit Complexes. Neuron 94, 569-580 e565.
