# Supplementary material for: Identification and functional evaluation of GRIA1 missense and truncation variants in individuals with ID: An emerging neurodevelopmental syndrome
Source: Am J Hum Genet. 2022 Jun 7;109(7):1217–41. doi: 10.1016/j.ajhg.2022.05.009 (PMC9300760; doi:10.1016/j.ajhg.2022.05.009)
Supplement: Document S2. Article plus supplemental information [file mmc2.pdf]

Identification and functional evaluation of *GRIA1* missense and truncation variants in individuals with ID: An emerging neurodevelopmental syndrome

Vardha Ismail,[1,13,14] Linda G. Zachariassen,[2,14] Annie Godwin,[4] Mane Sahakian,[2] Sian Ellard,[5,6] Karen L. Stals,[5] Emma Baple,[5,6] Kate Tatton Brown,[7] Nicola Foulds,[1] Gabrielle Wheway,[3] Matthew O. Parker,[8] Signe M. Lyngby,[2] Miriam G. Pedersen,[2] Julie Desir,[9] Allan Bayat,[10,11] Maria Musgaard,[12] Matthew Guille,[4] Anders S. Kristensen,[2,13,*] and Diana Baralle[1,3,13,*]

Summary

GRIA1 encodes the GluA1 subunit of α-amino-3-hydroxy-5-methyl-4-isoxazole propionate (AMPA) receptors, which are ligand-gated ion channels that act as excitatory receptors for the neurotransmitter *L*-glutamate (Glu). AMPA receptors (AMPARs) are homo- or heteromeric protein complexes with four subunits, each encoded by different genes, *GRIA1* to *GRIA4*. Although GluA1-containing AMPARs have a crucial role in brain function, the human phenotype associated with deleterious *GRIA1* sequence variants has not been established. Subjects with *de novo* missense and nonsense *GRIA1* variants were identified through international collaboration. Detailed phenotypic and genetic assessments of the subjects were carried out and the pathogenicity of the variants was evaluated *in vitro* to characterize changes in AMPAR function and expression. In addition, two *Xenopus gria1* CRISPR-Cas9 F_0 models were established to characterize the *in vivo* consequences. Seven unrelated individuals with rare *GRIA1* variants were identified. One individual carried a homozygous nonsense variant (p.Arg377Ter), and six had heterozygous missense variations (p.Arg345Gln, p.Ala636Thr, p.Ile627Thr, and p.Gly745Asp), of which the p.Ala636Thr variant was recurrent in three individuals. The cohort revealed subjects to have a recurrent neurodevelopmental disorder mostly affecting cognition and speech. Functional evaluation of major GluA1-containing AMPAR subtypes carrying the *GRIA1* variant mutations showed that three of the four missense variants profoundly perturb receptor function. The homozygous stop-gain variant completely destroys the expression of GluA1-containing AMPARs. The *Xenopus gria1* models show transient motor deficits, an intermittent seizure phenotype, and a significant impairment to working memory in mutants. These data support a developmental disorder caused by both heterozygous and homozygous variants in *GRIA1* affecting AMPAR function.

Introduction

α-amino-3-hydroxy-5-methyl-4-isoxazole propionate receptors (AMPARs) belong to the ionotropic glutamate receptor (iGluR) superfamily of ligand-gated cation channels that mediate the majority of excitatory synaptic transmission in the central nervous system.[1] The primary function of AMPARs is to facilitate synaptic transmission by delivering excitatory postsynaptic currents (EPSCs), but AMPARs are also involved in synaptic plasticity mechanisms thought to underlie learning and memory.[2–4] AMPARs form as tetrameric assemblies of the four subunits, GluA1–4, encoded by the *GRIA1–4* genes.[5] The *GRIA1* gene encodes the 907 amino acid GluA1 subunit (Figures 1A–1C). GluA1 can assemble as a homomeric receptor or combine with GluA2–4 subunits into heteromeric AMPARs. Structurally, AMPARs have a four-layer structure with the amino terminal domains (NTDs) from each subunit forming an upper extracellular layer, the agonist-binding domains (ABDs) forming a middle layer containing four Glu-binding sites, and the transmembrane domains (TMDs) forming a central membrane permeating ion channel (Figure 1B).

Neurodevelopmental disorders (NDDs) encompass a range of phenotypes, such as intellectual, behavioral, memory, or motor deficits, and are estimated to affect 1%–3% of the population in Western countries.[7–10] A growing body of evidence suggests that a substantial proportion of NDDs have monogenetic causes affecting key proteins in excitatory neurotransmission,[11–13] including *GRIA* genes. Advances in understanding the genetic

[1]Wessex Clinical Genetics Service, Princess Anne Hospital, University Hospital Southampton NHS Foundation Trust, Coxford Rd, Southampton SO165YA, UK; [2]Department of Drug Design and Pharmacology, University of Copenhagen, Universitetsparken 2, 2100 Copenhagen, Denmark; [3]Faculty of Medicine, University of Southampton, Duthie Building, Southampton General Hospital, Tremona Road, Southampton SO16 6YD, UK; [4]European *Xenopus* Resource Centre, School of Biological Sciences, King Henry Building, King Henry I Street, Portsmouth PO1 2DY, UK; [5]Exeter Genomics Laboratory, Royal Devon & Exeter NHS Foundation Trust, Barrack Road, Exeter EX2 5DW, UK; [6]University of Exeter Medical School, Royal Devon & Exeter NHS Foundation Trust, Barrack Road, Exeter EX2 5DW, UK; [7]South-West Thames Clinical Genetics Service, St George's University of London, Cranmer Terrace, London SW17 0RE, UK; [8]School of Pharmacy and Biomedical Sciences, University of Portsmouth, Old St Michael's Building, White Swan Road, Portsmouth PO1 2DT, UK; [9]Département de Génétique Clinique - Institut de Pathologie et de Génétique, Institut de Pathologie et de Génétique, Avenue Georges Lemaître, 25 6041 Gosselies, Belgium; [10]Danish Epilepsy Centre, Department of Epilepsy Genetics and Personalized Medicine, 4293 Dianalund, Denmark; [11]Department of Regional Health Research, University of Southern Denmark, 5230 Odense, Denmark; [12]Department of Chemistry and Biomolecular Sciences, University of Ottawa, 75 Laurier Ave E, Ottawa, ON K1N 6N5, Canada
[13]Senior author
[14]These authors contributed equally
*Correspondence: ask@sund.ku.dk (A.S.K.), d.baralle@soton.ac.uk (D.B.)
https://doi.org/10.1016/j.ajhg.2022.05.009.
© 2022 The Author(s). This is an open access article under the CC BY license (http://creativecommons.org/licenses/by/4.0/).

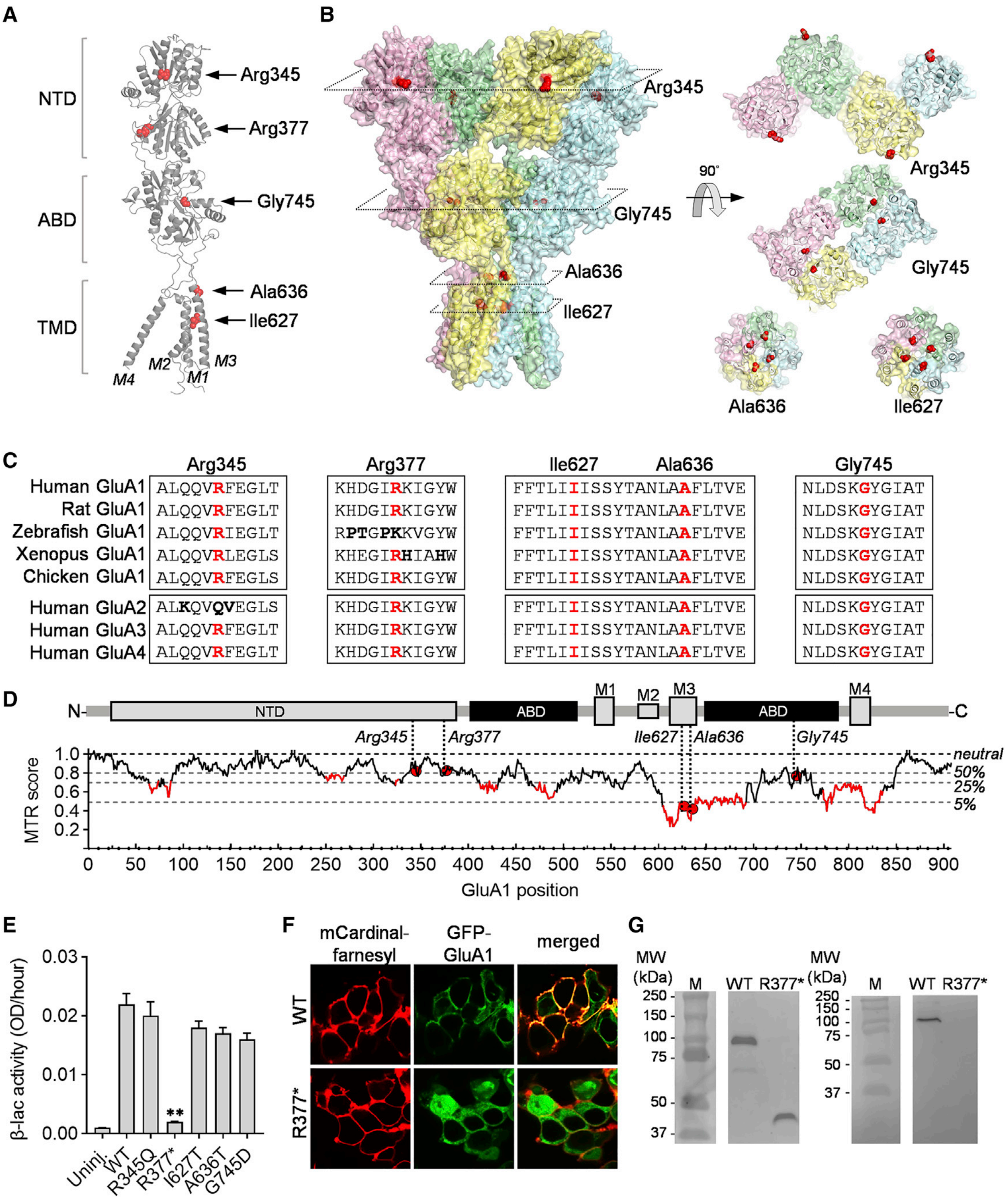

Figure 1. Location of residues in GluA1 affected by *GRIA1* variants and effect on receptor expression

(A) Cartoon representation of the structure and domain organization of the human GluA1 subunit protein (GenBank: NP_000817.1) encoded by the *GRIA1* gene. Residues affected by the GRIA1 variants evaluated in this study are shown in spheres (carbon and nitrogen atoms in purple and blue, respectively) and indicated with arrows. The GluA1 subunit structure was modeled from the structure of the rat GluA2 receptor (supplemental material and methods).

(B) Structure of the homotetrameric GluA1 AMPA receptor. The locations of the residues affected by the *GRIA1* missense variants are indicated by red spheres in the tetrameric receptor structure (left) and further shown in cross-sectional top views (right).

(C) Multiple alignment of the amino acid sequences that surround the residues affected by the *GRIA1* variants (highlighted in red) in GluA1 from human, rat, chicken, frog (*Xenopus*), and fish and in human GluA2, GluA3, and GluA4 subunits. Residues with diverging physiochemical properties are shown in bold.

(D) Missense tolerance ratio (MTR)[6] analysis of the population-level variation in the coding regions in *GRIA1* predict the tolerance of missense variation along the GluA1 primary structure to indicate residues or regions that are functionally sensitive to variation. The plot was created with the MTR-Viewer online service. Horizontal lines show gene-specific MTR percentiles 5th, 25th, 50th, and neutrality (MTR = 1.0). Red domains in the plot indicate regions in the primary structure of GluA1 that are highly sensitive to missense variation. The positions of the residues affected by the *GRIA1* variants in this study are shown as red circles. For reference, a linear representation of the GluA1 domain structure is shown above the plot.

(legend continued on next page)

architecture of the brain may begin to unravel the genetic causes for NDDs. In particular, appreciating the critical role of AMPARs in excitatory neurotransmission and synaptic plasticity mechanisms is yielding a new perspective for their causal role in NDDs. Previous studies have implicated *GRIA2*, *GRIA3*, and *GRIA4* genes in NDDs, but *GRIA1* has not yet been established as a disease-causing gene. Although variants in *GRIA1* have been identified through whole-exome sequencing (WES) and whole-genome sequencing (WGS) studies in cohorts of individuals with NDD[14–20] and a potential mutation "hotspot" in *GRIA1* has been postulated,[16] to date, there has been no detailed phenotypic analysis or functional work completed to classify these variants beyond uncertain clinical significance.

A cohort of unrelated individuals diagnosed with NDD with both new and previously reported *GRIA1* missense variants was identified through collaboration. In particular, this included an individual with a homozygous *GRIA1* stop-gain mutation that truncates the GluA1 subunit and appears to disrupt expression of any GluA1-containing AMPAR subtype. Functional evaluation of the impact of the *GRIA1* variants on the function of GluA1-containing AMPAR subtypes showed three out of four to have profound gain- or loss-of-function effects on important functional features of homomeric and heteromeric GluA1 receptor subtypes. In addition, the *in vivo* effects of disruption of GluA1-containing AMPAR expression was assessed in genetically altered *Xenopus* tadpoles with a novel behavioral model for measuring working memory. The results provide evidence that *GRIA1* variants are the cause of a monogenic NDD characterized by intellectual disability (ID), speech and language delay, poor sleep, abnormal electroencephalogram (EEG) with or without seizures, normal brain imaging, and endocrine abnormalities, adding to the existing collection of *GRIA*-related NDDs.

Material and methods

Cohort analysis

Subjects were identified through the authors' clinical practice or GeneMatcher[21] or ClinVar databases.[22] Medical information including birth parameters, epilepsy, EEGs, developmental histories, brain MRIs, and physical examinations were collected from the local healthcare providers. The study was conducted in agreement with the Declaration of Helsinki and approved by the local ethics committees. Because all individuals had cognitive impairment, their parents or legal guardians gave informed consent.

Genetic identification and analysis

Subjects 1 to 5 were investigated by WES or WGS ordered by primary healthcare providers or as part of larger research studies (supplemental note). Individual 6 was investigated by targeted panels (supplemental note). Information on genetic analysis was not available for individual 7. All variants were annotated with the GenBank: NM_000827.3 (GRCh37/hg19) transcript of *GRIA1*. The functional consequences of missense variants were predicted via calculation of combined annotation-dependent depletion (CADD) scores,[23] sorting intolerant from tolerant (SIFT),[24] and Polymorphism Phenotyping v2 (PolyPhen2)[25] analysis. We employed the Genome Aggregation Database (gnomAD v.2.1.1; https://gnomad.broadinstitute.org/) to determine the frequency of the variants in control populations.

Materials

All chemicals were from Sigma-Aldrich (St. Louis, MO) unless otherwise stated. Dulbecco's modified Eagle's medium (DMEM), fetal bovine serum, trypsin, and penicillin-streptomycin were from Invitrogen (Carlsbad, CA). DNA-modifying enzymes were from New England Biolabs (Ipswich, MA) except PfuUltra II Fusion HS DNA polymerase (Agilent, Carlsbad, CA). Tissue cell culture plasticware was from Sarstedt (Nümbrecht, Germany) unless otherwise stated. Cyclothiazide (CTZ), kainic acid, and NASP were from HelloBio (Bristol, UK). A DNA construct encoding GFP-GluA1 was a gift from Roberto Malinow (University of California, San Diego), and the construct encoding mCardinal-Farnesyl-5 was a gift from Michael Davidson (Addgene plasmid # 56159; http://n2t.net/addgene:56159; RRID: Addgene_56159).

Xenopus tropicalis husbandry

Adult Nigerian strain *Xenopus tropicalis* were housed and maintained within the European *Xenopus* Resource Centre, University of Portsmouth, in recirculating systems at 24°C–25°C with 15% daily water changes on a 13–11 h light-dark cycle. All work was conducted in accordance with the Home Office Code of Practice under PPL 79/8983 and PP4353452 following approval from the University of Portsmouth's Animal Welfare and Ethical Review Body. For egg recovery, female *Xenopus tropicalis* were primed with 10 IU of Human Chorionic Gonadotropin (Chorulon, Intervet) and boosted with 100 IU the following morning. Egg clutches were fertilized with cryopreserved sperm (EXRC).[26–28] Embryos were cultured at 27°C for the first 24 h and 24°C thereafter in 0.05 X Marc's Modified Ringer's (MMR in mM: 22 NaCl, 0.5 KCl, 0.5 $CaCl_2$, 0.25 $MgCl_2$, 1.25 HEPES, pH 7.4), in complete darkness with a 50% media change every other day, with twice-daily health

(E) Biochemical assessment of WT and mutant GluA1 cell surface expression. Summary of blac enzyme activity levels from the surface of oocytes expressing WT and mutant GluA1 receptors tagged in the N terminus of the NTD for measurement of receptor cell surface level. The enzyme blac catalyzes hydrolysis of the β-lactam ring of the membrane-impermeable substrate nitrocefin to cause a color change from yellow to red, which is measured as increase in optical density (OD) at 486 nm. As nitrocefin does not permeate the cell membrane, only extracellular blac activity is measured. The data shown are the mean ± SEM of parallel measurements from 10 to 16 live oocytes expressing the indicated receptor subunits at 48 h after RNA injection.
(F) Confocal imaging of HEK293 cells expressing the membrane reporter mCardinal-farnesyl (left images) and GFP-tagged WT and p.Arg377Ter (indicated by R377*) variant GluA1 (middle images) to visualize cellular GluA1 distribution patterns. Co-localization of green and red fluorescence indicates cell surface localization of GFP-tagged GluA1 and is visualized by merging left and middle images (right images), thereby appearing yellow.
(G) Immunoblot analysis of WT and p.Arg377Ter GluA1 protein expression in HEK293 cells with antibodies to the N and C termini of the protein (material and methods).

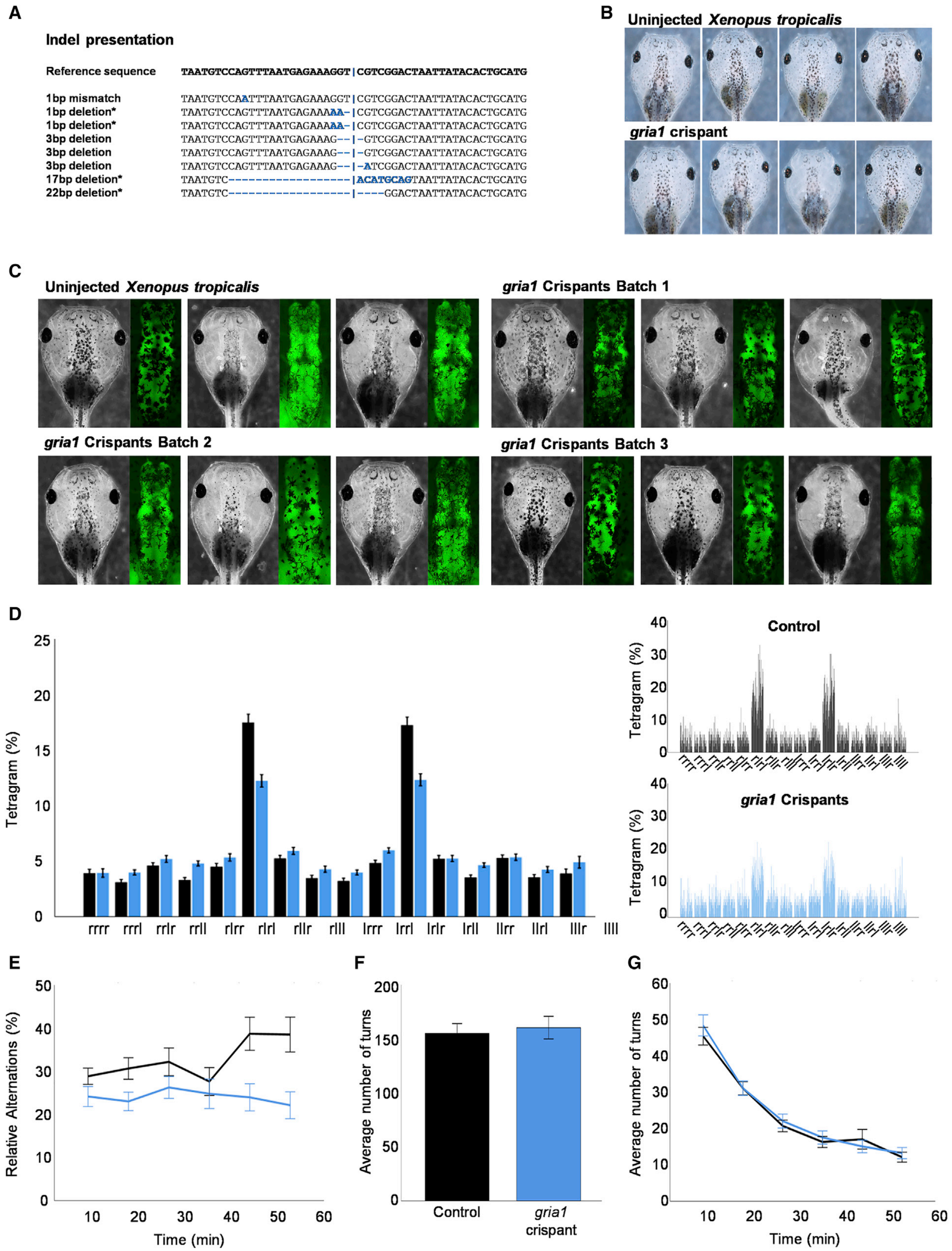

Figure 2. Tadpoles bearing CRISPR-Cas9-mediated insertion and deletion changes to exon 8, *gria1* (*Xtr.gria1*^{em2EXRC}) have significant deficits in working memory

(A) Targeted disruption within *gria1* exon 8 generates a range of insertion-deletion changes *in vivo*. Sanger sequencing of eight subcloned genomic amplicons (exon 8) from *Xtr.gria1*^{em2EXRC} tadpoles revealed a range of indels that occurred in samples at the CRISPR cut site

(legend continued on next page)

checks. Once at feeding stages, tadpoles were fed a mixed diet of spirulina and sera micron twice daily 5 days per week and once daily 2 days per week.

Animal strains and genetic alteration

Experimental data presented in this study were obtained from either wild-type (WT) Nigerian strain *Xenopus tropicalis* or a transgenic line expressing GFP in differentiated neural tissue [Xtr.Tg(tubb2b:GFP)Amaya] RRID: EXRC_3001 (from here on referred to as tubb2bGFP). In these background strains, three different CRISPR-Cas9-based mosaic models were made and analyzed as detailed below: a *tyrosinase* crispant in which exon 2 was targeted (*Xtr.tyr*^{em1EXRC} referred to as "*tyr* crispant" in the text and used as a control); a *gria1* crispant in which exon 2 was targeted (*Xtr.gria1*^{em1EXRC} referred to as "*gria1* knockout" in the text and described in Figure S2) and a second *gria1* crispant model in which exon 8 was targeted (*Xtr.gria1*^{em2EXRC} referred to as "*gria 1* crispant" in the text and described in Figure 2.).

Generation of knockout animals with CRISPR-Cas9

The target regions within *gria1* (exon 2 and exon 8) were identified with Xenbase,[29] and the single-guide RNAs (sgRNAs) were designed with v9.1 of the *X. tropicalis* genome and later blasted against v10 to check for additional off-target sites. Two single-stranded oligonucleotide templates (Table S1) for sgRNA synthesis were selected for each region on the basis of the following criteria: high mutagenic activity, minimal predicted off-target events, and a high frameshift frequency with the CRISPRscan[30] and inDelphi algorithms.[31] Following the Taq-based method described by Nakayama et al.,[32] single-stranded oligonucleotides (Invitrogen, UK) containing the T7 promoter were annealed and extended with the universal CRISPR oligonucleotide; this template was then transcribed with the T7 Megashortscript kit (Invitrogen, UK). The resulting sgRNAs were purified with SigmaSpin Sequencing Reaction Clean-Up columns (Sigma-Aldrich), quantified with a NanoDrop 1,000 spectrophotometer (Thermo Fisher Scientific, Loughborough, UK), analyzed by agarose gel electrophoresis, and stored at −80°C as single-use aliquots. Across all experiments, 1,000 pg sgRNAs were co-injected with 2.6 ng Cas9 protein (Spy cas9 NLS, New England Biolabs) into single-cell *X. tropicalis* embryos. The efficiency of indel formation was assessed in genomic DNA from crispant embryos. Briefly, lysates were prepared from three batches of embryos collected at Nieuwkoop and Faber (NF) stage 10 and stage 41 by incubation at 56°C for 2 h in 50 mM Tris, 1 mM EDTA, 0.5% [v/v] Tween 20, 100 μg/mL Proteinase K (pH 8.5). Primers for PCR amplification of the target regions of interest were designed with Primer3 software[33] (Table S1) and blasted against v10 of the *Xenopus tropicalis* genome (Xenbase). Amplicons were column purified (SmartPure PCR Purification Kit, Eurogentec, Belgium) and Sanger sequenced (Genewiz, UK), and the resulting trace files were compared with ICE v2 CRISPR Analysis Tool software (Synthego, Redwood City, CA). Indels were confirmed by Sanger sequencing of subcloned PCR amplicons from mutant animals.

Phenotypic analysis of crispant tadpoles

To identify gross morphological differences between uninjected tadpoles and crispant tadpoles, animals were anesthetized in 0.025% w/v tricaine mesylate solution and visually inspected with an AxioZoom V16 stereomicroscope (Zeiss, Jena, Germany) with fluorescence for visualizing GFP-expressing animals. To inhibit melanogenesis for the study of neural transgene expression, tadpole medium was supplemented with 75 μM 1-phenyl 2-thiourea (PTU) after hatching at NF26. Analysis of working memory was examined at stage NF50 with the Zantiks MWP unit (Zantiks Ltd, Cambridge, UK) and the free-movement pattern (FMP) Y-maze.[34] The FMP Y-maze is validated for assessing spatial working memory and cognitive flexibility in zebrafish and has been applied to both mice and humans but has never been used in *Xenopus*. The FMP Y-maze quantifies deviations from randomness in search strategies by analyzing a continuous log of arm entries in the maze in terms of discrete choices (i.e., "left" or "right"), grouped into a series of four overlapping choices, called "tetragrams" (e.g., LRLR, LLLR, etc.). Vertebrates such as zebrafish, mice, and humans show a common dominant search strategy (∼25%–30% of turns) of sequentially alternating left/right choices (LRLR or RLRL).[34,35] This pattern is abolished with memory-blocking drugs and reduces in aging.[34] The task is also able to quantify behavioral flexibility by analyzing the change across time in alternation strategies.[34] For example, behavioral flexibility is reduced with dopamine D_1 receptor antagonists and in the presence of acute stress.[34–36]

Xenopus tropicalis tadpoles were placed in semi-translucent, acrylic inserts containing two identical Y-mazes (three 10 × 10 × 25 mm arms and a 10 × 10 × 10 mm central zone). Inserts were filled with 0.05 X MMR and contained a dilute but equally distributed tadpole food mix. The Y-maze arms were of

(indicated by the blue line), including the re-occurring 1 bp deletion and 3 bp deletion. Further, four of the identified clones were found to cause a frameshift that truncated the protein (denoted by the asterisk).
(B) Representative micrograph images of the head region of uninjected control and *gria1* crispant tadpoles under bright-field conditions reveal no gross difference in craniofacial morphology.
(C) Representative bright-field (left) and fluorescence micrograph images (right, in green) of the head regions of transgenic [Xtr.Tg(tubb2b:GFP)Amaya, RRID: EXRC_3001] tadpoles reveal gross morphology of the forebrain, midbrain, and hindbrain regions. Crispant (*Xtr.gria1*^{em2EXRC}) tadpoles examined across three batches were generally indistinguishable from age-matched control tadpoles both in their craniofacial appearance and brain morphology. Specifically, brain length measured as the distance from the forebrain to the hindbrain (mean ± SD) was not significantly different between control and *gria1* crispant tadpoles (control tadpoles: 1.33 ± 0.1 mm, n = 36; crispant tadpoles: 1.31 ± 0.1 mm, n = 36, p = 0.537).
(D and E) The relative frequency distribution plots of the 1 h global search strategy of WT (black bars) and *gria1* crispant (blue bars) tadpoles in the FMP Y-maze. Shown is the summative (D, left, mean ± SEM) and individual (D, right) tadpole performances from 60 WT and 60 *gria1* crispant tadpoles. The *gria1* crispant tadpoles were observed to perform significantly fewer alternations than stage-matched control animals (ANCOVA: $F(2, 176) = 10.3$, $p < 0.001$ ($n = 60$), D), and this difference in the overall proportion of alternations performed was observed throughout the trial (E).
(F and G) Overall, there was no significant difference in the number of turns performed by the uninjected control and *gria1* crispant tadpoles ($t_{116.364} = -0.564$, $p = 0.574$), and all tadpoles were observed to perform fewer turns as the length of the trial increased (mean ± SEM).

equal size with no intra-maze cues, and lights were maintained off during each trial to match the rearing conditions of the tadpoles. An infra-red video camera was used for live monitoring and recording of the movement of individual animals. Sample size estimations were calculated *a priori* from pilot studies performed on 16 uninjected control tadpoles and 16 *gria1* knockout tadpoles with the G*Power software package version 3.1.9.7.[37] Tadpoles were transferred into the Y-mazes and placed into the Zantiks MWP unit for a pretrial time of 120 s to acclimatize. They were then tracked for 1 hour of free search. Data from each trial were output in two forms: zone entries/exits over time and an AVI video file (with live tracking). Zone entries and exits were converted into left or right turns, grouped into overlapping sequences of four turns (tetragrams) via customized Excel spreadsheets, and normalized against the total number of moves. For experiments with MK-801 ((+)-5-methyl-10,11-dihydroxy-5H-dibenzo(a,d)cyclohepten-5,10-imine]), the concentration and delivery was based on previously published work in zebrafish.[34] Specifically, tadpoles were placed in 100 mL beakers containing 50 mL 0.05 X MMR and 0.75 mg/L MK-801 for 2 h before evaluation in the Y-maze.

Molecular biology

Mutations in *GRIA1* (MIM: 138248) were introduced by site-directed mutagenesis into their corresponding positions in rat cDNA expression constructs encoding GluA1. Specifically, the plasmid vector pXOOF[38] containing cDNA for the unedited flip isoform of rat *GRIA1* was used for site-directed mutagenesis and subsequent heterologous expression in mammalian cells or generation of mRNA for microinjection in *Xenopus laevis* oocytes (XOs). Site-directed mutagenesis was performed with the QuickChange mutagenesis kit (Stratagene, La Jolla, CA). The mutations were verified by Sanger DNA sequencing of the entire GluA1-coding region (GATC Biotech, Constance, Germany). For analysis of cell-surface expression of WT and mutant GluA1 receptors, cDNA encoding a β-lactamase (blac) enzyme was inserted in the GluA1 cDNA in between the segments encoding the N-terminal signal sequence and the NTD with In-Fusion cloning (Promega, Mountain View, CA, USA). Specifically, a PCR-amplified DNA fragment encoding blac flanked by two short amino acid linkers (GGSGS and GGSG) was inserted in-frame between the signal sequence and the NTD with an *Xho*I restriction site introduced by site-directed mutagenesis of codon 24 and 25 to create WT and mutant blac-GluA1 constructs. For imaging of the expression patterns of WT and mutant GFP-tagged GluA1 subunit, a GFP-tagged GluA1 construct[39] and the red fluorescent membrane-reporter mCardinal-Farnesyl-5 were PCR subcloned into the pmLINK plasmid vector.[40] Two pmLINK plasmids can be fused by a two-step recombination strategy to form an expression construct for co-expression of cDNA inserts from two identical CMV promoter expression cassettes.[40] Specifically, pMLink-eGFP-GluA1-WT and pMLink-eGFP-GluA1-Arg377Ter were digested with *Swa*I and combined with *Pac*I-digested pMLink-mCardinal-Farnesyl-5 by use of Gibson assembly (Gibson Assembly Cloning Kit, New England Biolabs) for generation of pMLink-eGFP-GluA1-WT-mCardinal-Farnesyl-5 and pMLink-eGFP-GluA1-Arg377Ter-mCardinal-Farnesyl. For co-expression studies of WT and mutant GluA1 with the AMPAR GluA2 subunit and the auxiliary TARP-class subunit gamma-2, pXOOF plasmid constructs containing the flip isoform of rat GluA2 in the R-edited form (GluA2R) and gamma-2 were used. When used as templates for *in vitro* transcription of mRNA, all pXOOF plasmid constructs were linearized downstream of the 3' untranslated region with *Nhe*I, purified with a NucleoSpin DNA clean-up kit (Macherey-Nagel, Düren, Germany), and stored at a concentration of 1.0 μg/μL at −20°C until use. cRNA transcription was performed with the ARCA mRNA synthesis kit (NEB, Madison, WI, USA). The resulting mRNA was column purified with NucleoSpin RNA Clean-up kit (Macherey-Nagel), diluted to 0.5 ng/nL, and stored at −80°C until use.

Mammalian cell culturing and expression

HEK293T cells (American Type Culture Collection, Manassas, VA) were cultured in DMEM supplemented with 10% v/v fetal bovine serum, 100 units/mL penicillin, and 100 μg/mL streptomycin at 37°C in a humidified 5% CO_2 environment. For expression of WT and mutant GluA1 in HEK293 cells, TransIT-LT1 DNA transfection reagent (Mirus, Madison, WI) was used as described previously.[38] Briefly, HEK293 cells in suspension (1e6 cells/mL) were mixed with DNA/transfection complex formed by mixing plasmid DNA, TransIT-LT1 reagent, and DMEM in a 1:3:30 ratio and immediately plated into poly-*D*-lysine coated glass-bottom 96-well plates (MatTek Corporation, Ashland, MA) at 1e6 cells and 1 μg plasmid DNA per well and incubated for 2 days before experiments.

Xenopus laevis oocyte expression

Defolliculated XOs (stage V to VI) were prepared and injected with mRNA as described previously.[41] The care and use of *Xenopus laevis* were in strict adherence to a protocol (license 2014−15−0201−00031) approved by the Danish Veterinary and Food Administration. Injected XOs were incubated at 18°C in Modified Barth's Solution (MBS) containing (in mM) 88 NaCl, 1 KCl, 0.41 $CaCl_2$, 2.4 $NaHCO_3$, 0.33 $Ca(NO_3)_2$, 0.82 $MgSO_4$, 5 Tris (pH 7.4) supplemented with 50 μg/mL gentamycin until use.

Cell surface expression levels

Relative levels of surface-expressed blac-tagged GluA1 were quantified in living cells by measuring the conversion rate of the membrane-impermeable blac substrate nitrocefin by simple absorption spectroscopy.[42] For analysis in XOs, injected oocytes were placed individually in wells of clear-bottom 96-well plates containing 100 μL MBS followed by the addition of nitrocefin to a final concentration of 50 μM in a total volume of 200 μL per well and incubated at 37°C for 3 h. During the incubation time, 50 μL samples of medium were removed every 30 min, and the absorbance at 486 nm of the nitrocefin conversion product was determined with a microplate reader (Safire2, Tecan, Maennedorf, Switzerland), plotted as a function of time of sampling, and the rate of nitrocefin conversion was determined by linear regression analysis of the slope of the curve in the linear range.

Immunoblot analysis

Transfected HEK293 cells lysate was mixed 1:1 with 2× SDS sample buffer composed of 50 mM Tris-HCl, 2% w/v SDS, 10% w/v glycerol, 1% w/v β-mercaptoethanol, 12.5 mM EDTA, 0.02 w/v % bromophenol blue (pH 6.8) and heated at 65°C for 5 min. 20 μL samples were loaded on freshly prepared 10% polyacrylamide gels, resolved on the basis of molecular weight through electrophoresis, and transferred to a polyvinylidene difluoride membrane (Sigma-Aldritch) and incubated with antibodies for GluA1 for 2 h at room temperature (RT), washed twice for

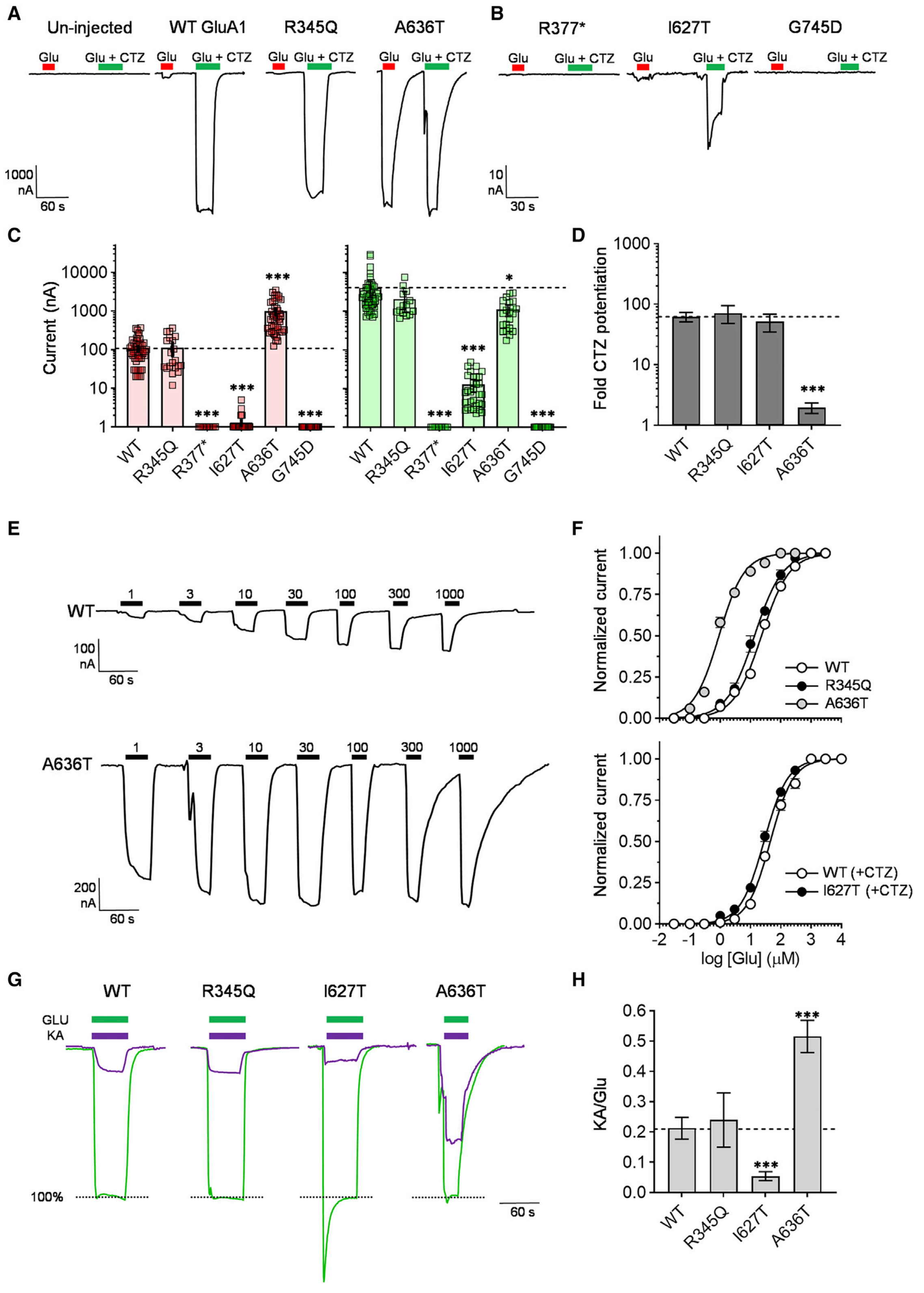

Figure 3. Functional characterization of homomeric mutant GluA1 receptors
(A and B) Representative steady-state currents evoked by sequential 10–20 s applications of Glu (1 mM, red bar) and Glu in the presence of CTZ (100 μM, green bar) from un-injected oocytes and oocytes expressing WT and mutant GluA1. The holding potential was −40 mV in all shown recordings. Note that the p.Arg377Ter, p.Ile627Thr, and p.Gly745Asp variants (indicated by R377*, I627T, and G745D, respectively) (B) show no or very small currents relative to WT, p.Arg345Gln, and p.Ala636Thr.

(legend continued on next page)

15 min at RT, then incubated with alkaline phosphatase-conjugated secondary antibody for 2 h at RT, and finally rinsed in water for 15 min. The immunoreactive protein content in the membrane was visualized with alkaline-phosphatase-mediated conversion of 5-bromo-4-chloro-3-indolyl phosphate (BCIP)/nitro blue tetrazolium (NBT) into an insoluble blue-purple product (SIGMAFAST BCIP/NBT system, Merck).

Two-electrode voltage clamp (TEVC) electrophysiology

Glass micropipettes (0.69 mm ID/1.2 mm OD, Harvard Apparatus, Holliston, MA) were pulled on a Sutter P-1000 micropipette puller (Sutter Instruments, Novato, CA) to a tip resistance of 0.5–2.5 MΩ and filled with 3 M KCl. Oocytes were clamped with a two-electrode voltage-clamp amplifier (OC-725C, Warner Instruments, Hamden, CT) and continuously perfused with Frog Ringer's solution containing (in mM) 115 NaCl, 2 KCl, 5 HEPES, and 1.8 $BaCl_2$ (pH 7.6 with NaOH) by gravity-assisted perfusion at flow rates of 2–4 mL/min into a vertical oocyte flow chamber with a volume of 0.3 mL.[43] Compounds were dissolved in Frog Ringer's solution and added by bath application. Concentration-response data were generally recorded at holding potentials of −40 mV; otherwise, they were recorded in the −20 to −80 mV range. Each compound solution was applied for 10 to 60 s depending on time needed to obtain steady-state currents. Data acquisition was accomplished with a CED 1401plus analog-digital converter (Cambridge Electronic Design, Cambridge, UK) interfaced with a PC running WinWCP software (available from Strathclyde Electrophysiology Software, University of Strathclyde, Glasgow, UK). Concentration-response experiments were performed by measuring agonist-evoked current during stepwise application of increasing concentrations of agonist, as illustrated in Figure 3E. All experiments were performed at RT.

Confocal imaging

A Leica SP2 confocal microscope equipped with an argon laser, a helium/neon laser, and 63X 1.2 Na HCX PL APO water-corrected objective was used. GFP-tagged WT and mutant GluA1 were visualized with 488 laser lines at 25%–35% input power as excitation sources and emission measurement in the 500–560 nm spectrum ranges. In addition, the co-expressed plasma membrane marker mCherry-Farnesyl-5 was visualized with the 633 nm helium/neon laser line at 25%–35% input power and collection of emission in the 640 to 700 nm spectrum range. Overlay images were produced with Leica LAS AF Lite software (Leica Microsystems GmbH, Wetzlar, Germany).

Data and statistical analysis

To construct concentration-response curves from electrophysiological data, we determined agonist-evoked current responses from individual oocytes from TEVC traces by using ClampFit 10 software (Molecular Devices, San Jose, CA) and normalized them to the current response by maximal agonist concentration. Composite concentration-response plots were constructed from normalized responses from 8 to 30 oocytes and fitted with GraphPad Prism v6.01 (GraphPad Software, San Diego, CA, USA) to a four-variable Hill equation:

$$\text{response} = +\frac{-\ \text{bottom}}{1+10^{(logEC_{50}-X)\cdot nH}}.$$

In this equation, *bottom* is the fitted minimum response, *top* is the fitted maximum response, *nH* is the Hill slope, X is the agonist concentration, and EC_{50} is the half-maximally effective agonist concentration. All statistical analyses of data from TEVC experiments were performed in GraphPad Prism 9. Unless otherwise stated, summary TEVC data are represented as mean with 95% confidence interval (CI) from n cells. One-way analysis of variance (ANOVA) with Dunn's *post hoc* multiple comparison test was performed for comparisons of three or more groups in which the data were normally distributed and where a p value < 0.05 was considered significant.

For the FMP Y-maze experiment, data across groups were compared with an analysis of covariance (ANCOVA) with significant effects assessed by Dunn's *post hoc* multiple comparison test, where a p value < 0.05 was considered significant. Grouped data are shown as the mean ± the standard error of the mean (SEM). Unless otherwise stated, statistical significance was denoted as follows: *$p < 0.05$, **$p < 0.01$, and ***$p < 0.001$.

Results

Genetic and clinical findings in individuals with homozygous and heterozygous GRIA1 variants

Seven unrelated individuals diagnosed with NDD and with rare *GRIA1* variants were identified and included in the study via the author's clinical practice, direct communication, and through the GeneMatcher[21] or ClinVar database.[22] In all cases, the *GRIA1* variants were identified from clinical or research genetic analysis aimed to determine the genetic cause underlying the individual's NDD and were found to include four missense variants (c.1906G>A [p.Ala636Thr], c.1880T>C [p.Ile627Thr], c.2234G>A [p.Gly745Asp], and

(C) Scatter plot with bars of individual and mean amplitude of Glu-evoked currents in oocytes expressing WT and mutant GluA1 in the absence (red bars and symbols) and presence (green bars and symbols) of CTZ block of desensitization. Error bars indicate the 95% confidence interval of the mean amplitude. Note the semi-log y axis. The stipulated line indicates the mean amplitude level for WT GluA1.
(D) Summary of fold desensitization for Glu-evoked currents calculated from the amplitudes of currents evoked by sequential application of Glu in the absence and presence of CTZ. Data represent the mean of 10–50 oocytes for each WT and mutant GluA1. Error bars indicate the 95% confidence interval of the means.
(E) Representative steady-state currents evoked by sequential applications (black bars) of increasing concentrations of Glu at oocytes expressing WT and the p.Ala636Thr variant GluA1.
(F) Composite concentration-response curves for WT and mutant GluA1 homomeric receptors. Data points represent the mean of 10–31 oocytes. Error bars are the SEM and are shown when larger than symbol size. The current responses are normalized to the maximal response evoked by Glu (1 mM).
(G) Overlay of representative steady-state currents evoked by sequential applications of Glu (1 mM, green bars) and KA (300 μM, purple bars) in the presence of CTZ (100 μM) to show the efficacy of KA relative to Glu for evoking current. The KA currents are shown normalized to the Glu current.
(H) Summary of the average KA/Glu current response ratios for WT and mutant GluA1. Error bars indicate the 95% confidence interval of the means.

Table 1. Variant information

Affected individual	Variant	GRCh38	cDNA	CADD	SIFT	PolyPhen2	gnomAD requency
1	p.Arg377Ter	g.153686324C>T	c.1129C>T homozygous	36.0	–	–	0
2–4	p.Ala636T	g.153764516G>A	c.1906G>A heterozygous	29.3	deleterious	probably damaging	0
5	p.Gly745Asp	g.153770379:G>A	c.2234G>A heterozygous	28.1	deleterious	probably damaging	0
6	p.Ile627Thr	g.153764490T>C	c.1880T>C heterozygous	28.5	deleterious	possibly damaging	0
7	p.Arg345Gln	g:153,686,229:G>A	c.1034G>A heterozygous	21.8	tolerated	benign	1.24 × 10e−5

The table shows the five *GRIA1* variants identified in subjects, the resultant change in amino acid, the genomic DNA nucleotide change in *GRIA1*, and the site of the variants in cDNA encoding the GluA1 subunit protein. Combined annotation-dependent depletion (CADD) scores[23] predicted that variants p.Arg377Ter, p.Ala636Thr, p.Gly745Asp, and p.Ile627Thr are highly likely to be deleterious variants. Sorting Intolerant From Tolerant (SIFT)[24] and Polymorphism Phenotyping v2 (PolyPhen2)[25] analysis predicted all variants to be deleterious or damaging, except for p.Arg345Gln, which is predicted as tolerated or benign. Note that the p.Ala636Thr, p.Ile627Thr, and p.Gly745Asp variants (in individuals 2, 5, and 6, respectively) were reported previously.[14–16]

c.1034G>A [p.Arg345Gln]) and one truncating variant (c.1129C>T [p.Arg377Ter]) (Table 1). The p.Ile627Thr, p.Ala636Thr, and p.Gly745Asp missense variants were heterozygous and arose *de novo*. The p.Arg345Gln variant was also heterozygous, but inheritance information was lacking for this variant. The truncating stop-gain variant was inherited as autosomal recessive homozygous variants from consanguineous parents. Three individuals harbored the p.Ala636Thr variant (individuals 2–4), which previously has been reported as a recurrent *de novo GRIA1* variant.[16] An overview of the genetic and bioinformatic data is provided in Table 1, while the clinical features of the individuals are provided in Table 2. Individual case stories are included in the supplementary information (see supplemental note). The ages of the individuals ranged from 7 to 26 years and included an equal number of males and females and one individual for which sex was not reported (Table 2). All individuals were diagnosed with neurodevelopmental delay. In addition, individuals were most severely affected in terms of verbal abilities: individuals 1, 2, and 3 remained non-verbal while individuals 4 and 6 communicated with either simple words or short sentences. Data on speech was not available for individuals 5 and 7. All individuals could walk independently before 24 months of life (ranging from 12 to 17 months). Individuals 1, 2, 3, 4, and 6 had cognitive impairment ranging from moderate to severe based on clinical assessment. As a result of limited data access, it was not possible to determine the severity of cognitive impairment for individuals 5 and 7. Also, data on behavioral phenotype were not available for subjects 5 and 7 but were reported in all remaining individuals. These included recurring issues, such as anxiety, autism spectrum disorder (ASD), and ADHD phenotypes (Table 2). Individual 6 was described as having a low anger threshold and had challenging behavior such as anger tantrums and occasional aggressive outbursts; however, no self-injurious behavior was described. Individuals 3, 4, and 6 were reported to have ASD. Individuals 1, 3, and 4 were reported to have a sleeping disorder necessitating treatment with melatonin. Normal brain MRI was reported for all individuals except individual 7, for which information on MRI status was not available. None of the individuals were reported to have a movement disorder. Epileptic seizures were only reported in individual 1, who carried the homozygous truncating variant and were reported to be treatment-resistant focal seizures (supplemental note). An endocrine disorder was identified in individuals in the forms of premature puberty in affected individual 1 and hypothyroidism plus polycystic ovarian syndrome in affected individual 6.

Evaluation of effects of *GRIA1* variants on GluA1 subunit expression

The amino acid residues in the GluA1 subunit protein affected by the *GRIA1* missense variants in individuals 1 to 7 are located in different subunit domains (Figures 1A and 1B). As shown in the cartoon representation of a single GluA1 subunit protein (Figure 1A), Arg345 is located in the NTD, Gly745 is located in the ABD that contains the Glu-binding site, and Ile627 and Ala636 are located within the M3 transmembrane domain of the TMD. The Arg377 residue affected by the stop-gain variant in individual 1 is located in the C terminus of the NTD close to the linker that connects the NTD to the ABD (Figure 1A). The stop-gain variation (p.Arg377Ter) is therefore predicted to cause expression of the NTD alone. All of the residues are located in GluA1 sequence regions that are highly conserved among species and the other AMPAR subunits GluA2–4 (Figure 1B). Except for Arg345, no other missense variants affecting the residues exist in the Genome Aggregation Database (gnomAD), which may indicate sensitivity to missense variation. The p.Ile627Thr, p.Ala636Thr, and p.Gly745Asp variants that were not present in gnomAD were classified as potentially damaging by various *in silico* tools for the prediction of deleteriousness of single missense variants (Table 1). Moreover, missense tolerance ratio (MTR) analysis[6] of the *GRIA1* coding sequence reveals the codons encoding Ile627 and Ala636 to have MTR scores (0.41 and 0.33, respectively) that are in the lower 5% percentile and close to the global minimum of the entire subunit sequence (Figure 1D), strongly indicating an unusually high sensitivity of these residues to missense variation. Gly745 also has a low MTR score (0.66) and is located in a local minima region of the MTR (Figure 1D) and, therefore, is also predicted to have sensitivity to missense variants. In contrast, Arg345 is located in a

Table 2. Clinical features of individuals harboring GRIA1 variants

Individual	Individual 1	Individual 2	Individual 3	Individual 4	Individual 5	Individual 6	Individual 7
**Transcript number**	GenBank: NM_000827.3	GenBank: NM_000827.3	GenBank: NM_000827.3	N GenBank: M_000827.3	GenBank: NM_000827.3	GenBank: NM_000827.3	GenBank: NM_000827.3
***GRIA1* variant**	c.1129C>T (p.Arg377Ter)	c.1906G>A (p.Ala636Thr)	c.1906G>A (p.Ala636Thr)	c.1906G>A (p.Ala636Thr)	c.1880T>C (p.Ile627Thr)	c.2234G>A (p.Gly745Asp)	c.1034G>A (p.Arg345Gln)
**Current age**	10	7	13	26	N.R.	21	N.R.
**Sex**	female	female	male	female	male	female	N.R.
**Intellectual disability (ID)**	yes	yes	yes	yes	yes	yes	yes
**Level of cognitive impairment based on the clinical impression**	severe	severe	severe	moderate	not classified	moderate	not classified
**Level of speech impairment**	non-verbal	non-verbal	non-verbal	language difficult to understand	N.R.	simple verbal language	N.R.
**Motor development**	walked at 14 months	walked at 12 months; sat at 12 months	walked at 18 months; sat at 5 months	walked at 17 months	N.R.	Walked at 13 months; delayed fine motor skills and coordination issues	N.R.
Epilepsy diagnosis	yes	N.R.	no	no	no	no	no
Electroencephalogram (EEG)	frequent interictal epileptiform discharges with spikes/spike over posterior regions	N.R.	N.R.	N.R.	N.R.	normal	N.R.
**Other movement disorder**	tip-toe walking	N.R.	N.R.	No	N.R.	dystonia, catatonia	N.R.
**Behavioral issues**	self-injurious behavior	unspecified behavioral problems	ADHD ASD	ADHD	unspecified behavioral problems	anxiety; anger tantrums; ASD	N.R.
Brain MRI	normal	normal	normal	normal	normal	normal	N.R.
Sleep	poor sleep requiring melatonin	N.R.	poor sleep requiring melatonin	normal	normal	poor sleep	N.R.
**Vision**	left intermittent divergent squint	N.R.	possible squint, not diagnosed	hypermetropia astigmatism glasses at 11 months	N.R.	normal	N.R.
Dysmorphic features	no	mild upslanting palpebral fissures	broad forehead and telecanthus	no	flushed cheeks and ears; high arched palate	normal	N.R.

(*Continued on next page*)

Table 2. *Continued*

Individual	Individual 1	Individual 2	Individual 3	Individual 4	Individual 5	Individual 6	Individual 7
Endocrine/metabolic disease	precocious puberty from 6 years of life	N.R.	no	no	no	hypothyroidism polycystic ovarian syndrome	N.R.
Cardiovascular disease	N.R.	N.R.	N.R.	no	bicuspid aortic valve	no	N.R.
**Head circumference (cm)**	49	50	53	N.R.	N.R.	N.R.	N.R.
**Age at physical assessment (years)**	9 $^{1}/_{2}$	5 $^{1}/_{2}$	4	26	N.R.	19 $^{1}/_{2}$	N.R.
**Weight (kg)**	31	26	21	60	N.R.	54	N.R.
**Height (cm)**	140.5	118	107.3	149	N.R.	157.5	N.R.
**Other genetic findings**	normal *SLC2A1* and epilepsy and severe delay gene panel	normal 250k SNP array and *FMR1* analysis	N.R.	normal array CGH	N.R.	N.R.	N.R.

ASD, autism spectrum disorder; ADHD, attention deficit hyperactivity disorder; ID, intellectual disability; N.R., not reported. Some information for individuals 2 and 5 were reported previously.[14,16]

sequence region without reported unusual sensitivity to missense variants.

For evaluation of the effect of the *GRIA1* variants on expression and function of GluA1-containing AMPARs subtypes, the mutations reflecting the *GRIA1* variants p.Arg345Gln, p.Arg377Ter, p.Ile627Thr, p.Ala636Thr, and p.Gly745Asp were generated in the GluA1 subunit (material and methods). Mutational effects on the ability of the GluA1 subunit protein to fold correctly, assemble into receptors, and traffic to the cell surface membrane were first characterized with a β-Lactamase (β-lac) enzyme reporter assay.[38,44,45] Specifically, β-lac was fused to the extracellular N terminus of WT and mutant GluA1. The resulting β-lac-tagged subunit constructs were expressed in *Xenopus laevis* oocytes (material and methods). Following 2 days of expression, the relative levels of WT and mutant receptor present at the oocyte cell surface were determined by measuring the rate of β-lac catalyzed cleavage of the cell-impermeable chromogenic substrate nitrocefin added to the cells.[42,45] All mutants except p.Arg377Ter displayed β-lac activity similar to WT GluA1, which indicates that the variations do not affect normal efficiency of subunit expression, folding, assembly, and surface trafficking (Figure 1E). In contrast, p.Arg377Ter-expressing oocytes showed a similar level of β-lac activity to un-injected oocytes (0.002 OD/h versus 0.001 OD/h; $p = 0.72$; Figure 1E), supporting that the p.Arg377Ter variation truncates the GluA1 subunit in the NTD such that the subunit lacks the ABD and TMD that is essential for subunit assembly into functional receptors in the membrane (Figure 1). This was further tested by expressing WT GluA1 and the p.Arg377Ter mutant tagged with GFP in the N terminus of the NTD in HEK293 cells together with the red-fluorescent cell surface membrane reporter protein construct mCardinal-farnesyl (material and methods). Confocal imaging of GFP fluorescence in the transfected cells showed clear membrane localization of WT GFP-GluA1 protein that overlapped with mCardinal-farnesyl fluorescence. In contrast, GFP fluorescence for GFP-GluA1-Arg377Ter cells was confined to the intracellular compartments (Figure 1F). Furthermore, immunoblot analysis of GluA1 protein size in protein extracts from WT and p.Arg377Ter-transfected HEK293 cells were performed (Figure 1G). Analysis with an antibody directed against an NTD epitope showed GluA1 protein with a size corresponding to full-length GluA1 (100 kDa) in WT transfected cells, whereas p.Arg377Ter-transfected cells showed a protein product with a size corresponding to truncation at Arg377 (43 kDa). A similar analysis with an antibody directed at the GluA1 C terminus showed a 100 kDa size for the WT protein and detected no GluA1 protein in extracts from p.Arg377Ter-transfected cells (Figure 1G), indicating that translational read-through of the stop codon generated by the p.Arg377Ter variant does not occur. Together, these data show that the homozygous *GRIA1* variant p.Arg377Ter identified in individual 1 is a stop-gain variant that truncates GluA1 at Arg377 and prevents

any expression of functional GluA1 subunit in human and amphibian cells.

Indels in exon 8 cause working memory deficits in tadpoles

We modeled the homozygous stop-gain variant p.Arg377Ter in *Xenopus tropicalis* tadpoles by using CRISPR-Cas9 to investigate how the disrupted expression of the GluA1 subunit contributes to neurological development and behavior. Gene editing in *Xenopus* species is now so efficient that analysis is routinely performed in founder animals, enabling rapid testing to support the causality of genetic disruption across a range of genes.[46–49] To support the use of *X. tropicalis* to model variants in *gria1,* both human and *Xenopus tropicalis* share identical gene structures and produce proteins that are >87% conserved (Figures S1A and S1B). Additionally, the exon 8 target region corresponding to the homozygous nonsense variant identified in individual 1 (p.Arg377Ter) is well conserved in *Xenopus* (Figure S1C). We used CRISPR-Cas9 editing (material and methods) to disrupt exon 2 (*Xtr.gria1*$^{\mathrm{em1EXRC}}$, *gria1* knockout) or the genomic DNA encoding 18 amino acids upstream of Arg377 in exon 8 (*Xtr.gria1*$^{\mathrm{em2EXRC}}$, *gria1* crispant) (Table S1 and Figure S1). Arg377 is the penultimate amino acid of exon 8, and we chose targeting upstream of the variant to avoid altered splicing outcomes in the crispant model. Sanger sequencing of the target region within exon 8 in genomic DNA samples collected from crispant tadpoles demonstrated a good penetrance of indels. Further, sequencing of subcloned genomic amplicons revealed that none of the sequenced clones represented the WT allele and demonstrated that half of the sequenced clones truncated the protein (denoted by the asterisk, Figure 2A). The genotype of the *gria1* knockout model targeting exon 2 is predominantly a 7 bp deletion (>75% indels, Figure S2A).

All phenotyping experiments were replicated in embryos from at least three different females, and no consistent early developmental abnormalities were noted across these experiments. Transient motor differences were seen in almost all post-hatching knockout (27 of 30 animals) and crispant tadpoles (23 of 30 animals). Both types of *gria1* mutants were slower to hatch than controls and subsequently displayed an abnormal escape response to tactile stimulation. Post-hatching control animals were observed to move away from all tactile stimuli (trunk and head), whereas both types of mutant animals responded either by moving in tight circles or not at all. At later stages (NF42 onwards), all tadpoles were seen to develop normally, adopting an appropriate filter-feeding (head down, tail up) posture with the ability to navigate their environment freely. Imaging revealed no obvious or consistent craniofacial abnormalities in either model (Figures 2B and S2B). Similarly, we noted no significant gross structural differences in the forebrain, midbrain, or hindbrain regions when we made *gria1* knockout (Figure S2C) or *gria1* crispant tadpoles (Figure 2C) in a tubb2bGFP background to enable visualization of the brain. A small number of tadpoles in each batch were observed to demonstrate episodic periods of abnormal behavior (5 of 50 animals), which included "C-shaped" alternating axial contractions of the tail coupled with rapid changes in direction. These "manic" bouts were followed by a prolonged and unusual period of immobility. Interestingly, this behavior is consistent with descriptions in the literature of seizures in *Xenopus* tadpoles.[50,51]

All individuals in this study have varying degrees of cognitive impairment (Table 2 and supplemental note). Until now, quantitative measures of higher executive functions in *Xenopus* have not been described. Therefore, a novel FMP behavioral model was developed in *Xenopus tropicalis* tadpoles to assess the impact of *gria1* knockout on cognitive functions (material and methods). Previous FMP Y-maze studies show a dominant vertebrate search strategy largely consisting of alternating left/right choice patterns (LRLR or RLRL). Importantly, this strategy can be impaired by pharmacological agents that disrupt working memory and cognitive flexibility.[34,35] The results presented here show that control tadpoles demonstrate a predominant search strategy consisting largely of alternations (black bars, Figures 2D, S2D, and S3A). The alternation strategy observed in control tadpoles was abolished following administration of the NMDA receptor antagonist MK-801 (green bars, Figure S3). Specifically, MK-801-treated tadpoles showed "primitive" search patterns consisting of repetitions (Figures S3A and S3D), similar to those observed in invertebrate models.[34] These findings are consistent with those reported in rodent and zebrafish FMP Y-maze studies,[34,52,53] which show that glutamatergic disruption impairs spatial working memory and provides a further demonstration of the suitability of *Xenopus* tadpoles to study genetic disruption within *gria1*.

Next, we used the 1 h FMP Y-maze assay to compare WT and *gria1* crispant (Figures 2D–2G) or *gria1* knockout tadpoles (Figures S2D–S2G). Both the *gria1* crispant (Figure 2D) and *gria1* knockout tadpoles (Figure S2D) showed a significant decrease in alternations compared to WT tadpoles. This decrease in alternations was present across the entire 1 h trial (Figures 2E and S2E) and is consistent with a working memory deficit. These results agree with the finding of short-term working memory deficits in the *gria1*$^{-/-}$ mouse model.[54–58] Unlike in the *Gria1*$^{-/-}$ mouse, there was no evidence to support hyperactivity in either type of mutant tadpole (Figures 2F and S2F). However, caution must be applied to this conclusion because these assessments were made on the movement into and out of zones rather than the total distance covered. Finally, the tadpoles show a relatively static search strategy and appear to perform a decreasing number of turns across the trial, suggesting that the 1 h trial period could be reduced in future studies (Figures 2G and S2G).

Table 3. **Functional parameters for WT and mutant GluA1-containing AMPAR subtypes**

Receptor	Mean current (nA)	n	Mean current with CTZ (nA)	n	Glu EC50 (uM)	n	Glu EC50 with CTZ (uM	n	Fold desensitization	n	KA/Glu (%)	n
**WT**	104 (81–127)	53	4,074 (2,316–5,832)	45	23 (21–25)	25	46 (40–51)	18	62 (51–72)	21	21 (18–25)	36
+ A2R	511 (372–651)	54	7,051 (4,631–9,472)	26	18 (17–18)	31	n.d.	–	57 (46–66)	19	30 (27–32)	25
+ A2R, + γ-2	1,474 (1,028–1,919)	53	7,930 (4,350–11,509)	23	14 (12–17)	11	n.d.	–	7.2 (5.6–8.7)	46	74 (69–78)	27
**R354Q**	114 (63–164)	20	2,081 (524–1,959)	14	14 (12–16)	10	n.d.	–	71 (48–94)	15	24 (17–32)	9
+ A2R	255 (179–331)	26	3,587 (2,187–4,987)	10	9.4 (8.4–11)	13	n.d.	–	38 (26–48)	9	30 (27–35)	16
+ A2R, + γ-2	1,001 (720–1,282)	43	4,433 (2,637–6,230)	16	5.9 (4.7–7.2)	12	n.d.	–	5.9 (4.3–7.5)	16	80 (73–87)	16
**p.Arg377Ter**	0*** (0–0)	10	0***	10	n.d.	-	n.d.	–	n.d.	–	n.d.	–
+ A2R	0*** (0–0)	10	0***	10	n.d.	–	n.d.	–	n.d.	–	n.d.	–
+ A2R, + γ-2	0*** (0–0)	10	22*** (2–40)	10	n.d.	–	n.d.	–	n.d.	–	n.d.	–
**p.Ile627Thr**	1*** (0–1)	26	12*** (8–17)	31	n.d.	–	28* (26–30)	20	52 (35–69)	10	5*** (4–7)	10
+ A2R	40*** (28–52)	25	2,728** (1,821–3,635)	16	15 (13–17)	10	n.d.	–	72 (47–97)	12	9*** (8–11)	17
+ A2R, + γ-2	825 (610–1,041)	41	13,505* (7,835–19,174)	9	11 (9–13)	17	n.d.	–	14 (12–18)	13	66 (61–71)	11
**p.Ala636Thr**	993*** (746–1,246)	43	1,105** (729–1,482)	22	0.9 (0.8–1.0)	26	n.d.	–	1.9*** (1.6–2.4)	24	52*** (46–56)	25
+ A2R	2064** (1,437–2,690)	27	2474** (1,636–3,313)	16	4.2 (3.9–4.6)	10	n.d.	–	1.1*** (1.0–1.3)	16	81*** (70–90)	15
+ A2R, + γ-2	2,504 (1,151–3,857)	20	1,961** (1,650–3,758)	12	5.8 (4.8–7.1)	13	n.d.	–	1.3*** (1.1–1.4)	14	65 (55–75)	11
**p.Gly745Asp**	0*** (0–1)	34	0*** (0–1)	22	n.d.	–	n.d.	–	n.d.	n.d.	n.d.	–
+ A2R	83*** (57–109)	29	347*** (191–504)	11	14 (13–16)	11	n.d.	–	6.7*** (6.0–7.0)	11	58*** (52–63)	11
+ A2R, + γ-2	219*** (134–303)	31	767*** (354–1,179)	22	5.8 (4.3–8.0)	10	n.d.	–	7.3 (5.8–8.9)	26	94*** (89–99)	11

Values are given for mean currents, Glu EC50, fold desensitization, and KA/Glu response ratios from the electrophysiological experiments as described in the text. The data represent means ± 95% confidence intervals. n, number of individual experiments or oocytes. Statistical information in the form of probability value (p) level is given where values are significantly different from WT as follows: *, $p < 0.05$; **, $p < 0.01$; ***, $p < 0.001$. CTZ, cyclothiazide; Glu, glutamate; KA, kainic acid; n.d., not determined; WT, wild type.

Impact of variants on homomeric GluA1 receptor function

The GluA1 subunit can assemble as a homomeric receptor and as heteromeric receptor subtypes with GluA2–4 subunits.[59–61] First, the effect of the *GRIA1* variants on ligand-gated ion channel function of homomeric GluA1 receptors was evaluated by measuring current responses to Glu application in *Xenopus laevis* oocytes expressing WT and the mutant GluA1 subunits (Figure 3). Like all AMPAR subtypes, homomeric GluA1 receptors display fast and profound desensitization;[1,62] resulting in current responses to Glu that, within milliseconds, decline by more than 95% from a peak response level to a steady-state level that represents the majority of the receptor population to reside in the desensitized receptor state.[63,64] This response waveform cannot be resolved in *Xenopus* oocytes. Therefore, to enable measurement of both desensitized and non-desensitized response levels, we performed recordings of Glu-evoked currents in the presence and absence of cyclothiazide (CTZ), a compound that blocks AMPAR desensitization (material and methods) (Figure 3A and 3B). Un-injected oocytes did not show any responses to Glu in absence or presence of CTZ (Figure 3A), confirming that *Xenopus laevis* oocytes do not express endogenous AMPA receptors at functional detectable levels. All mutants except p.Arg345Gln showed current responses that were significantly different from WT (Figure 3C and Table 3). As expected from the biochemical analysis of surface expression, oocytes expressing Arg377Ter did not produce current response to Glu in

the absence or presence of CTZ (Figure 3A and Table 3), further confirming that the p.Arg377Ter variant prevents the expression of a functional GluA1 subunit. Also, no detectable desensitized or non-desensitized currents were observed for the p.Gly745Asp variant (Figure 3A and Table 3). The p.Ile627Thr variant displayed detectable currents during desensitizing and non-desensitizing conditions, however, with amplitudes more than 10-fold lower than WT (Figure 3A and Table 3). These results indicate that the p.Ile627Thr and p.Gly745Asp variants identified in individuals 5 and 6, respectively, have loss-of-function effects on the function of the GluA1 subunit. In contrast, oocytes expressing the p.Ala636Thr variant on average displayed 10-fold increased currents compared to oocytes expressing WT GluA1 (Figures 3A and 3E and Table 3). Importantly, in individual p.Ala636Thr-expressing oocytes, the Glu-evoked currents recorded sequentially in the absence and presence of CTZ had near-identical amplitudes (Figure 3A), whereas currents in WT-expressing oocytes increased 61-fold when desensitization was blocked by CTZ (Figure 3D and Table 3), a factor that corresponds well to previously reported ratios between non-desensitized peak current and desensitized steady-state current amplitudes for homomeric GluA1 receptors recorded in HEK293 cells via fast-application protocols.[63,64] This result strongly indicates that the p.Ala636Thr variation disrupts the ability of homomeric GluA1 receptors to desensitize and explains the dramatic increase in current amplitude during non-desensitizing conditions. A similar analysis of the factor by which CTZ increased Glu-evoked current for p.Arg345Gln and p.Ile627Thr showed increases that were not significantly different from WT (Figure 3D and Table 3) to indicate that these variations do not change receptor desensitization properties.

In addition to changing receptor desensitization properties, variations can also change the current response to Glu by perturbing the activation properties of the receptor, e.g., the ability of the receptor to open the channel when the agonist is bound. A measure of the activation properties of AMPARs is to determine the efficacy of the weak partial agonist kainic acid (KA) relative to the full agonist Glu for activating receptor currents[65,66] (Figure 3H). When desensitization was blocked, KA evoked current at WT GluA1 that was 21% of the Glu-evoked current (Figure 3G and Table 3). The p.Arg345Gln variant showed similar KA efficacy (26%), indicating the variation does not change receptor activation properties. In contrast, p.Ile627Thr showed a significantly lower KA efficacy of 5% compared to WT, indicating a decreased ability of the GluA1 subunit to translate agonist binding to channel opening (Figure 3G and Table 3). This result explains the dramatic decrease in desensitized and non-desensitized current for p.Ile627Thr (Figure 3B). In contrast, p.Ala636Thr showed a significantly increased KA efficacy (52%) (Figure 3B and Table 3), indicating increased channel-opening ability. Notably, the effect of the p.Ala636Thr variation has previously been studied for GluA1.[67–69] Ala636 is the third Ala residue in the SYTANLAAF motif that is completely conserved in all eukaryotic iGluR subunits (Figure 1D). This motif forms the upper M3 helix that lines the extracellular entrance to the channel and acts as a gate during channel opening.[70–72] Profound effects of variation of this conserved Ala to Thr were first identified in the GluD2 iGluR in the mutant *lurcher* mice strain[73,74] that causes widespread neuronal cell death in homozygous animals. The same Ala-Thr variation has subsequently been applied in several iGluR subunits to study the role of SYTANLAAF motif for, in particular, channel activation, including in GluA1.[67,69] The results presented here corroborate previous findings that the Ala-Thr variation profoundly increases the ability of GluA1 to activate channel opening and further shows that desensitization is abolished.

We generated dose-response curves for Glu for WT and the p.Arg345Gln, p.Ile627Thr, and p.Ala636Thr variants and used them to determine the half-maximally effective concentration (EC50) of Glu (material and methods) (Figure 3E and Table 3). Because of the minimal current responses of the p.Ile627Thr variant under desensitizing conditions, the dose-response experiments for this mutant were performed in the presence of CTZ. p.Arg345Gln and p.Ile627Thr showed EC50 values identical or very close to WT (Table 3), indicating that these variations overall do not change receptor sensitivity to Glu. In contrast, the p.Ala636Thr variant showed greatly increased sensitivity towards Glu compared to WT, leading to a 25-fold reduced EC50 (Figure 3F and Table 3), which agrees with previous reports.[67–69]

Impact of variants on heteromeric GluA1 receptor function and in the presence of the auxiliary subunit γ-2

Homomeric GluA1 AMPARs are thought to exist *in vivo*;[75] however, heteromeric GluA1/A2 and GluA1/A3 receptors are considered to constitute the main population of GluA1-containing receptors in most CNS regions.[59–61,76,77] Furthermore, most native AMPARs form a complex with auxiliary subunits that regulate their function, such as the transmembrane AMPA receptor regulatory proteins (TARPs).[78] To evaluate the effects of the *GRIA1* variants in the context of heteromeric receptors, WT and mutant GluA1 were expressed together with the GluA2 subunit and the electrophysiological studies of receptor function were repeated (Figure 4). Specifically, WT and mutant GluA1 were expressed with GluA2 subunit in the R-edited (GluA2R) form in a 1:2 ratio, which previously has been shown to result in the majority of GluA1 to form a receptor population mainly composed of GluA1/A2R receptors (material and methods).[41,79] Heteromeric assembly was confirmed by measuring the current-voltage (IV) relationship of Glu-evoked currents (Figure 4B). The IV relationship changes from inwardly rectifying for homomeric GluA1 to linear when GluA1 assembles with GluA2R subunits (Figure 4B). Except for Arg377Ter, all of the mutants displayed a near-complete shift from inwardly rectifying IV curves for homomeric expression to linear IV curves when

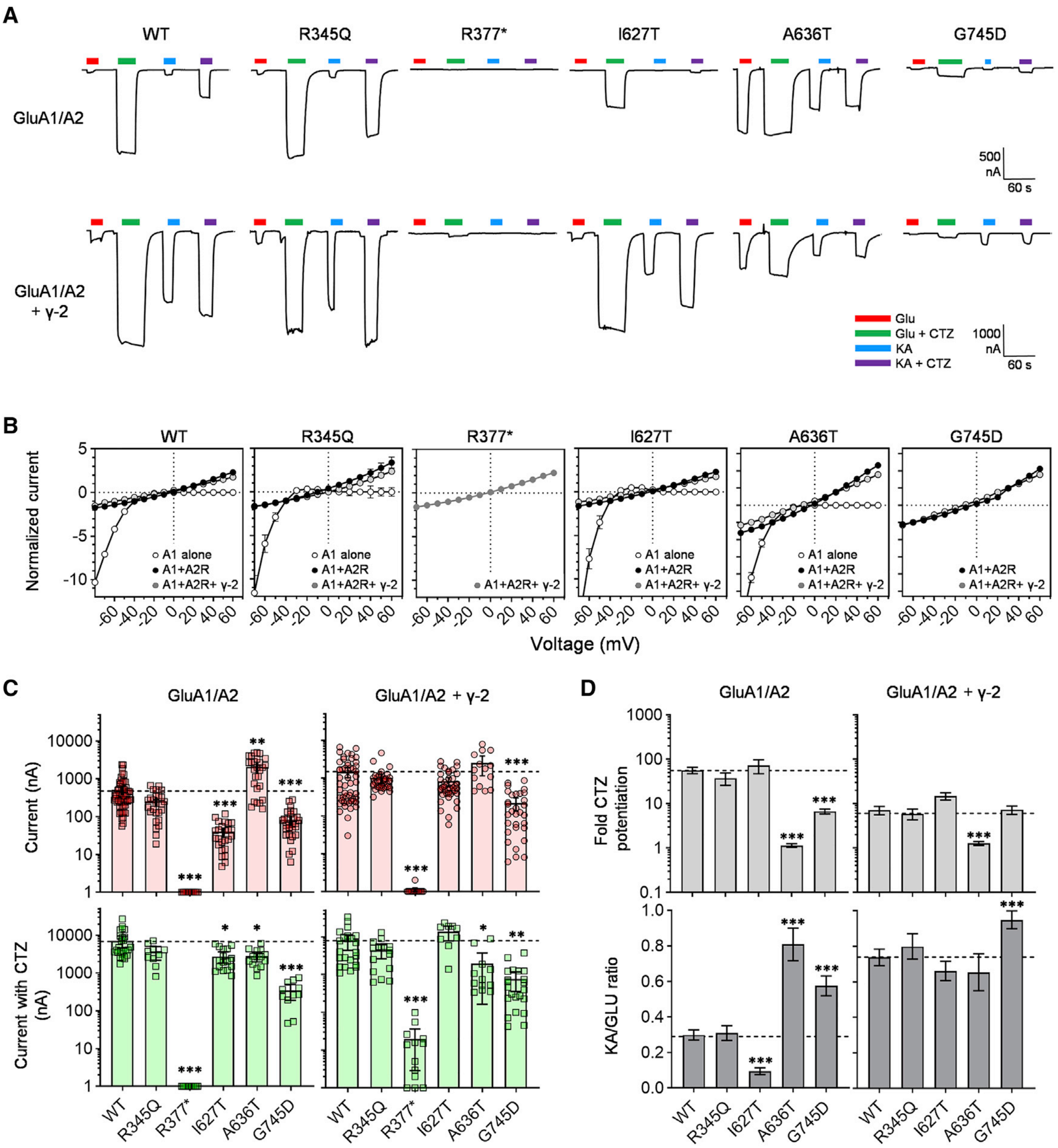

Figure 4. Functional characterization of heteromeric mutant GluA1 receptors

(A) Representative steady-state currents evoked by sequential 10–20 s applications of 1 mM Glu (red bars), 1 mM Glu in the presence of 100 μM CTZ (green bars), 300 μM KA (blue bars), and 300 μM KA in the presence of 100 μM CTZ (300 μM, purple bars), from WT and mutant GluA1/A2R receptors in the absence (upper traces) and presence (lower traces) of the TARP auxiliary subunit γ-2. The holding potential was −40 mV in all shown recordings. Note different amplitude scale for traces with and without γ-2.

(B) IV relationships of Glu-evoked currents from oocytes expressing WT and mutant GluA1 subunits alone (white circles), with the GluA2R subunit (black circles) and with the GluA2R subunit and γ-2 (grey circles). The current amplitude at the different holding potentials is shown normalized to the current at −40 mV. Data points represent the mean from six to ten oocytes. Error bars indicate the SEM and are shown when larger than symbol size.

(C) Summaries of the amplitudes of Glu-evoked currents in individual oocytes expressed recorded at −40 mV in the absence (red symbols) and presence (green symbols) of CTZ.

(D) Summaries of the average desensitization and KA/Glu ratios for WT and mutant GluA1/A2R with (right) and without γ-2 (left). Error bars indicate the 95% confidence interval of the mean.

co-expressed with GluA2R, showing that the variations do not affect the ability of GluA1 to preferentially assemble with GluA2R to form heteromeric receptors (Figure 4B). Also, mutant IV curves were identical to the equivalent WT, confirming that the variations do not change rectification properties of homo- or heteromeric receptors. Although the GluA2R subunit can form homomeric receptors, these have very low channel conductance and traffic poorly to the cell surface.[80,81] Consequently, homomeric GluA2R rarely produce detectable currents in *Xenopus* oocytes or other cells and are therefore not a concern in functional studies. In addition, to further mimic native AMPARs,

GluA1/A2 receptors were also expressed with the prototypical AMPAR auxiliary TARP-class subunit γ-2 (also known as stargazin) (material and methods).[82] Successful incorporation of γ-2 subunits into the receptor complex was determined by measuring the ratio of sequential currents evoked by GLU and KA in the absence of CTZ (Figure 4A). Previous work has shown that γ-2 increases steady-state current to KA relatively more than the Glu current, and determination of the KA/GLU current ratio provides a robust test for functional expression of AMPARs in complex with γ-2.40;[82–86] For WT and all mutants except p.Ala636Thr, γ-2 co-expression increased the KA/Glu ratio approximately 4- to 6-fold and maintained linear IV curves, verifying the formation of heteromeric GluA1/A2R receptors in complex with γ-2 (Figures 4B and S4). Notably, as the GluA1/A2-Ala636Thr receptor showed a profoundly increased KA/Glu ratio, it is likely that further increase of this ratio as measured for γ-2 incorporation is not possible.

On this background, mutant WT and mutant GluA1/A2R receptors with and without γ-2 were characterized for changes in the average current response, desensitization, and activation properties and Glu EC50 with similar electrophysiological recording protocols as for homomeric GluA1 (Figures 4A and S4). Co-expression of p.Arg377Ter with GluA2R did not produce currents, showing that the NTD-truncated GluA1 subunit cannot form a functional receptor with GluA2R. However, upon co-expression with γ-2, very small currents (10–20 nA) were detected in some oocytes (Figure 4A and Table 3), but the amplitude of the currents was more than 300-fold smaller than the mean currents in oocytes expressing WT GluA1/A2R with γ-2 and, therefore, most likely originate from homomeric GluA2R receptors, which previously have been shown to produce detectable currents when co-expressed with γ-2.40

Similar to the results from homomeric receptors, GluA1-Ile627Thr/GluA2 receptors showed currents that were significantly smaller than WT both during desensitizing conditions (40 nA for p.Ile627Thr versus 511 nA for WT; Table 3) as well as non-desensitizing conditions (2,728 nA for p.Ile627Thr versus 7,051 nA for WT; Table 3). Also similar to homomeric p.Ile627Thr, the desensitization ratio was not significantly different from WT (Figure 4D and Table 3), and the KA/Glu efficacy was decreased (9% for p.Gly745Asp versus 30% for WT; Figure 4D and Table 3). These results show that the effect of the p.Ile627Thr variation on GluA1 activation properties lead to heteromeric GluA1/A2 receptors with reduced current amplitudes. However, when WT and p.Ile627Thr was co-expressed with the γ-2 auxiliary subunit, the mean current amplitudes were not significantly different (Figures 4A and 4C; Table 3). Generally, inclusion of the γ-2 auxiliary subunit in AMPAR subtypes enhances receptor activation by increasing the efficiency of receptor subunits to translate agonist binding to channel opening,[82,87,88] and this effect is manifest as a marked increase in apparent KA efficacy.[82,89] Indeed, when γ-2 was co-expressed with the heteromeric receptors, the KA/Glu response ratio increased from 30% to 74% for WT (Figures 4A and 4D; Table 3). This effect was also observed for the p.Ile627Thr variant where KA/Glu increased from 6% to 66% (Figures 4A and 4D; Table 3). Therefore, this result suggests that inclusion of γ-2 into the GluA1/A2 receptor partly rescues the detrimental effect of the p.Ile627Thr variation on receptor activation properties. Other TARP subtypes as well as non-TARP auxiliary subunits vary greatly in their influence on AMPAR function.[78,89,90] Further work examining functional effects of AMPAR variants in the context of different auxiliary subunits may therefore be warranted.

For the p.Ala636Thr variation, the effects observed in homomeric GluA1 were maintained in heteromeric GluA1/A2R overall; these showed significantly increased Glu-evoked currents, decreased desensitization, decreased Glu EC50, and increased KA efficacy (Figures 4A, 4C, and 4D and Table 3). This effect pattern was maintained upon γ-2 co-expression, except for the KA efficacy found not to be significantly different from WT (Figure 4D). However, as previously mentioned, γ-2 in general enhances activation, and it might be speculated that this masks any effect of the p.Ala636Thr variation on activation in heteromeric GluA1/A2 receptors with γ-2.

The p.Gly745Asp variation that yielded homomeric GluA1 receptors that are completely inactive displayed desensitized and non-desensitized Glu-evoked currents when co-expressed with GluA2R (Figure 4A); however, the current amplitudes were 6-fold and 19-fold decreased, respectively, compared to the WT currents (Figure 4C and Table 3). Surprisingly, analysis of the CTZ potentiation ratio and KA/Glu efficacy ratio showed p.Gly745Asp to have decreased desensitization and increased KA/Glu efficacy ratio, which would suggest steady-state currents to be increased (Figure 4D and Table 3). The EC50 value for Glu was not changed (Figure S5 and Table 3). These effects of p.Gly745Asp were maintained when co-expressed with γ-2, except for the CTZ potentiation ratio, which was not different from WT GluA1/A2R with γ-2 (Figure 4D and Table 3). These results show that the p.Gly745Asp changes functional properties of the GluA1 subunit to overall lower heteromeric GluA1/A2R receptor currents; however, they do not unequivocally reveal how the variation disrupts function. Finally, the p.Arg345Gln variant displayed similar properties to WT GluA1/A2R with and without γ-2 with comparable current amplitudes, desensitization, and KA/Glu ratios that were not different from WT (Figure 4D and Table 3).

In summary, the electrophysiological evaluation of the effect of the *GRIA1* variants on AMPAR function confirmed the prediction that the stop-gain variant p.Arg377Ter completely prevents the formation of functional GluA1-containing AMPARs. Notably, this finding supports the results from the *Xenopus* tadpole behavioral experiments that showed the knockout and the exon 8 deletion crispant tadpoles to have indistinguishable phenotypes. Together, this suggests that the homozygous p.Arg377Ter variant leads to complete loss of GluA1 in individual 1. For the

missense variants in individuals 2 to 7, the electrophysiological results strongly suggest that the p.Ile627Thr and p.Gly745Asp variants overall lead to severe loss-of-function phenotypes for homomeric GluA1 receptors and significantly decrease heteromeric GluA1-containing receptor function. In contrast, the p.Ala636Thr variant produces a clear gain-of-function phenotype in homomeric and heteromeric receptors characterized by loss of desensitization and increased Glu sensitivity. The p.Arg345Gln variant was found not to change functional parameters of both homomeric and heteromeric receptors and therefore may be considered benign in terms of function.

Structural modeling of GluA1 shows that variants affect key receptor domains

Based on X-ray crystallography and cryoelectron microscopy (cryo-EM) structures representing the main functional states of AMPARs, the structural mechanisms that underlie receptor function is becoming increasingly well understood.[70–72,91–93] Overall, the mechanism can be described by a four-state model as illustrated in Figure 5A. The Ile627, Ala636, and Gly745 residues where the *GRIA1* variants lead to altered receptor function are located in regions of the receptor structure that hold critical roles in this model. Specifically, Ile627 and Ala636 are located in the upper part of the M3 helix, which lines the ion channel and contains the channel gate (Figure 5A). Gly745 is located in the clamshell-shaped ABD containing the Glu-binding site and that undergoes the initial conformational changes leading to channel opening and receptor desensitization (Figure 5A). To understand how variation of these residues can influence the stability of the key receptor states, the structural role of these residues was analyzed with homology models of homomeric GluA1 created from AMPAR structures that represent the resting, active, and desensitized receptor states (supplemental methods) (Figures 5B–5D).

Gly745 is located in the center of a short beta-strand that acts as a hinge between the upper and lower domains (denoted D1 and D2) that form the clamshell-shaped ABD (Figures 5A–5B). Upon agonist binding, D1 and D2 close around the agonist, leading to a pre-active, closed state from which the receptor can transition to an active, open-channel state or a desensitized, closed-channel state (Figure 5A). In general, glycine adds flexibility to the peptide backbone that is lost upon substitution with any residue. Therefore, it can be speculated that the p.Gly745Asp variation changes hinge properties to destabilize the closed-cleft ABD conformation in the pre-active, active, and desensitized states. Also, in the tetrameric AMPAR complex, the four ABDs are arranged pairwise into two identical dimers. In each dimer, the two ABDs are in a back-to-back orientation with the agonist binding clefts facing outwards (Figures 5A and 5B). Gly745 is part of the dimer interface that is formed mainly between the D1 subdomains (Figure 5B). In the resting and active state GluA1 models, the side chain introduced by any variation of Gly745 will face into a hydrophobic motif formed by side chains of Ile495, Pro508, and Leu765 that contributes to the D1/D1 interface (Figure 5B, lower panels). The p.Gly745Asp variation introduces an acidic side chain towards this motif, thereby possibly destabilizing the interface (Figure 5B). Notably, the strength of the D1/D1 interface is important for the stability of the pre-active and active states, and perturbation is known to be determinant for the equilibrium between receptor states[70,94–96](Figure 5A). Specifically, variations that destabilize the interface promote entry into the desensitized, closed-channel state and prevent entry into the active, open-channel state.[97] Therefore, the loss-of-function effects of the p.Gly745Asp variation may also be caused by the destabilization of the D1/D1 interface. Notably, in heteromeric receptors, such as GluA1/A2R receptors, the ABD dimers form between GluA1 and GluA2R subunits. Thus, the ABD interface in both dimers will be affected by the GluA1-p.Gly745Asp variation, explaining the dominant effect of the variation in heteromeric GluA1/A2R receptors.

Ile627 and Ala636 are located in the transmembrane M3 helix that forms the ion channel pore and the channel gate (Figures 5C and 5D). Ala636 is at the extracellular-facing tip of M3 that contains the channel gate and undergoes conformational changes during channel opening and closing.[71,72,93] The GluA1 models show Ala636 in the four subunits to be arranged in nearly identical conformations in the resting and desensitized states, where the channel gate is closed (Figure 5D). In these states, the Ala636 residues pack closely against the side chains of Leu638 and Thr639 on the adjacent subunit and form a hydrophobic interaction network that most likely contributes to the stability of the closed-channel states (Figure 5D, lower panels). The substitution of alanine with threonine introduces additional bulk and polarity into this network, which is predicted to destabilize the closed-channel configuration of the upper M3. In contrast, in the active, open-channel model, the tips of the M3 helices have moved away from the channel center axis. As a result, the four Ala636 side chains do not interact with any channel residues, facing a direction allowing the extra methyl and hydroxyl groups introduced by the p.Ala636Thr variation (Figure 5D). Therefore, the models suggest that p.Ala636Thr selectively destabilizes the closed-channel conformations observed in the resting and non-desensitized states, thereby shifting receptor equilibrium towards the active state. This analysis is in good agreement with the functional results that show the p.Ala636Thr variant to have enhanced activation and decreased desensitization (Figure 5A).

Ile627 is located down the M3 helix below the channel gate and above the selectivity filter at the tip of the M2 re-entry loop. In the resting and desensitized states, the models show the isoleucine side chain projecting towards the channel center (indicated with a blue arrow in the top panel, Figure 5C) and not forming any interactions with other residues. In these conformations, the substitution

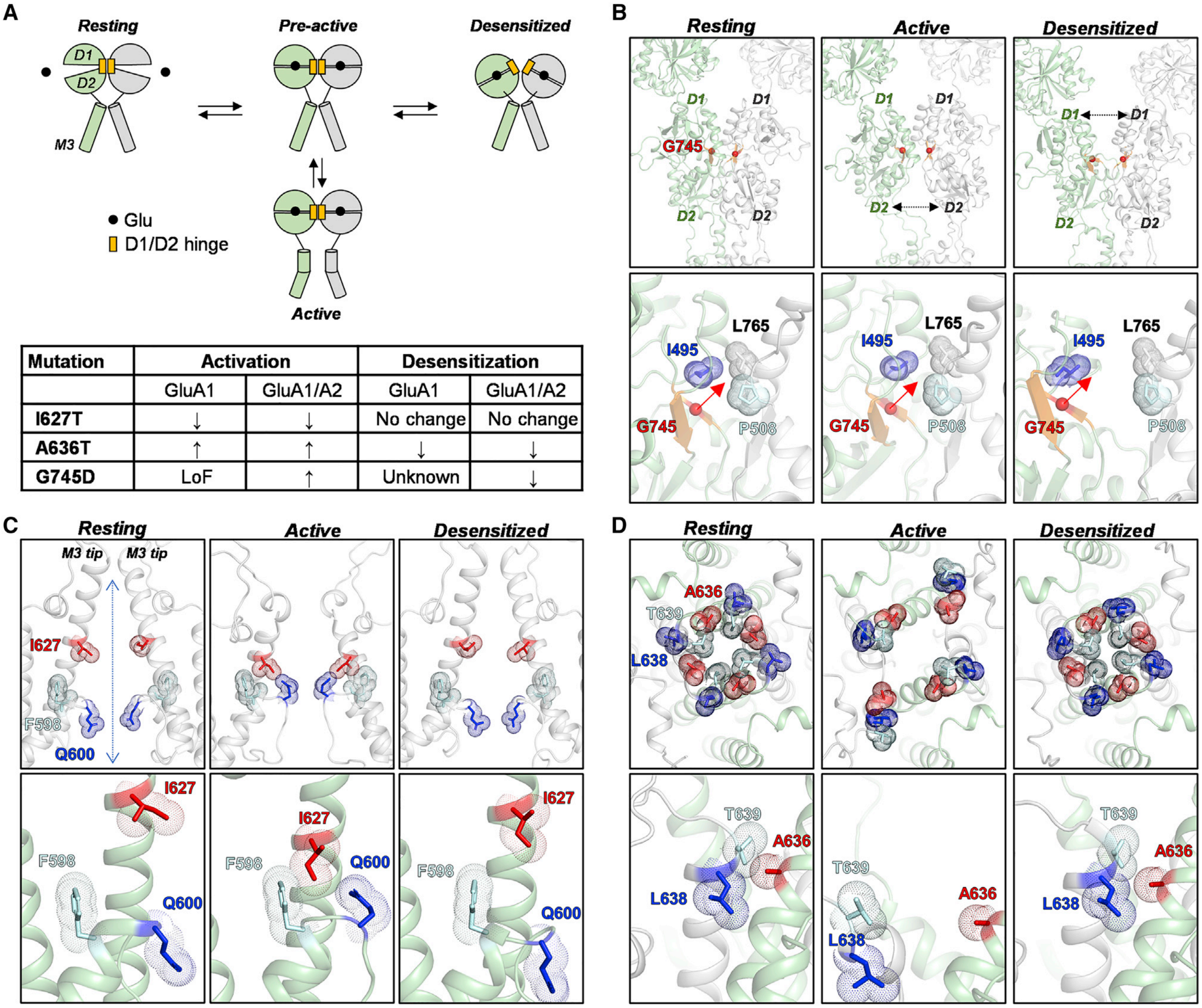

Mutation	Activation		Desensitization	
	GluA1	GluA1/A2	GluA1	GluA1/A2
I627T	↓	↓	No change	No change
A636T	↑	↑	↓	↓
G745D	LoF	↑	Unknown	↓

Figure 5. Structural role of GluA1 residues affected by the *GRIA1* variants

(A) Cartoon illustration of a four-state model for the structural mechanism underlying AMPAR function and summary of the effects of the p.Ile627Thr, p.Ala636Thr, and p.Gly745Asp variations (indicated by I627T, A636T, and G745D, respectively). For simplicity, only two subunits with the ABD and M3 segments (green and gray, respectively) are shown, organized in a dimer complex. In the model, agonist (*black spheres*) binding to the clamshell-shaped ABD promotes the transition from the resting state to the pre-active state where the D1 and D2 subdomains of the ABD adopt a closed conformation around the agonist. From the pre-active state, the receptor can transition into the active state, which involves conformational changes in the upper region of the M3 helices that open the channel, or to a desensitized state, which involves conformational changes around the ABD dimer interface. The D1/D2 hinge region is highlighted in orange. The table shows a qualitative summary of the mutational effects on receptor function with upward and downward arrows indicating increase and decrease, respectively, at homomeric GluA1 and heteromeric GluA1/A2 receptors.

(B) Upper panels show side-on views of the ABD region in a GluA1 subunit dimer (green and gray, respectively) in the resting (left), active (middle), and desensitized (right) states with the Gly745 alpha carbon shown as red spheres. Lower panels show zoomed views of the side chains of Ile495 (blue), Pro508 (cyan), and Leu765 (black) as stick representations with the atomic surface indicated by dots. These residues form a hydrophobic interaction network across the D1/D1 interface in the resting and active states. The p.Gly745Asp variation will project the negatively charged aspartate side chain into this interaction network (indicated by red arrow).

(C) Upper panels show side-on views of the channel region formed by the M3 helices in a GluA1 subunit dimer in the resting (left), active (middle), and desensitized (right) states. The lower panels show zoomed views of the side chains of Ile627 (red), Phe598 (cyan), and Gln600 (blue) as sticks with atomic surfaces indicated by dots. In the active state conformation, but not in the resting and desensitized states, these residues form interactions that might stabilize the open-channel conformation of the M3 helices. The channel center axis is indicated by the blue arrow.

(D) Upper panels show extracellular top views of the channel gate and the side chains of Ala636 (red), Thr639 (cyan), and Leu638 (blue) are shown as sticks with atomic surfaces indicated by dots. The Ala636 side chains contribute to stabilizing the closed-gate conformation by forming hydrophobic interactions with Thr639 and Leu638 in the resting and desensitized conformations (lower panels).

of isoleucine with threonine, which has a similar-sized but polar side chain, can be expected to be tolerated without changing the stability of the closed-channel states. In contrast, in the open-channel conformation of the active state, the Ile627 side chain is close to Phe598 and Gln600 at the tip of the re-entry M2 helix and forms hydrophobic interactions that stabilize the open-channel state. The polar side chain of threonine may disrupt these interactions to destabilize the open-channel conformation and explain the effect of decreased activation of the p.Ile627Thr variation (Figure 5A). In summary, except for p.Arg345Gln, the *GRIA1* variants implicated in NDDs affect residues that are positioned in critical structural domains in the GluA1 subunit protein, and analysis of the potential structural effect of the variants agree well with their observed functional effects to further substantiate their pathogenic status.

Discussion

The *GRIA1-4* genes are emerging as candidate disease-causing genes in NDDs; particularly in forms with severe ID, ASD, and attention-deficit disorder (ADD). Indeed, multiple studies employing *GRIA*-targeted sequencing or WES of individuals with NDD or cohorts have reported variants in all four *GRIA* genes as potential or verified pathogenic.[14,16,65,98–105] These include studies utilizing electrophysiological and biochemical analysis of potential variant effects on the function or expression of recombinant AMPARs containing the subunit variant. Functionally validated variants are so far best described for *GRIA2* and *GRIA3* for which more than 20 missense, insertion/deletion (indel) or stop-gain variants have been reported to change normal receptor function or disrupt or truncate subunit structure; strongly suggesting a linkage between specific *GRIA2* and *GRIA3* variants and NDD phenotypes.[8,65,100,105,106] For example, Salpietro et al.[106] performed functional evaluation of 11 *GRIA2* variants identified in individuals with NDD that included severe ID and found the majority to impact function or expression of GluA2-containing AMPAR subtypes. Similar evaluation of five *GRIA3* missense variants identified in a cohort of 400 unrelated males with X-linked mental retardation (XLMR) found all to drastically alter or destroy the function of GluA3-containing AMPARs.[100] *GRIA4* missense variants have also been associated with NDD phenotypes with severe ID,[98] but functional evaluation has not yet established whether these *GRIA4* variants change the function of GluA4-containing AMPAR subtypes.

In contrast to *GRIA2*, *GRIA3*, and *GRIA4*, the identity of *GRIA1* as an NDD-causing gene has yet to be established, and only three *GRIA1* missense variants so far have been reported in NDD individuals (p.Ile627Thr, p.Ala636Thr, and p.Gly745Asp).[14–16] Interestingly, the p.Ala636Thr variant has been recurrently identified in six unrelated individuals via WES or targeted *GRIA1* sequencing in two large NDD cohorts.[14,16] However, none of these *GRIA1* variants reported in the literature have been functionally evaluated for the potential impact on the expression and function of GluA1-containing AMPARs and, therefore, the pathogenic significance of *GRIA1* in NDDs has remained unclear. Consequently, the diagnostic interpretation and reporting of *GRIA1* variants are at present challenging: *GRIA1* variants are classified as variants of uncertain significance as per the Association for Clinical Genomic Science (ACGS) guidelines for variant classification.[107] Furthermore, *GRIA1* is not part of most commercial or custom-made gene panels used for genetic diagnosis of NDD-affected individuals and is not included in most clinical genetic knowledge bases, such as the widely used UK-based PanelApp web resource, where virtual gene panels related to human disease are curated. Together, these uncertainties regarding the pathophysiological role of *GRIA1* variants have limited diagnosis and potential AMPAR-targeted drug treatment options.

In the present work, a phenotype has been identified in individuals with *GRIA1* variants that includes ID, speech and language delay, poor sleep, abnormal EEG with or without seizures, normal brain imaging, and endocrine abnormalities. Additionally, only missense heterozygous variants have been reported within the current literature, and our index case is the first homozygous nonsense variant in *GRIA1* causing a neurodevelopmental delay phenotype. Functional evaluation of the *GRIA1* variants identified in the individuals was performed with electrophysiological and biochemical analyses, which characterized variant-induced changes in receptor function and expression. Crucially, three of the four missense variants caused significant changes in the function of homomeric GluA1 and heteromeric GluA1/A2 subtypes, whether this was in the current response amplitudes, degree of desensitization, or receptor activation. The most pronounced abnormalities were demonstrated by the p.Gly745Asp variant, which had a minimal current response, and the homozygous nonsense variant (p.Arg377Ter), which demonstrated no current response to Glu. In addition to the lack of current response, shown in the p.Arg377Ter variant, there was no cell surface expression. These findings support the notion that homozygous nonsense variants result in no functioning *GRIA1* gene, leading to increased severity in phenotype, and are internally consistent with the results of mutants in the *Xenopus* model (below). Interestingly, the p.Ala636Thr variant showed an increased current response that was thought to be secondary to increased sensitivity to glutamate and a loss of desensitization. Our findings demonstrate that *GRIA1* contributes to two syndromes with an autosomal recessive and autosomal dominant inheritance pattern and increased severity demonstrated by early-onset seizures and profound speech and language delay in the autosomal recessive index case.

Fundamental insight into the physiological role of GluA1 has come from gene deletion studies in mice where knockout models of the GluA1-encoding gene *Gria1* have been

established and report normal development and life expectancy in *Gria1*$^{-/-}$ animals.[57,108–112] However, behavioral inconsistencies are often reported in studies employing the *Gria1*$^{-/-}$ model, including hyperactivity,[57,109–113] impaired spatial working memory,[54–57] and abnormalities in prepulse inhibition[114] and sleep EEG in keeping with those found in schizophrenia.[115] Here, we extend *GRIA1* gene function analysis to a second model utilizing a CRISPR-based loss-of-function analysis in crispant *X. tropicalis* tadpoles and use it to test explicitly the genotype-phenotype link caused by truncation of *GRIA1*. *Xenopus* have an extensive track record for cost-effective, high-throughput gene function analysis[116–120] and high evolutionary similarity to mammals but broadly lack robust assays to measure higher executive functions. To date, most behavioral studies in *Xenopus* have focused on understanding behavior in the wild, with some reports detailing laboratory schooling,[121,122] swim and search patterns,[123–126] color differentiation,[127–129] seizure induction,[50,130] and learned behaviours.[129,131] In contrast, sophisticated quantitative behavioral analysis has so far been limited in tadpoles. Against this background, the present work demonstrates the successful adaptation of an established method from other vertebrate models with high translational relevance to humans[34] to test the working memory of *gria1* mutant tadpoles. The initial characterization of the nonsense variant with *Xenopus* tadpoles was undertaken by creating homozygous indels within exon 8 and a separate *gria1* knockout model. Our model demonstrates the quantitative behavioral analysis of higher cognitive functions in *Xenopus* tadpoles for the first time, using a test with the potential for future direct comparison between the animal model and the cohort. Homozygous indels created within exon 8 to mimic the homozygous nonsense variant (p.Arg377Ter) functionally support the description of an NDD phenotype by showing working memory deficits without detectable structural changes to the brain. Overall, these findings are in keeping with those reported in the *gria1*$^{-/-}$ mouse, providing a second, cost-effective model organism to investigate further the functional role of GluA1-containing AMPARs in the brain.

In summary, this study establishes *GRIA1* as a human NDD-causing gene that merits being part of the existing collection of *GRIA*-related NDDs.

Supplemental information

Supplemental information can be found online at https://doi.org/10.1016/j.ajhg.2022.05.009.

Acknowledgments

Thank you to all individuals and families. D.B. is generously supported by a National Institute for Health and Care Research research professorship RP-2016-07-011. A.S.K. is supported by the Lundbeck Foundation, the Novo Nordisk Foundation, the Carlsberg Foundation, and the Hørslev Foundation. We thank Zantiks (Cambridge), in particular Dr. Shanelle Kohler, for their continued support for the establishment of measurable behavioral parameters in *Xenopus*. We are grateful to Dr. Colin Sharpe, University of Portsmouth, for his expert feedback on the manuscript. The European *Xenopus* Resource Centre is supported by the Wellcome Trust (101480Z), BBSRC (BB/K019988/1), and MRC (MR/V012177/1). We are also grateful to the University of Portsmouth for funding A.G.'s PhD project.

Declaration of interests

The authors declare no competing interests.

Received: December 16, 2021
Accepted: May 11, 2022
Published: June 7, 2022

Web Resources

Addgene, http://n2t.net/addgene
ClinVar, https://www.ncbi.nlm.nih.gov/clinvar
Decipher, https://decipher.sanger.ac.uk
GeneMatcher, https://genematcher.org
gnomAD, https://gnomad.broadinstitute.org
MTR-viewer, http://biosig.unimelb.edu.au/mtr-viewer/
OMIM, https://www.omim.org
PanelApp, https://panelapp.genomicsengland.co.uk
Xenbase, http://www.xenbase.org/entry/

References

1. Traynelis, S.F., Wollmuth, L.P., McBain, C.J., Menniti, F.S., Vance, K.M., Ogden, K.K., Hansen, K.B., Yuan, H., Myers, S.J., and Dingledine, R. (2010). Glutamate receptor ion channels: structure, regulation, and function. Pharmacol. Rev. *62*, 405–496. https://doi.org/10.1124/pr.109.002451.
2. Bliss, T.V., and Collingridge, G.L. (2013). Expression of NMDA receptor-dependent LTP in the hippocampus: bridging the divide. Mol. Brain *6*, 5. https://doi.org/10.1186/1756-6606-6-5.
3. Huganir, R.L., and Nicoll, R.A. (2013). AMPARs and synaptic plasticity: the last 25 years. Neuron *80*, 704–717. https://doi.org/10.1016/j.neuron.2013.10.025.
4. Malinow, R., and Malenka, R.C. (2002). AMPA receptor trafficking and synaptic plasticity. Annu. Rev. Neurosci. *25*, 103–126. https://doi.org/10.1146/annurev.neuro.25.112701.142758.
5. Hollmann, M., and Heinemann, S. (1994). Cloned glutamate receptors. Annu. Rev. Neurosci. *17*, 31–108. https://doi.org/10.1146/annurev.ne.17.030194.000335.
6. Traynelis, J., Silk, M., Wang, Q., Berkovic, S.F., Liu, L., Ascher, D.B., Balding, D.J., and Petrovski, S. (2017). Optimizing genomic medicine in epilepsy through a gene-customized approach to missense variant interpretation. Genome Res. *27*, 1715–1729. https://doi.org/10.1101/gr.226589.117.
7. Leonard, H., and Wen, X. (2002). The epidemiology of mental retardation: challenges and opportunities in the new millennium. Ment. Retard. Dev. Disabil. Res. Rev. *8*, 117–134. https://doi.org/10.1002/mrdd.10031.
8. Chelly, J., and Mandel, J.L. (2001). Monogenic causes of X-linked mental retardation. Nat. Rev. Genet. *2*, 669–680. https://doi.org/10.1038/35088558.
9. Ropers, H.H. (2010). Genetics of early onset cognitive impairment. Annu. Rev. Genom. Hum. Genet. *11*, 161–187. https://doi.org/10.1146/annurev-genom-082509-141640.

10. Sheridan, E., Wright, J., Small, N., Corry, P.C., Oddie, S., Whibley, C., Petherick, E.S., Malik, T., Pawson, N., McKinney, P.A., and Parslow, R.C. (2013). Risk factors for congenital anomaly in a multiethnic birth cohort: an analysis of the Born in Bradford study. Lancet *382*, 1350–1359. https://doi.org/10.1016/s0140-6736(13)61132-0.
11. Hamdan, F.F., Gauthier, J., Araki, Y., Lin, D.T., Yoshizawa, Y., Higashi, K., Park, A.R., Spiegelman, D., Dobrzeniecka, S., Piton, A., et al. (2011). Excess of de novo deleterious mutations in genes associated with glutamatergic systems in nonsyndromic intellectual disability. Am. J. Hum. Genet. *88*, 306–316. https://doi.org/10.1016/j.ajhg.2011.03.011.
12. Kaufman, L., Ayub, M., and Vincent, J.B. (2010). The genetic basis of non-syndromic intellectual disability: a review. J. Neurodev. Disord. *2*, 182–209. https://doi.org/10.1007/s11689-010-9055-2.
13. Yuan, H., Low, C.M., Moody, O.A., Jenkins, A., and Traynelis, S.F. (2015). Ionotropic GABA and glutamate receptor mutations and human neurologic diseases. Mol. Pharmacol. *88*, 203–217. https://doi.org/10.1124/mol.115.097998.
14. de Ligt, J., Willemsen, M.H., van Bon, B.W.M., Kleefstra, T., Yntema, H.G., Kroes, T., Vulto-van Silfhout, A.T., Koolen, D.A., de Vries, P., Gilissen, C., et al. (2012). Diagnostic exome sequencing in persons with severe intellectual disability. N. Engl. J. Med. *367*, 1921–1929. https://doi.org/10.1056/NEJMoa1206524.
15. Guo, H., Duyzend, M.H., Coe, B.P., Baker, C., Hoekzema, K., Gerdts, J., Turner, T.N., Zody, M.C., Beighley, J.S., Murali, S.C., et al. (2019). Genome sequencing identifies multiple deleterious variants in autism patients with more severe phenotypes. Genet. Med. : official journal of the American College of Medical Genetics *21*, 1611–1620. https://doi.org/10.1038/s41436-018-0380-2.
16. Geisheker, M.R., Heymann, G., Wang, T., Coe, B.P., Turner, T.N., Stessman, H.A.F., Hoekzema, K., Kvarnung, M., Shaw, M., Friend, K., et al. (2017). Hotspots of missense mutation identify neurodevelopmental disorder genes and functional domains. Nat. Neurosci. *20*, 1043–1051. https://doi.org/10.1038/nn.4589.
17. Turner, T.N., Hormozdiari, F., Duyzend, M.H., McClymont, S.A., Hook, P.W., Iossifov, I., Raja, A., Baker, C., Hoekzema, K., Stessman, H.A., et al. (2016). Genome sequencing of autism-affected families reveals disruption of putative noncoding regulatory DNA. Am. J. Hum. Genet. *98*, 58–74. https://doi.org/10.1016/j.ajhg.2015.11.023.
18. Iossifov, I., O'Roak, B.J., Sanders, S.J., Ronemus, M., Krumm, N., Levy, D., Stessman, H.A., Witherspoon, K.T., Vives, L., Patterson, K.E., et al. (2014). The contribution of de novo coding mutations to autism spectrum disorder. Nature *515*, 216–221. https://doi.org/10.1038/nature13908.
19. Koire, A., Katsonis, P., Kim, Y.W., Buchovecky, C., Wilson, S.J., and Lichtarge, O. (2021). A method to delineate de novo missense variants across pathways prioritizes genes linked to autism. Sci. Transl. Med. *13*. https://doi.org/10.1126/scitranslmed.abc1739.
20. Turner, T.N., Wilfert, A.B., Bakken, T.E., Bernier, R.A., Pepper, M.R., Zhang, Z., Torene, R.I., Retterer, K., and Eichler, E.E. (2019). Sex-based analysis of de novo variants in neurodevelopmental disorders. Am. J. Hum. Genet. *105*, 1274–1285. https://doi.org/10.1016/j.ajhg.2019.11.003.
21. Sobreira, N., Schiettecatte, F., Valle, D., and Hamosh, A. (2015). GeneMatcher: a matching tool for connecting investigators with an interest in the same gene. Hum. Mutat. *36*, 928–930. https://doi.org/10.1002/humu.22844.
22. Landrum, M.J., Lee, J.M., Benson, M., Brown, G.R., Chao, C., Chitipiralla, S., Gu, B., Hart, J., Hoffman, D., Jang, W., et al. (2018). ClinVar: improving access to variant interpretations and supporting evidence. Nucleic Acids Res. *46*, D1062–D1067. https://doi.org/10.1093/nar/gkx1153.
23. Rentzsch, P., Witten, D., Cooper, G.M., Shendure, J., and Kircher, M. (2019). CADD: predicting the deleteriousness of variants throughout the human genome. Nucleic Acids Res. *47*, D886–D894. https://doi.org/10.1093/nar/gky1016.
24. Ng, P.C., and Henikoff, S. (2003). SIFT: predicting amino acid changes that affect protein function. Nucleic Acids Res. *31*, 3812–3814. https://doi.org/10.1093/nar/gkg509.
25. Adzhubei, I.A., Schmidt, S., Peshkin, L., Ramensky, V.E., Gerasimova, A., Bork, P., Kondrashov, A.S., and Sunyaev, S.R. (2010). A method and server for predicting damaging missense mutations. Nat. Methods *7*, 248–249. https://doi.org/10.1038/nmeth0410-248.
26. Sargent, M.G., and Mohun, T.J. (2005). Cryopreservation of sperm of Xenopus laevis and Xenopus tropicalis. Genesis *41*, 41–46. https://doi.org/10.1002/gene.20092.
27. Mansour, N., Lahnsteiner, F., and Patzner, R.A. (2009). Optimization of the cryopreservation of African clawed frog (Xenopus laevis) sperm. Theriogenology *72*, 1221–1228. https://doi.org/10.1016/j.theriogenology.2009.07.013.
28. Xenopus online resource | Xenopus resource Centre (EXRC). In. (
29. Karimi, K., Fortriede, J.D., Lotay, V.S., Burns, K.A., Wang, D.Z., Fisher, M.E., Pells, T.J., James-Zorn, C., Wang, Y., Ponferrada, V.G., et al. (2018). Xenbase: a genomic, epigenomic and transcriptomic model organism database. Nucleic Acids Res. *46*, D861–D868. https://doi.org/10.1093/nar/gkx936.
30. Moreno-Mateos, M.A., Vejnar, C.E., Beaudoin, J.D., Fernandez, J.P., Mis, E.K., Khokha, M.K., and Giraldez, A.J. (2015). CRISPRscan: designing highly efficient sgRNAs for CRISPR-Cas9 targeting in vivo. Nat. Methods *12*, 982–988. https://doi.org/10.1038/nmeth.3543.
31. Shen, M.W., Arbab, M., Hsu, J.Y., Worstell, D., Culbertson, S.J., Krabbe, O., Cassa, C.A., Liu, D.R., Gifford, D.K., and Sherwood, R.I. (2018). Predictable and precise template-free CRISPR editing of pathogenic variants. Nature *563*, 646–651. https://doi.org/10.1038/s41586-018-0686-x.
32. Nakayama, T., Blitz, I.L., Fish, M.B., Odeleye, A.O., Manohar, S., Cho, K.W., and Grainger, R.M. (2014). Cas9-based genome editing in Xenopus tropicalis. Methods Enzymol. *546*, 355–375. https://doi.org/10.1016/b978-0-12-801185-0.00017-9.
33. Untergasser, A., Cutcutache, I., Koressaar, T., Ye, J., Faircloth, B.C., Remm, M., and Rozen, S.G. (2012). Primer3–new capabilities and interfaces. Nucleic Acids Res. *40*, e115. https://doi.org/10.1093/nar/gks596.
34. Cleal, M., Fontana, B.D., Ranson, D.C., McBride, S.D., Swinny, J.D., Redhead, E.S., and Parker, M.O. (2021). The Free-movement pattern Y-maze: a cross-species measure of working memory and executive function. Behav. Res. Methods *53*, 536–557. https://doi.org/10.3758/s13428-020-01452-x.
35. Fontana, B.D., Cleal, M., Clay, J.M., and Parker, M.O. (2019). Zebrafish (Danio rerio) behavioral laterality predicts increased short-term avoidance memory but not stress-reactivity responses. Anim Cogn *22*, 1051–1061. https://doi.org/10.1007/s10071-019-01296-9.

36. Fontana, B.D., Cleal, M., Gibbon, A.J., McBride, S.D., and Parker, M.O. (2021). The effects of two stressors on working memory and cognitive flexibility in zebrafish (Danio rerio): the protective role of D1/D5 agonist on stress responses. Neuropharmacology *196*, 108681. https://doi.org/10.1016/j.neuropharm.2021.108681.
37. Faul, F., Erdfelder, E., Lang, A.G., and Buchner, A. (2007). G*Power 3: a flexible statistical power analysis program for the social, behavioral, and biomedical sciences. Behav. Res. Methods *39*, 175–191. https://doi.org/10.3758/bf03193146.
38. Stenum-Berg, C., Musgaard, M., Chavez-Abiega, S., Thisted, C.L., Barrella, L., Biggin, P.C., and Kristensen, A.S. (2019). Mutational analysis and modeling of negative allosteric modulator binding sites in AMPA receptors. Mol. Pharmacol. *96*, 835–850. https://doi.org/10.1124/mol.119.116871.
39. Hayashi, Y., Shi, S.H., Esteban, J.A., Piccini, A., Poncer, J.C., and Malinow, R. (2000). Driving AMPA receptors into synapses by LTP and CaMKII: requirement for GluR1 and PDZ domain interaction. Science *287*, 2262–2267. https://doi.org/10.1126/science.287.5461.2262.
40. Lu, P., Bai, X.C., Ma, D., Xie, T., Yan, C., Sun, L., Yang, G., Zhao, Y., Zhou, R., Scheres, S.H.W., and Shi, Y. (2014). Three-dimensional structure of human gamma-secretase. Nature *512*, 166–170. https://doi.org/10.1038/nature13567.
41. Poulsen, M.H., Lucas, S., Stromgaard, K., and Kristensen, A.S. (2014). Evaluation of PhTX-74 as subtype-selective inhibitor of GluA2-containing AMPA receptors. Mol. Pharmacol. *85*, 261–268. https://doi.org/10.1124/mol.113.089961.
42. Lam, V.M., Beerepoot, P., Angers, S., and Salahpour, A. (2013). A novel assay for measurement of membrane-protein surface expression using a β-lactamase reporter. Traffic *14*, 778–784. https://doi.org/10.1111/tra.12073.
43. Joshi, P.R., Suryanarayanan, A., and Schulte, M.K. (2004). A vertical flow chamber for Xenopus oocyte electrophysiology and automated drug screening. J. Neurosci. Methods *132*, 69–79. https://doi.org/10.1016/j.jneumeth.2003.09.002.
44. Li, J., Zhang, J., Tang, W., Mizu, R.K., Kusumoto, H., Xiang-Wei, W., Xu, Y., Chen, W., Amin, J.B., Hu, C., et al. (2019). De novo GRIN variants in NMDA receptor M2 channel pore-forming loop are associated with neurological diseases. Hum. Mutat. *40*, 2393–2413. https://doi.org/10.1002/humu.23895.
45. Swanger, S.A., Chen, W., Wells, G., Burger, P.B., Tankovic, A., Bhattacharya, S., Strong, K.L., Hu, C., Kusumoto, H., Zhang, J., et al. (2016). Mechanistic insight into NMDA receptor dysregulation by rare variants in the GluN2A and GluN2B agonist binding domains. Am. J. Hum. Genet. *99*, 1261–1280. https://doi.org/10.1016/j.ajhg.2016.10.002.
46. Barbosa, S., Greville-Heygate, S., Bonnet, M., Godwin, A., Fagotto-Kaufmann, C., Kajava, A.V., Laouteouet, D., Mawby, R., Wai, H.A., Dingemans, A.J.M., et al. (2020). Opposite modulation of RAC1 by mutations in TRIO is associated with distinct, domain-specific neurodevelopmental disorders. Am. J. Hum. Genet. *106*, 338–355. https://doi.org/10.1016/j.ajhg.2020.01.018.
47. Macken, W.L., Godwin, A., Wheway, G., Stals, K., Nazlamova, L., Ellard, S., Alfares, A., Aloraini, T., AlSubaie, L., Alfadhel, M., et al. (2021). Biallelic variants in COPB1 cause a novel, severe intellectual disability syndrome with cataracts and variable microcephaly. Genome Med. *13*, 34. https://doi.org/10.1186/s13073-021-00850-w.
48. Deniz, E., Mis, E.K., Lane, M., and Khokha, M.K. (2018). CRISPR/Cas9 F0 screening of congenital heart disease genes in Xenopus tropicalis. Methods Mol. Biol. *1865*, 163–174. https://doi.org/10.1007/978-1-4939-8784-9_12.
49. Sega, A.G., Mis, E.K., Lindstrom, K., Mercimek-Andrews, S., Ji, W., Cho, M.T., Juusola, J., Konstantino, M., Jeffries, L., Khokha, M.K., and Lakhani, S.A. (2019). De novo pathogenic variants in neuronal differentiation factor 2 (NEUROD2) cause a form of early infantile epileptic encephalopathy. J. Med. Genet. *56*, 113–122. https://doi.org/10.1136/jmedgenet-2018-105322.
50. Hewapathirane, D.S., Dunfield, D., Yen, W., Chen, S., and Haas, K. (2008). In vivo imaging of seizure activity in a novel developmental seizure model. Exp. Neurol. *211*, 480–488. https://doi.org/10.1016/j.expneurol.2008.02.012.
51. Pratt, K.G., and Khakhalin, A.S. (2013). Modeling human neurodevelopmental disorders in the Xenopus tadpole: from mechanisms to therapeutic targets. Dis Model Mech *6*, 1057–1065. https://doi.org/10.1242/dmm.012138.
52. Shapiro, M.L., and O'Connor, C. (1992). N-methyl-D-aspartate receptor antagonist MK-801 and spatial memory representation: working memory is impaired in an unfamiliar but not in a familiar environment. Behav. Neurosci. *106*, 604–612. https://doi.org/10.1037/0735-7044.106.4.604.
53. Brosnan-Watters, G., Wozniak, D.F., Nardi, A., and Olney, J.W. (1996). Acute behavioral effects of MK-801 in the mouse. Pharmacol. Biochem. Behav. *53*, 701–711. https://doi.org/10.1016/0091-3057(95)02073-x.
54. Sanderson, D.J., Good, M.A., Skelton, K., Sprengel, R., Seeburg, P.H., Rawlins, J.N.P., and Bannerman, D.M. (2009). Enhanced long-term and impaired short-term spatial memory in GluA1 AMPA receptor subunit knockout mice: evidence for a dual-process memory model. Learn. Mem. *16*, 379–386. https://doi.org/10.1101/lm.1339109.
55. Reisel, D., Bannerman, D.M., Schmitt, W.B., Deacon, R.M.J., Flint, J., Borchardt, T., Seeburg, P.H., and Rawlins, J.N.P. (2002). Spatial memory dissociations in mice lacking GluR1. Nat. Neurosci. *5*, 868–873. https://doi.org/10.1038/nn910.
56. Schmitt, W.B., Deacon, R.M.J., Seeburg, P.H., Rawlins, J.N.P., and Bannerman, D.M. (2003). A within-subjects, within-task demonstration of intact spatial reference memory and impaired spatial working memory in glutamate receptor-A-deficient mice. J. Neurosci. *23*, 3953–3959. https://doi.org/10.1523/jneurosci.23-09-03953.2003.
57. Zamanillo, D., Sprengel, R., Hvalby, O., Jensen, V., Burnashev, N., Rozov, A., Kaiser, K.M.M., Koster, H.J., Borchardt, T., Worley, P., et al. (1999). Importance of AMPA receptors for hippocampal synaptic plasticity but not for spatial learning. Science *284*, 1805–1811. https://doi.org/10.1126/science.284.5421.1805.
58. Sanderson, D.J., Sprengel, R., Seeburg, P.H., and Bannerman, D.M. (2011). Deletion of the GluA1 AMPA receptor subunit alters the expression of short-term memory. Learn. Mem. *18*, 128–131. https://doi.org/10.1101/lm.2014911.
59. Greger, I.H., Watson, J.F., and Cull-Candy, S.G. (2017). Structural and functional architecture of AMPA-type glutamate receptors and their auxiliary proteins. Neuron *94*, 713–730. https://doi.org/10.1016/j.neuron.2017.04.009.
60. Herguedas, B., Garcia-Nafria, J., Cais, O., Fernandez-Leiro, R., Krieger, J., Ho, H., and Greger, I.H. (2016). Structure and organization of heteromeric AMPA-type glutamate receptors. Science *352*, aad3873. https://doi.org/10.1126/science.aad3873.

61. Herguedas, B., Krieger, J., and Greger, I.H. (2013). Receptor heteromeric assembly—how it works and why it matters. Progress in molecular biology and translational science *117*, 361–386. https://doi.org/10.1016/b978-0-12-386931-9.00013-1.
62. Sommer, B., Keinanen, K., Verdoorn, T.A., Wisden, W., Burnashev, N., Herb, A., Kohler, M., Takagi, T., Sakmann, B., and Seeburg, P.H. (1990). Flip and flop: a cell-specific functional switch in glutamate-operated channels of the CNS. Science *249*, 1580–1585. https://doi.org/10.1126/science.1699275.
63. Plested, A.R., Wildman, S.S., Lieb, W.R., and Franks, N.P. (2004). Determinants of the sensitivity of AMPA receptors to xenon. Anesthesiology *100*, 347–358. https://doi.org/10.1097/00000542-200402000-00025.
64. Robert, A., Irizarry, S.N., Hughes, T.E., and Howe, J.R. (2001). Subunit interactions and AMPA receptor desensitization. J. Neurosci. *21*, 5574–5586. https://doi.org/10.1523/jneurosci.21-15-05574.2001.
65. Davies, B., Brown, L.A., Cais, O., Watson, J., Clayton, A.J., Chang, V.T., Biggs, D., Preece, C., Hernandez-Pliego, P., Krohn, J., et al.; The WGS500 Consortium (2017). A point mutation in the ion conduction pore of AMPA receptor GRIA3 causes dramatically perturbed sleep patterns as well as intellectual disability. Hum. Mol. Genet. *26*, 3869–3882. https://doi.org/10.1093/hmg/ddx270.
66. Schmid, S.M., Korber, C., Herrmann, S., Werner, M., and Hollmann, M. (2007). A domain linking the AMPA receptor agonist binding site to the ion pore controls gating and causes lurcher properties when mutated. J. Neurosci. *27*, 12230–12241. https://doi.org/10.1523/jneurosci.3175-07.2007.
67. Klein, R.M., and Howe, J.R. (2004). Effects of the lurcher mutation on GluR1 desensitization and activation kinetics. J. Neurosci. *24*, 4941–4951. https://doi.org/10.1523/jneurosci.0660-04.2004.
68. Taverna, F., Xiong, Z.G., Brandes, L., Roder, J.C., Salter, M.W., and MacDonald, J.F. (2000). The Lurcher mutation of an alpha-amino-3-hydroxy-5-methyl- 4-isoxazolepropionic acid receptor subunit enhances potency of glutamate and converts an antagonist to an agonist. J. Biol. Chem. *275*, 8475–8479. https://doi.org/10.1074/jbc.275.12.8475.
69. Kohda, K., Wang, Y., and Yuzaki, M. (2000). Mutation of a glutamate receptor motif reveals its role in gating and δ2 receptor channel properties. Nat. Neurosci. *3*, 315–322. https://doi.org/10.1038/73877.
70. Twomey, E.C., and Sobolevsky, A.I. (2018). Structural mechanisms of gating in ionotropic glutamate receptors. Biochemistry *57*, 267–276. https://doi.org/10.1021/acs.biochem.7b00891.
71. Twomey, E.C., Yelshanskaya, M.V., Grassucci, R.A., Frank, J., and Sobolevsky, A.I. (2017). Channel opening and gating mechanism in AMPA-subtype glutamate receptors. Nature *549*, 60–65. https://doi.org/10.1038/nature23479.
72. Chen, S., Zhao, Y., Wang, Y., Shekhar, M., Tajkhorshid, E., and Gouaux, E. (2017). Activation and desensitization mechanism of AMPA receptor-TARP complex by cryo-EM. Cell *170*, 1234–1246.e14. https://doi.org/10.1016/j.cell.2017.07.045.
73. Phillips, R.J.S. (1960). 'Lurcher', a new gene in linkage group XI of the house mouse. J. Genet. *57*, 35–42. https://doi.org/10.1007/bf02985337.
74. Zuo, J., De Jager, P.L., Takahashi, K.A., Jiang, W., Linden, D.J., and Heintz, N. (1997). Neurodegeneration in Lurcher mice caused by mutation in δ2 glutamate receptor gene. Nature *388*, 769–773. https://doi.org/10.1038/42009.
75. Ge, Y., and Wang, Y.T. (2021). GluA1-homomeric AMPA receptor in synaptic plasticity and neurological diseases. Neuropharmacology *197*, 108708. https://doi.org/10.1016/j.neuropharm.2021.108708.
76. Schwenk, J., Baehrens, D., Haupt, A., Bildl, W., Boudkkazi, S., Roeper, J., Fakler, B., and Schulte, U. (2014). Regional diversity and developmental dynamics of the AMPA-receptor proteome in the mammalian brain. Neuron *84*, 41–54. https://doi.org/10.1016/j.neuron.2014.08.044.
77. Schwenk, J., Harmel, N., Brechet, A., Zolles, G., Berkefeld, H., Muller, C.S., Bildl, W., Baehrens, D., Huber, B., Kulik, A., et al. (2012). High-resolution proteomics unravel architecture and molecular diversity of native AMPA receptor complexes. Neuron *74*, 621–633. https://doi.org/10.1016/j.neuron.2012.03.034.
78. Jackson, A.C., and Nicoll, R.A. (2011). The expanding social network of ionotropic glutamate receptors: TARPs and other transmembrane auxiliary subunits. Neuron *70*, 178–199. https://doi.org/10.1016/j.neuron.2011.04.007.
79. Kristensen, A.S., Jenkins, M.A., Banke, T.G., Schousboe, A., Makino, Y., Johnson, R.C., Huganir, R., and Traynelis, S.F. (2011). Mechanism of Ca2+/calmodulin-dependent kinase II regulation of AMPA receptor gating. Nat. Neurosci. *14*, 727–735. https://doi.org/10.1038/nn.2804.
80. Greger, I.H., Khatri, L., and Ziff, E.B. (2002). RNA editing at arg607 controls AMPA receptor exit from the endoplasmic reticulum. Neuron *34*, 759–772. https://doi.org/10.1016/s0896-6273(02)00693-1.
81. Swanson, G.T., Kamboj, S.K., and Cull-Candy, S.G. (1997). Single-channel properties of recombinant AMPA receptors depend on RNA editing, splice variation, and subunit composition. J. Neurosci. *17*, 58–69. https://doi.org/10.1523/jneurosci.17-01-00058.1997.
82. Tomita, S., Adesnik, H., Sekiguchi, M., Zhang, W., Wada, K., Howe, J.R., Nicoll, R.A., and Bredt, D.S. (2005). Stargazin modulates AMPA receptor gating and trafficking by distinct domains. Nature *435*, 1052–1058. https://doi.org/10.1038/nature03624.
83. Korber, C., Werner, M., Kott, S., Ma, Z.L., and Hollmann, M. (2007). The transmembrane AMPA receptor regulatory protein 4 is a more effective modulator of AMPA receptor function than stargazin (2). J. Neurosci. *27*, 8442–8447. https://doi.org/10.1523/jneurosci.0424-07.2007.
84. Kott, S., Sager, C., Tapken, D., Werner, M., and Hollmann, M. (2009). Comparative analysis of the pharmacology of GluR1 in complex with transmembrane AMPA receptor regulatory proteins γ2, γ3, γ4, and γ8. Neuroscience *158*, 78–88. https://doi.org/10.1016/j.neuroscience.2007.12.047.
85. Kott, S., Werner, M., Korber, C., and Hollmann, M. (2007). Electrophysiological properties of AMPA receptors are differentially modulated depending on the associated member of the TARP family. J. Neurosci. *27*, 3780–3789. https://doi.org/10.1523/jneurosci.4185-06.2007.
86. Sager, C., Tapken, D., Kott, S., and Hollmann, M. (2009). Functional modulation of AMPA receptors by transmembrane AMPA receptor regulatory proteins. Neuroscience *158*, 45–54. https://doi.org/10.1016/j.neuroscience.2007.12.046.
87. Priel, A., Kolleker, A., Ayalon, G., Gillor, M., Osten, P., and Stern-Bach, Y. (2005). Stargazin reduces desensitization and slows deactivation of the AMPA-type glutamate receptors. J. Neurosci. *25*, 2682–2686. https://doi.org/10.1523/jneurosci.4834-04.2005.
88. Soto, D., Coombs, I.D., Gratacos-Batlle, E., Farrant, M., and Cull-Candy, S.G. (2014). Molecular mechanisms contributing to

TARP regulation of channel conductance and polyamine block of calcium-permeable AMPA receptors. J. Neurosci. *34*, 11673–11683. https://doi.org/10.1523/jneurosci.0383-14.2014.

89. Shelley, C., Farrant, M., and Cull-Candy, S.G. (2012). TARP-associated AMPA receptors display an increased maximum channel conductance and multiple kinetically distinct open states. J Physiol *590*, 5723–5738. https://doi.org/10.1113/jphysiol.2012.238006.
90. Kamalova, A., and Nakagawa, T. (2021). AMPA receptor structure and auxiliary subunits. J Physiol *599*, 453–469. https://doi.org/10.1113/jp278701.
91. Twomey, E.C., Yelshanskaya, M.V., Grassucci, R.A., Frank, J., and Sobolevsky, A.I. (2017). Structural bases of desensitization in AMPA receptor-auxiliary subunit complexes. Neuron *94*, 569–580.e5. https://doi.org/10.1016/j.neuron.2017.04.025.
92. Chen, S., and Gouaux, E. (2019). Structure and mechanism of AMPA receptor - auxiliary protein complexes. Curr. Opin. Struct. Biol. *54*, 104–111. https://doi.org/10.1016/j.sbi.2019.01.011.
93. Durr, K.L., Chen, L., Stein, R.A., De Zorzi, R., Folea, I.M., Walz, T., McHaourab, H.S., and Gouaux, E. (2014). Structure and dynamics of AMPA receptor GluA2 in resting, pre-open, and desensitized states. Cell *158*, 778–792. https://doi.org/10.1016/j.cell.2014.07.023.
94. Sun, Y., Olson, R., Horning, M., Armstrong, N., Mayer, M., and Gouaux, E. (2002). Mechanism of glutamate receptor desensitization. Nature *417*, 245–253. https://doi.org/10.1038/417245a.
95. Armstrong, N., and Gouaux, E. (2000). Mechanisms for activation and antagonism of an AMPA-sensitive glutamate receptor. Neuron *28*, 165–181. https://doi.org/10.1016/s0896-6273(00)00094-5.
96. Mayer, M.L., Olson, R., and Gouaux, E. (2001). Mechanisms for ligand binding to GluR0 ion channels: crystal structures of the glutamate and serine complexes and a closed apo state. J. Mol. Biol. *311*, 815–836. https://doi.org/10.1006/jmbi.2001.4884.
97. Horning, M.S., and Mayer, M.L. (2004). Regulation of AMPA receptor gating by ligand binding core dimers. Neuron *41*, 379–388. https://doi.org/10.1016/s0896-6273(04)00018-2.
98. Martin, S., Chamberlin, A., Shinde, D.N., Hempel, M., Strom, T.M., Schreiber, A., Johannsen, J., Ousager, L.B., Larsen, M.J., Hansen, L.K., et al. (2017). De novo variants in GRIA4 lead to intellectual disability with or without seizures and gait abnormalities. Am. J. Hum. Genet. *101*, 1013–1020. https://doi.org/10.1016/j.ajhg.2017.11.004.
99. Cherot, E., Keren, B., Dubourg, C., Carre, W., Fradin, M., Lavillaureix, A., Afenjar, A., Burglen, L., Whalen, S., Charles, P., et al. (2018). Using medical exome sequencing to identify the causes of neurodevelopmental disorders: experience of 2 clinical units and 216 patients. Clin. Genet. *93*, 567–576. https://doi.org/10.1111/cge.13102.
100. Wu, Y., Arai, A.C., Rumbaugh, G., Srivastava, A.K., Turner, G., Hayashi, T., Suzuki, E., Jiang, Y., Zhang, L., Rodriguez, J., et al. (2007). Mutations in ionotropic AMPA receptor 3 alter channel properties and are associated with moderate cognitive impairment in humans. Proc. Natl. Acad. Sci. U. S. A. *104*, 18163–18168. https://doi.org/10.1073/pnas.0708699104.
101. Philips, A.K., Siren, A., Avela, K., Somer, M., Peippo, M., Ahvenainen, M., Doagu, F., Arvio, M., Kaariainen, H., Van Esch, H., et al. (2014). X-exome sequencing in Finnish families with intellectual disability–four novel mutations and two novel syndromic phenotypes. Orphanet J. Rare Dis. *9*, 49. https://doi.org/10.1186/1750-1172-9-49.
102. Allen, N.M., Conroy, J., Shahwan, A., Lynch, B., Correa, R.G., Pena, S.D.J., McCreary, D., Magalhaes, T.R., Ennis, S., Lynch, S.A., and King, M.D. (2016). Unexplained early onset epileptic encephalopathy: exome screening and phenotype expansion. Epilepsia *57*. e12-17. https://doi.org/10.1111/epi.13250.
103. Alkelai, A., Shohat, S., Greenbaum, L., Schechter, T., Draiman, B., Chitrit-Raveh, E., Rienstein, S., Dagaonkar, N., Hughes, D., Aggarwal, V.S., et al. (2021). Expansion of the GRIA2 phenotypic representation: a novel de novo loss of function mutation in a case with childhood onset schizophrenia. J. Hum. Genet. *66*, 339–343. https://doi.org/10.1038/s10038-020-00846-1.
104. Trivisano, M., Santarone, M.E., Micalizzi, A., Ferretti, A., Dentici, M.L., Novelli, A., Vigevano, F., and Specchio, N. (2020). GRIA3 missense mutation is cause of an x-linked developmental and epileptic encephalopathy. Seizure *82*, 1–6. https://doi.org/10.1016/j.seizure.2020.08.032.
105. Sun, J.H., Chen, J., Ayala Valenzuela, F.E., Brown, C., Masser-Frye, D., Jones, M., Romero, L.P., Rinaldi, B., Li, W.L., Li, Q.Q., et al. (2021). X-linked neonatal-onset epileptic encephalopathy associated with a gain-of-function variant p.R660T in GRIA3. PLoS Genet. *17*, e1009608. https://doi.org/10.1371/journal.pgen.1009608.
106. Salpietro, V., Dixon, C.L., Guo, H., Bello, O.D., Vandrovcova, J., Efthymiou, S., Maroofian, R., Heimer, G., Burglen, L., Valence, S., et al. (2019). AMPA receptor GluA2 subunit defects are a cause of neurodevelopmental disorders. Nat. Commun. *10*, 3094. https://doi.org/10.1038/s41467-019-10910-w.
107. Richards, S., Aziz, N., Bale, S., Bick, D., Das, S., Gastier-Foster, J., Grody, W.W., Hegde, M., Lyon, E., Spector, E., et al. (2015). Standards and guidelines for the interpretation of sequence variants: a joint consensus recommendation of the American college of medical genetics and genomics and the association for molecular pathology. Genet. Med. : official journal of the American College of Medical Genetics *17*, 405–424. https://doi.org/10.1038/gim.2015.30.
108. Procaccini, C., Aitta-aho, T., Jaako-Movits, K., Zharkovsky, A., Panhelainen, A., Sprengel, R., Linden, A.M., and Korpi, E.R. (2011). Excessive novelty-induced c-Fos expression and altered neurogenesis in the hippocampus of GluA1 knockout mice. Eur. J. Neurosci. *33*, 161–174. https://doi.org/10.1111/j.1460-9568.2010.07485.x.
109. Vekovischeva, O.Y., Zamanillo, D., Echenko, O., Seppala, T., Uusi-Oukari, M., Honkanen, A., Seeburg, P.H., Sprengel, R., and Korpi, E.R. (2001). Morphine-induced dependence and sensitization are altered in mice deficient in AMPA-type glutamate receptor-A subunits. J. Neurosci. *21*, 4451–4459. https://doi.org/10.1523/jneurosci.21-12-04451.2001.
110. Chourbaji, S., Vogt, M.A., Fumagalli, F., Sohr, R., Frasca, A., Brandwein, C., Hortnagl, H., Riva, M.A., Sprengel, R., and Gass, P. (2008). AMPA receptor subunit 1 (GluR-A) knockout mice model the glutamate hypothesis of depression. FASEB J *22*, 3129–3134. https://doi.org/10.1096/fj.08-106450.
111. Fitzgerald, P.J., Barkus, C., Feyder, M., Wiedholz, L.M., Chen, Y.C., Karlsson, R.M., Machado-Vieira, R., Graybeal, C., Sharp, T., Zarate, C., et al. (2010). Does gene deletion of AMPA GluA1 phenocopy features of schizoaffective disorder? Neurobiol. Dis. *40*, 608–621. https://doi.org/10.1016/j.nbd.2010.08.005.

112. Barkus, C., Feyder, M., Graybeal, C., Wright, T., Wiedholz, L., Izquierdo, A., Kiselycznyk, C., Schmitt, W., Sanderson, D.J., Rawlins, J.N.P., et al. (2012). Do GluA1 knockout mice exhibit behavioral abnormalities relevant to the negative or cognitive symptoms of schizophrenia and schizoaffective disorder? Neuropharmacology *62*, 1263–1272. https://doi.org/10.1016/j.neuropharm.2011.06.005.
113. Bannerman, D.M., Deacon, R.M.J., Brady, S., Bruce, A., Sprengel, R., Seeburg, P.H., and Rawlins, J.N.P. (2004). A comparison of GluR-A-deficient and wild-type mice on a test battery assessing sensorimotor, affective, and cognitive behaviors. Behav. Neurosci. *118*, 643–647. https://doi.org/10.1037/0735-7044.118.3.643.
114. Wiedholz, L.M., Owens, W.A., Horton, R.E., Feyder, M., Karlsson, R.M., Hefner, K., Sprengel, R., Celikel, T., Daws, L.C., and Holmes, A. (2008). Mice lacking the AMPA GluR1 receptor exhibit striatal hyperdopaminergia and 'schizophrenia-related' behaviors. Mol. Psychiatr. *13*, 631–640. https://doi.org/10.1038/sj.mp.4002056.
115. Ang, G., McKillop, L.E., Purple, R., Blanco-Duque, C., Peirson, S.N., Foster, R.G., Harrison, P.J., Sprengel, R., Davies, K.E., Oliver, P.L., et al. (2018). Absent sleep EEG spindle activity in GluA1 (Gria1) knockout mice: relevance to neuropsychiatric disorders. Transl. Psychiatry *8*, 154. https://doi.org/10.1038/s41398-018-0199-2.
116. DeLay, B.D., Corkins, M.E., Hanania, H.L., Salanga, M., Deng, J.M., Sudou, N., Taira, M., Horb, M.E., and Miller, R.K. (2018). Tissue-specific gene inactivation in Xenopus laevis: knockout of lhx1 in the kidney with CRISPR/Cas9. Genetics *208*, 673–686. https://doi.org/10.1534/genetics.117.300468.
117. Hwang, W.Y., Marquez, J., and Khokha, M.K. (2019). Xenopus: driving the discovery of novel genes in patient disease and their underlying pathological mechanisms relevant for organogenesis. Front. Physiol. *10*, 953. https://doi.org/10.3389/fphys.2019.00953.
118. Kariminejad, A., Szenker-Ravi, E., Lekszas, C., Tajsharghi, H., Moslemi, A.R., Naert, T., Tran, H.T., Ahangari, F., Rajaei, M., Nasseri, M., et al. (2019). Homozygous null TBX4 mutations lead to posterior amelia with pelvic and pulmonary hypoplasia. Am. J. Hum. Genet. *105*, 1294–1301. https://doi.org/10.1016/j.ajhg.2019.10.013.
119. Naert, T., Colpaert, R., Van Nieuwenhuysen, T., Dimitrakopoulou, D., Leoen, J., Haustraete, J., Boel, A., Steyaert, W., Lepez, T., Deforce, D., et al. (2016). CRISPR/Cas9 mediated knockout of rb1 and rbl1 leads to rapid and penetrant retinoblastoma development in Xenopus tropicalis. Sci. Rep. *6*, 35264. https://doi.org/10.1038/srep35264.
120. Naert, T., and Vleminckx, K. (2018). CRISPR/Cas9 disease models in zebrafish and Xenopus: the genetic renaissance of fish and frogs. Drug Discov. Today Technol. *28*, 41–52. https://doi.org/10.1016/j.ddtec.2018.07.001.
121. Katz, L.C., Potel, M.J., and Wassersug, R.J. (1981). Structure and mechanisms of schooling intadpoles of the clawed frog, Xenopus laevis. Anim. Behav. *29*, 20–33. https://doi.org/10.1016/s0003-3472(81)80148-0.
122. Lopez, V., 3rd, Khakhalin, A.S., and Aizenman, C. (2021). Schooling in Xenopus laevis tadpoles as a way to assess their neural development. Cold Spring Harb. Protoc. https://doi.org/10.1101/pdb.prot106906.
123. Truszkowski, T.L.S., James, E.J., Hasan, M., Wishard, T.J., Liu, Z., Pratt, K.G., Cline, H.T., and Aizenman, C.D. (2016). Fragile X mental retardation protein knockdown in the developing Xenopus tadpole optic tectum results in enhanced feedforward inhibition and behavioral deficits. Neural Dev. *11*, 14. https://doi.org/10.1186/s13064-016-0069-7.
124. Roberts, A., Hill, N.A., and Hicks, R. (2000). Simple mechanisms organise orientation of escape swimming in embryos and hatchling tadpoles of Xenopus laevis. J. Exp. Biol. *203*, 1869–1885. https://doi.org/10.1242/jeb.203.12.1869.
125. Hanzi, S., and Straka, H. (2018). Wall following in Xenopus laevis is barrier-driven. J Comp Physiol A Neuroethol Sens Neural Behav Physiol *204*, 183–195. https://doi.org/10.1007/s00359-017-1227-z.
126. Videlier, M., Cornette, R., Bonneaud, C., and Herrel, A. (2015). Sexual differences in exploration behavior in Xenopus tropicalis? J. Exp. Biol. *218*, 1733–1739. https://doi.org/10.1242/jeb.120618.
127. Moriya, T., Kito, K., Miyashita, Y., and Asami, K. (1996). Preference for background color of the Xenopus laevis tadpole. J. Exp. Zool. *276*, 335–344. https://doi.org/10.1002/(sici)1097-010x(19961201)276:5<335::aid-jez4>3.0.co;2-p.
128. Viczian, A.S., and Zuber, M.E. (2014). A simple behavioral assay for testing visual function in Xenopus laevis. J Vis Exp. https://doi.org/10.3791/51726.
129. Blackiston, D.J., and Levin, M. (2012). Aversive training methods in Xenopus laevis: general principles. Cold Spring Harb. Protoc. *2012*. https://doi.org/10.1101/pdb.top068338.
130. Bell, M.R., Belarde, J.A., Johnson, H.F., and Aizenman, C.D. (2011). A neuroprotective role for polyamines in a Xenopus tadpole model of epilepsy. Nat. Neurosci. *14*, 505–512. https://doi.org/10.1038/nn.2777.
131. Blackiston, D.J., and Levin, M. (2013). Ectopic eyes outside the head in Xenopus tadpoles provide sensory data for light-mediated learning. J. Exp. Biol. *216*, 1031–1040. https://doi.org/10.1242/jeb.074963.

The American Journal of Human Genetics, Volume *109*

Supplemental information

Identification and functional evaluation of *GRIA1* missense and truncation variants in individuals with ID: An emerging neurodevelopmental syndrome

Vardha Ismail, Linda G. Zachariassen, Annie Godwin, Mane Sahakian, Sian Ellard, Karen L. Stals, Emma Baple, Kate Tatton Brown, Nicola Foulds, Gabrielle Wheway, Matthew O. Parker, Signe M. Lyngby, Miriam G. Pedersen, Julie Desir, Allan Bayat, Maria Musgaard, Matthew Guille, Anders S. Kristensen, and Diana Baralle

Supporting Information

Supplemental Note: Case Reports

Individual 1 (p.Arg377Ter) is a 10-year-old female born to consanguineous parents. She is diagnosed with severe ID and delayed motor development. Although she walked at 14 months of life, her fine motor skills are delayed compared to peers, and she remains non-verbal. In addition, she displays self-injurious behavior and sleeping difficulties. She experienced her first epileptic seizure at six months of life and had recurrent seizures despite being treated with a wide range of anti-seizure medications. She is currently on lamotrigine and ethosuximide. An electroencephalogram showed diffuse background slowing with frequent bursts of spikes and spike-wave discharges across the posterior regions. We were unable to obtain further data in order to classify the seizure or epilepsy types. She also has precocious puberty, feeding difficulties requiring a gastrostomy, constipation, and an intermittent divergent squint.

Individual 2 (p.Ala636Thr) is a 6-year-old female. She is diagnosed with severe ID. She displayed an early developmental delay in gross motor skills. She sat unsupported at 1 year old and began walking at 2 years old. She is non-verbal at the present age. She is reported to have unspecified behavioral issues. No additional medical issues were reported aside from recurrent airway infections. She has not experienced epileptic seizures so far. The Individual was identified via the GeneMatcher platform[1] and is described in a previously reported study of 100 Individuals with severe ID (classed as an IQ below 50). As described in the original report, Individual and unaffected parents were subjected to diagnostic trio-WES analysis[2], which yielded the *GRIA1* missense variant, p.Ala636Thr, as a primary candidate (Table 1).

Individual 3 (p.Ala636Thr) is a 12-year-old male diagnosed with moderate ID. He displayed normal motor development. He sat unsupported at 6 months, walked at 18 months, and can currently run and ride a bike. He is non-verbal at the present age. He has autistic features and is hyperactive. He has poor sleep requiring melatonin and is mildly dysmorphic with telecanthus and full lips. He has not experienced epileptic seizures so far. The Individual was identified from the 100,000 Genomes Project (http://www.genomicsengland.co.uk) following WGS analysis that yielded the *GRIA1* missense variant, p.Ala636Thr, as the primary pathogenic variant candidate (Table 1).

Individual 4 (p.Ala636Thr) is a 26-year-old female with moderate ID. She has some gross motor delay and behavioral problems, including ADHD. Although she used her first word at 1 year of life and spoke in sentences at 2 years of life, she communicates using short and simple sentences and has dysarthria. Additionally, she had astigmatism requiring glasses at 11 months of life. This Individual was identified via the GeneMatcher platform[1]. A clinical exome of approximately 4000 genes was completed, which yielded the *GRIA1* missense variant p.Ala636Thr (Table 1).

Individual 5 (p.Ile627Thr) is a male of unknown age diagnosed with unclassified ID. Only limited access to clinical information was obtained for Individual 5, and further cognitive or motor impairment could not be reported. Other medical issues were reported to be migrainous headaches and a bicuspid aortic valve. Dysmorphic features described were full lips and a high arched palate. This Individual was identified from a study where targeted sequencing was completed for 20 recurrent sites of missense variants in 17,688 Individuals with NDD[3]. As described in the original report, single-molecule molecular inversion probes (smMIP) were designed to capture the regions of interest and the *GRIA1* p.Ile627Thr missense variant was identified (Table 1).

Individual 6 (p.Gly745Asp) is a 20-year-old female diagnosed with moderate-severe ID. She started walking at 13 months of life, but her fine motor skills are delayed compared to peers, and she was found to be hypermobile. She has a speech impairment, with regression from the age of 6 years old. She currently communicates using simple verbal language. She is diagnosed with ASD, bipolar disorder, and a sleeping disorder in addition to polycystic ovarian syndrome and hypothyroidism. The Individual was identified from a previously reported WES study of 213 Individuals with autism spectrum disorders[4] and via the GeneMatcher platform. As detailed in the original report, sequencing was performed on the Illumina HiSeq X Ten platform, and the *GRIA1* missense variant p.Gly745Asp was identified. This variant is also deposited in the ClinVar database (National Center for Biotechnology Information. ClinVar; [VCV000585059.2], https://www.ncbi.nlm.nih.gov/clinvar/variation/VCV000585059.2)[5].

Individual 7 (R345Q) is of unknown sex and age and was identified in the ClinVar database[5] (https://www.ncbi.nlm.nih.gov/clinvar/variation/VCV000983361.1) carrying the *GRIA1* missense variant

(p.Arg345Gln). Unfortunately, aside from being diagnosed with ID, there was limited clinical information available for this Individual at the time of publication.

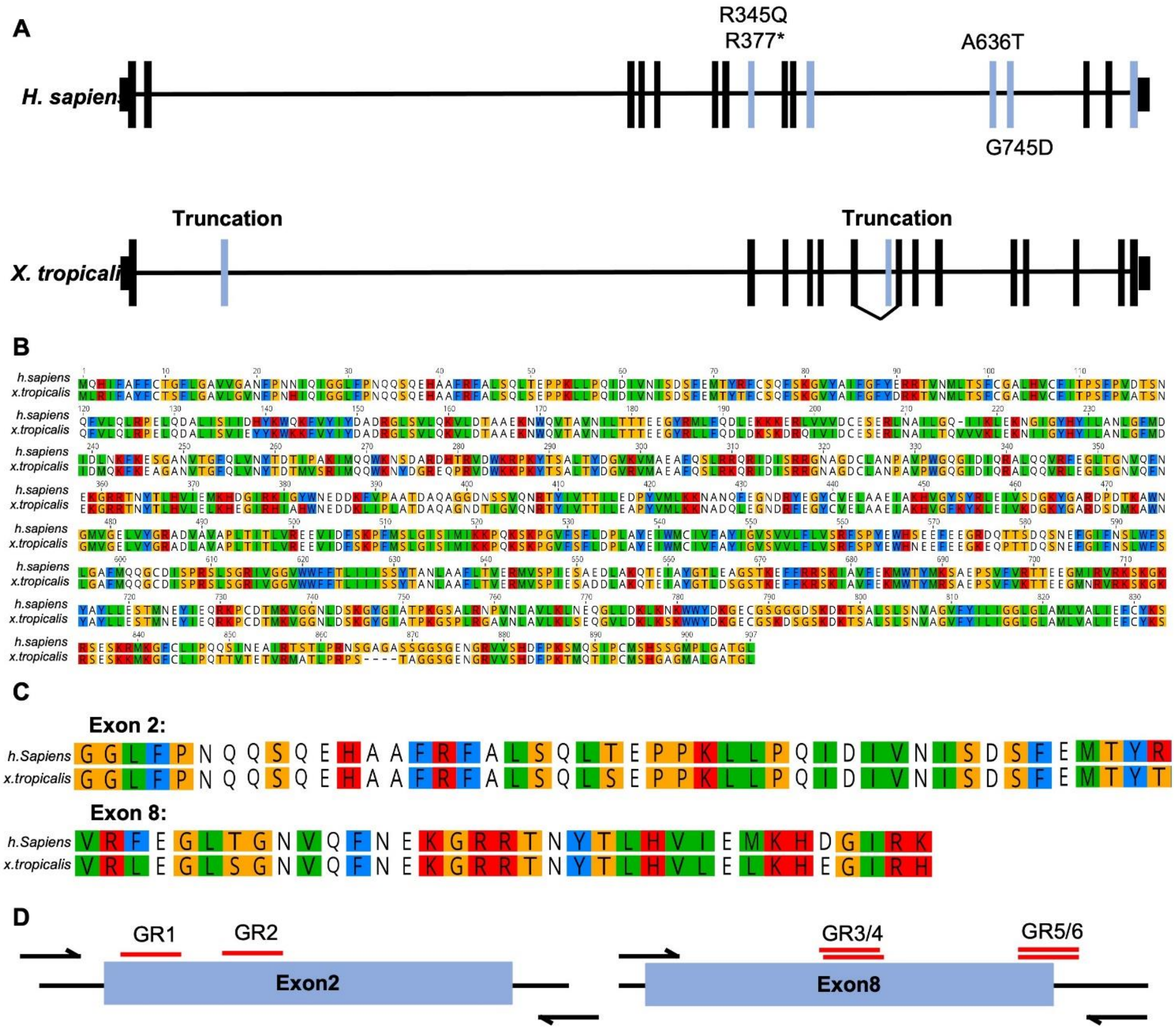

Figure S1. **Glutamate Ionotropic Receptor AMPA Type Subunit 1: gene structure, protein conservation and *X. tropicalis* target sites for disruption by CRISPR/Cas9.**

Xenopus tropicalis gria1, like *Homo sapiens gria1*, has 16 exons. The identified variants of unknown significance described causing GRIA1 Syndrome (bold) are shown spatially alongside the additional six variants listed on ClinVar. (A) The target exons for gene editing are highlighted in blue on the *X. tropicalis* model. (B-C) The *gria1* amino-acid sequence of *H. sapiens* and *X. tropicalis* aligned using Geneious Primer 2020 software (Biomatters, Ltd., Auckland, New Zealand) shows significant sequence conservation (>87%), particularly in the areas of interest in exons 2 and 8 (C). (D) The partial *X. tropicalis* exon schematics depict the position of the sgRNAs and genotyping primers for both the CRISPR screens.

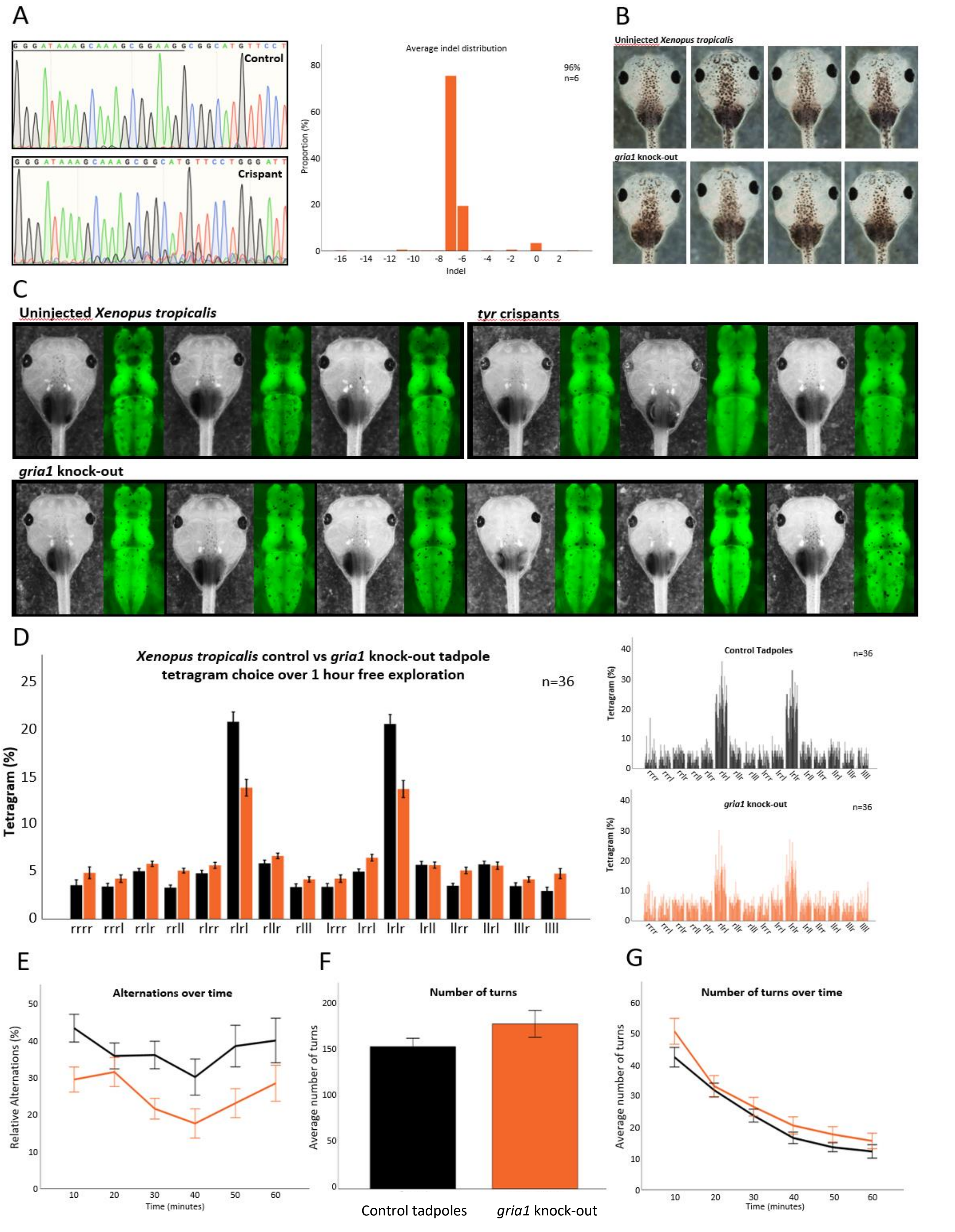

Figure S2. Characterization of tadpoles bearing CRISPR/Cas9 mediated insertion and deletion changes to *gria1* exon 2.

Fertilized *X. tropicalis* eggs were obtained in two mixed batches from three females and injected with sgRNA-GR2 targeting exon 2 of *gria1* and Cas9 protein. (A) Genomic amplicons were Sanger sequenced to confirm the presence of indels (*left*). The ratio of indels was assessed by Synthego ICE across six individual embryos, and the frequency is summarised as an average indel distribution in the mutant revealing a predominant 7 bp deletion. (B-C) Micrograph images of wild-type (*uninjected*), *tyrosinase* crispant (*tyr*), and *gria1* knock-out (*gria1*) tadpoles show head structural morphology under bright-field conditions (B) and whole brains in GFP fluorescence images. No morphological differences were observed in the forebrain, midbrain, and hindbrain regions of transgenic tadpoles [Xtr.Tg(tubb2b:GFP)Amaya, RRID: EXRC_3001]. *Gria1* knock-out tadpoles were generally indistinguishable from age-matched controls (*uninjected* and *tyr* crispant) both in their craniofacial appearance and brain length (forebrain to telencephalon: control Mean 2.24 mm, SD 0.3; knock-out Mean 2.14 mm, SD 0.29 [t_{70}=1.47; p=0.147], n = 36). (D) Relative frequency distribution plots of the free movement patterns of wild-type (*black bars*) and *gria1* knock-out (*orange bars*) tadpoles in the FMP Y-maze. Shown are summative (*left*) and individual (*right*) frequency distributions. For control animals, the alternating search patterns (LRLRL and RLRL) dominate, whereas for knock-out tadpoles, this dominance was significantly reduced. The data in the summative plot represents the mean ± SEM of all animals. (E) The number of alternations observed during the test period for wild-type (*black*) and *gria1* knock-out (*orange*) are shown in 10-minute time bins. *Gria1* knock-out tadpoles were observed to perform significantly fewer alternations than stage-matched control animals (ANCOVA: F(1, 69) = 18.9, p<0.001, n=36) throughout the trial. Data points represent mean ± SEM for all animals. (F & G) Overall, there was no significant difference in the number of turns performed by the control and *gria1* knock-out tadpoles (F: $t_{58.934}$ = -1.438, p=0.155), and all tadpoles were observed to perform fewer turns as the length of the trial increased (G: mean ± SEM).

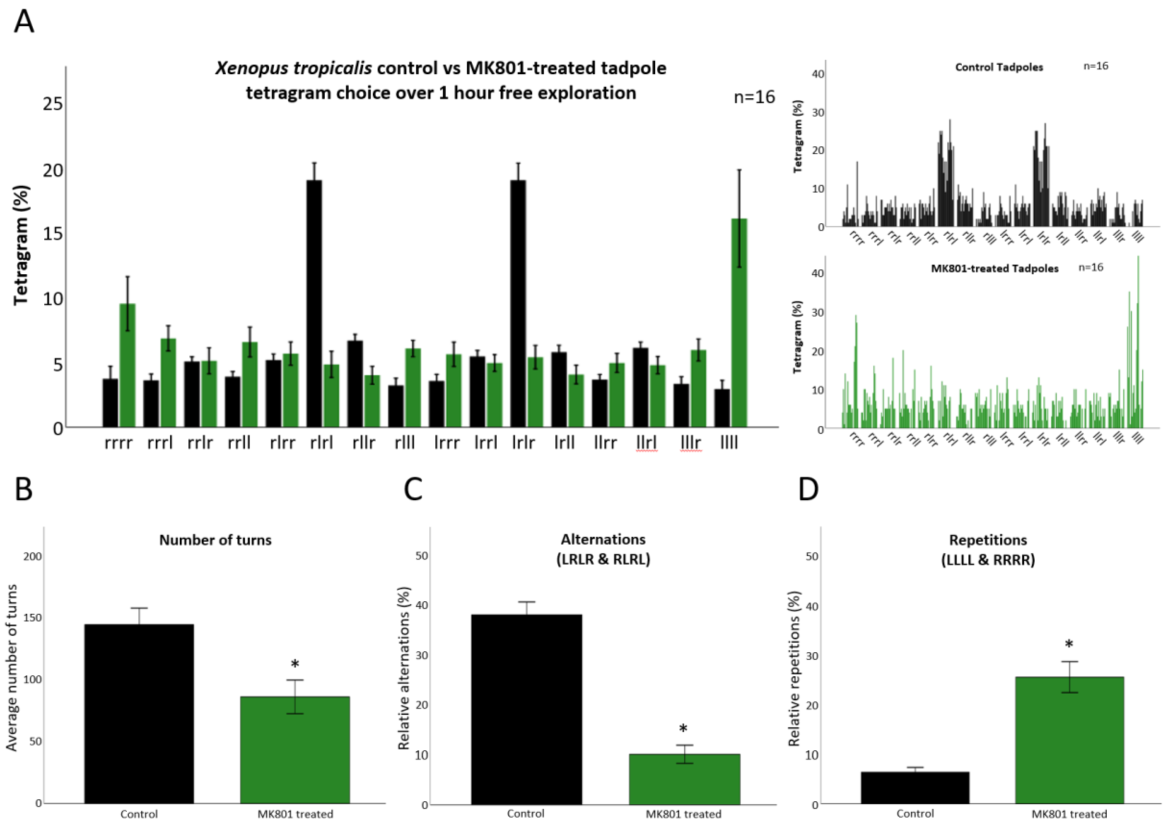

Figure S3. Implementation and pharmacological validation of the FMP Y-maze model for *Xenopus tropicalis* tadpoles.

Global tadpole search strategies in the FMP Y-maze were quantified as described in *Materials and methods*. The memory disrupting glutamate antagonist MK-801 was used to test whether search strategy patterns measure working memory in *Xenopus*. Wild-type tadpoles at stage NF50 were either treated with 0.75mg/L MK-801 (*green bars*) or remained untreated (*black bars*) in the two hours preceding behavioral analysis in the FMP Y-maze trial. (A) Relative frequency distribution plots of the free movement patterns of wild-type (*black bars*) and *gria1* knock-out (*green bars*) tadpoles in the FMP Y-maze. Shown are summative (*left*) and individual (*right*) frequency distributions. For control animals, the alternating search patterns (LRLRL and RLRL) dominate, whereas for MK-801 treated tadpoles, repetitive patterns (RRRR and LLLL) are dominant. The data in the summative plot represent the mean ± SEM of all animals. (B) The total number of turns during the trial for wild-type (*black bars*) and MK-801 treated (*green bars*) tadpoles. MK-801 treated tadpoles showed a significant decrease in the total number of turns performed across the trial ($t_{33.991}$ = 3.063, p=0.004). The data represent the mean ± SEM of all animals. (C - D) Direct comparison of the frequency of alternating search (LRLR and RLRL) and repetition (LLLL - RRRR) patterns between control and MK-801 treated tadpoles. Control tadpoles show a dominant search strategy of alternations (C: ANCOVA: F(1, 33) = 28.8, p<0.001; n=18), while MK-801 treated tadpoles display a dominant search strategy based on repetition (D: ANCOVA: F(1, 33) = 27.7, p<0.001; n=18). All data represent the mean ± SEM.

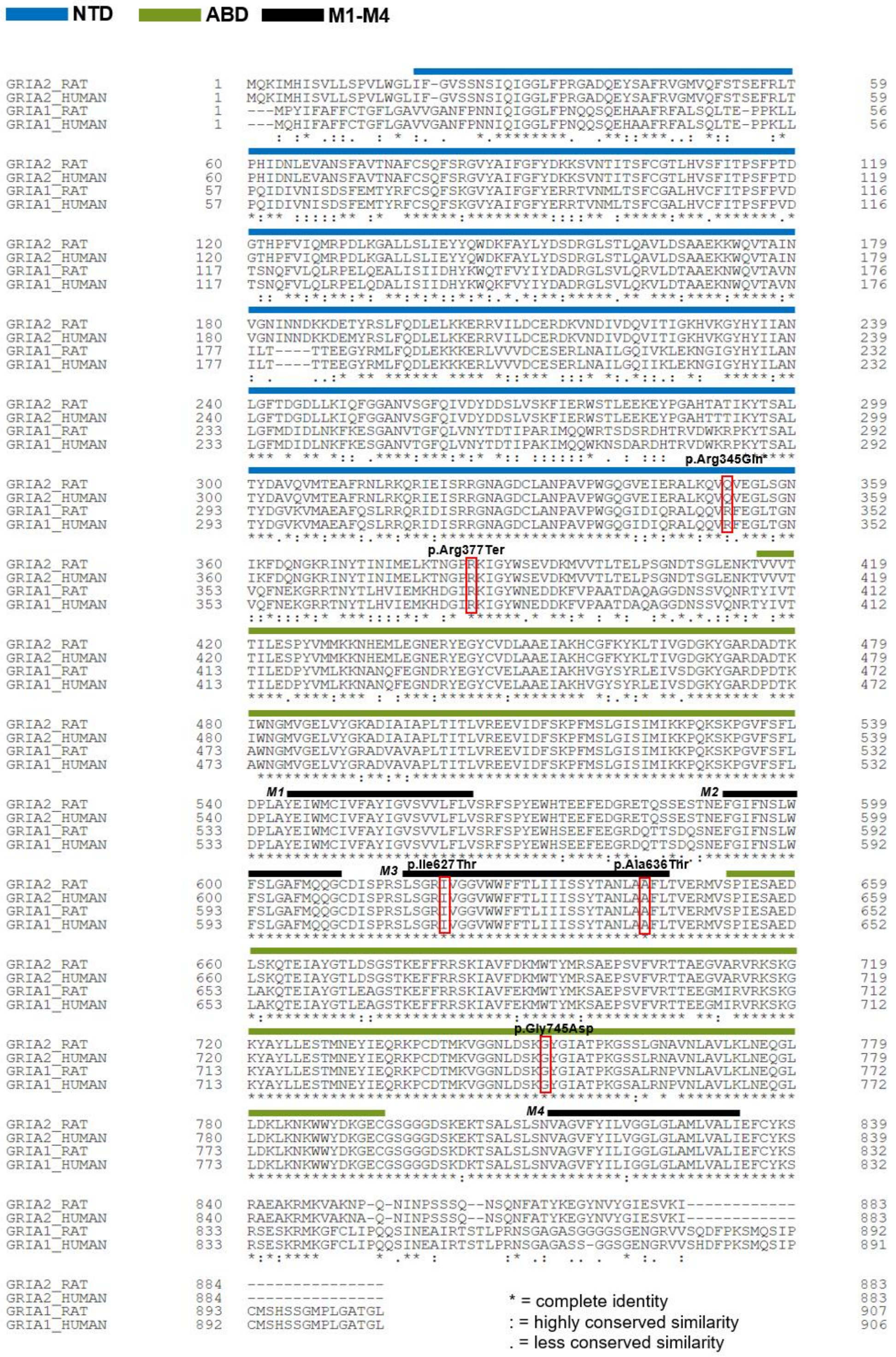

Figure S4. **Amino acid sequence alignment of human and rat GluA1 and GluA2 used for homology modeling of the human GluA1 receptor**.

The location of the NTD segment (*blue*), the ABD segments (*green*), and the transmembrane regions M1 to M4 (*black*) are depicted on top of the alignment. The residues affected by *GRIA1* variants are highlighted with red boxes.

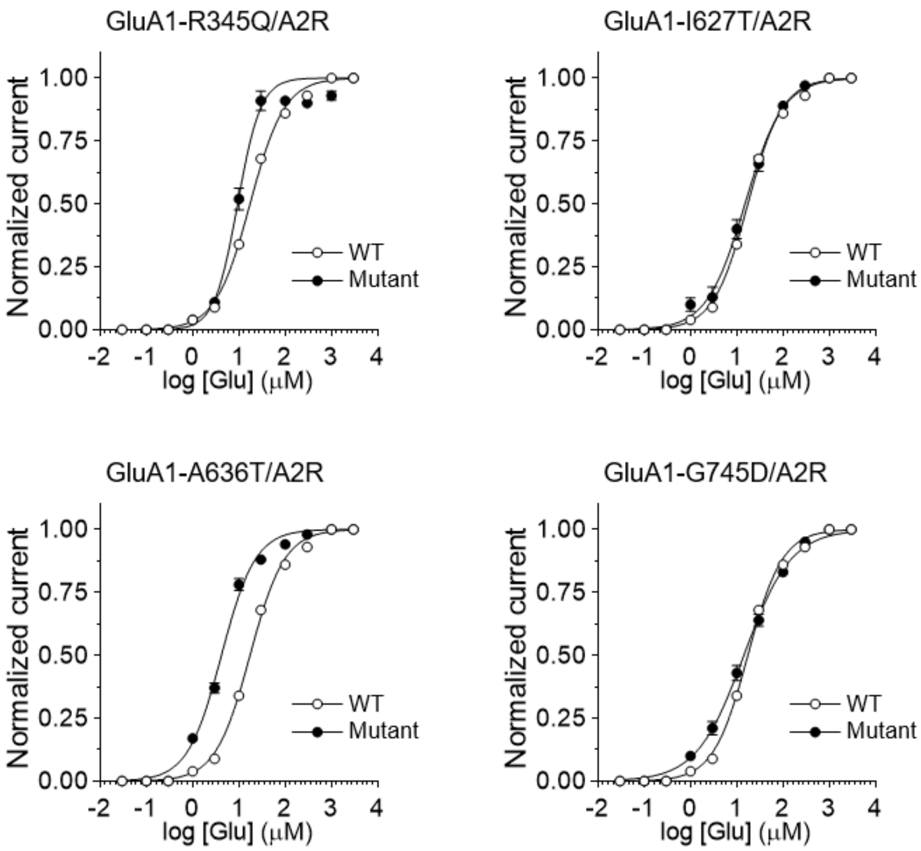

Figure S5. Glutamate concentration-response relationship for GluA1 mutants expressed as heteromeric GluA1/A2.

Composite concentration-response curves for glutamate at WT (○) and mutant (●) GluA1/A2 receptors were constructed using TEVC (*Materials and Methods*). Data represent the mean + SEM from 10-31 independent experiments.

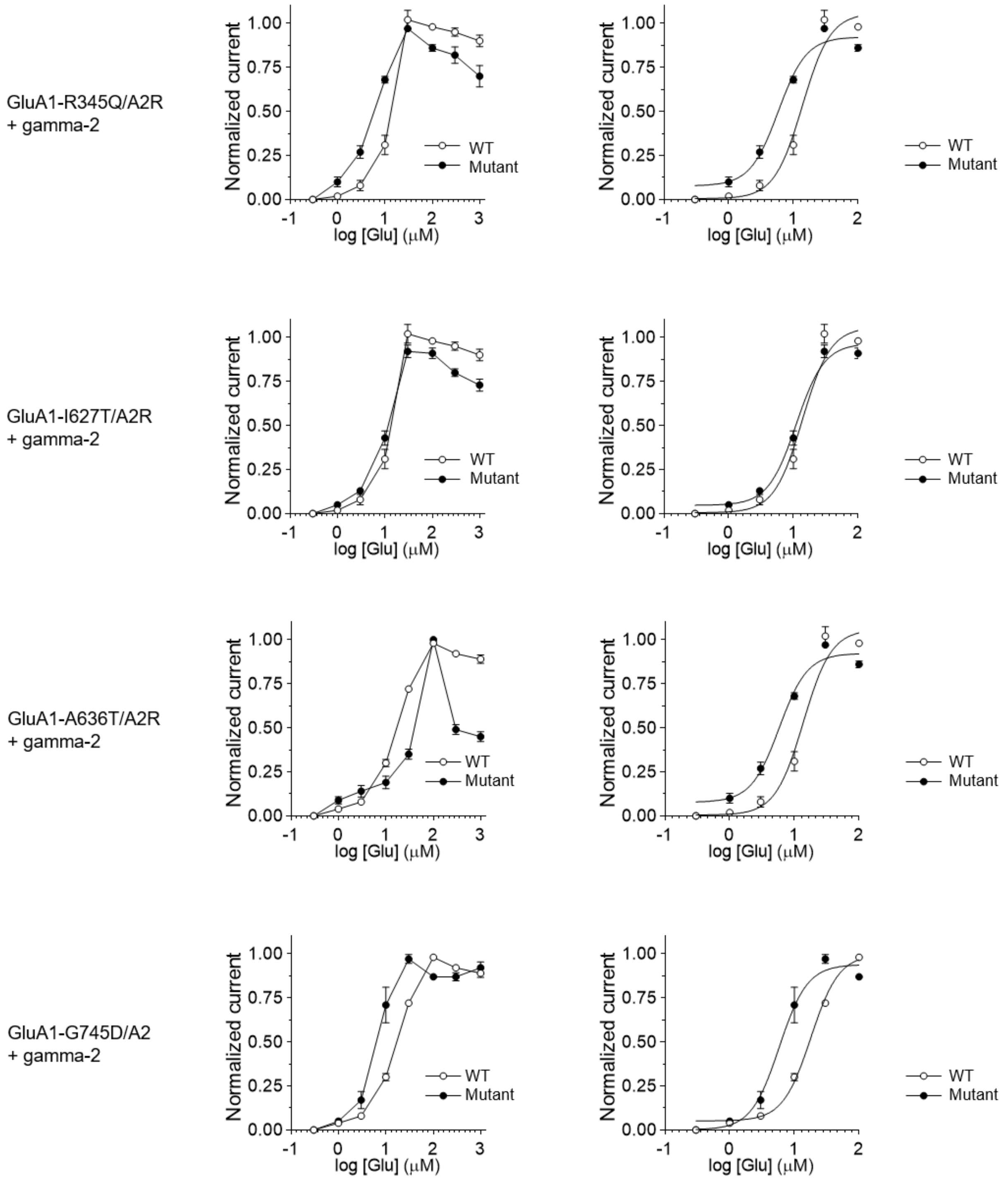

Figure S6. Glutamate concentration-response relationship for GluA1 mutants expressed as heteromeric GluA1/A2 in the presence of the TARP-class auxiliary subunit gamma-2.
Composite concentration-response curves for glutamate at WT (○) and mutant (●) GluA1/A2 receptors expressed with gamma-2 were constructed using TEVC (*Materials and Methods*). Data represent the mean + SEM from 10-31 independent experiments.

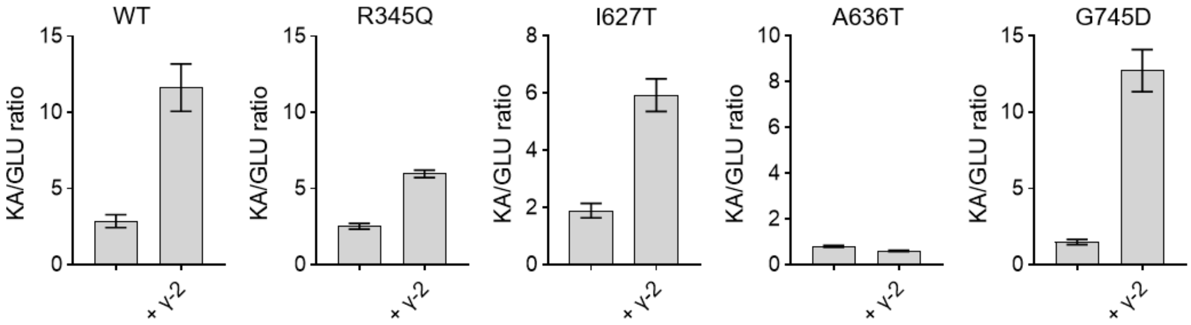

Figure S7. Control for the formation of gamma-2 complex for heteromeric GluA1/A2R receptors. The KA/Glu response ratio is increased upon co-expression with gamma-2 for all mutants and WT except for the A636T mutant. Data represent the mean + SEM from 10-31 independent experiments.

Gene	Target region	Primer sequence (gDNA)	#	CRISPR scan Score	Target sequence	Blast (v.10 *X. tropicalis*)	InDelphi Frameshift score
gria1	Exon 2: Gene knock-out	ACGACAGGAAAATAGCTGca AAGTGACCCCATTGCCTACA 435bp	GR1	75	GGTCCTGGGATTGCTGGTTT	16bp Chr8: pgk1 (non-coding), 16bp Chr4: non-coding	80.9% 64% +1, 17% +2
			GR2	61	GGATAAAGCAAAGCGGAAGG	17bp Chr1: non-coding, 16bp Chr1: LOC101730958 (non-coding), 16bp Chr7: non-coding	86.7% 17% +1, 70% +2
	Exon 8: p.Arg377* (homozygous)	ACTTCAAAGGTTCGGCTGGA CCTTTACCAGCTTAGTTGCCC 502bp	GR3	23	GGCGACCTTTCTCATTAAAC	17bp Chr2: non-coding, 17bp Chr1: ctxn1 (non-coding), 16bp Chr1: non-coding, 16bp Chr9: micall2 (non-coding), 16bp Chr8: non-coding, 16bp Chr5: non-coding, 16bp Chr3: non-coding	63.5% 34% +1, 29% +2
			GR4	33	GGTTTAATGAGAAAGGTCGT	17bp Chr3: non-coding, 17bp Chr1: ctxn1 (non-coding), 16bp Chr1: non-coding, 16bp Chr8: non-coding, 16bp Chr5: non-coding	61.1% 31% +1, 30% +2
tyr	Exon 2: Gene knock-out	CCTGCCGCTGACATATGGA GATGCCTCAGAGAGACGGAT 514bp	TY1	56	GGGGGTTCTGCTCCGATCG		84.7% 39% +1, 45% +2
			TY2	71	GGCTGTTGTAGGCAATCGGG	16bp Chr1: *rad9b* (non-coding)	74% 39% +1, 35% +2

Table S1. Design considerations for selecting efficient CRISPR/Cas9 oligonucleotides for gene-editing *in vivo*.

This table presents the *gria1* sgRNA CRISPR design considerations, providing an overview of each target (gene, target region, variant of unknown significance and genomic region of interest) and its associated oligonucleotide specificities (reference, CRISPRscan score, sequence, off-target blast hits, Indelphi frameshift frequency). Oligonucleotides were designed using CRISPRscan, which blasts the target genome (v9.1 *X. tropicalis*) against the reference sequence (c.50bp) and suggests constructs with on-target, efficient mutagenic potential (indicated by a high CRISPRscan score). All oligonucleotides were then manually blasted against either v10 (*X. tropicalis*) or v9.1 (*X. laevis*) of the *Xenopus* genome (Xenbase, e value 10), hits in grey represent genome locations lacking a suitable PAM (NGG) sequence, while hits in black show any potential off-target locations. Potential off-target hits differing more than 2bp from the original oligonucleotide sequence were also considered not viable. Fifty bp on either side of the expected CRISPR/Cas9 cut site was used to predict the likelihood of a deleterious frameshift variant (Indelphi). The frameshift score represents the overall proportion of variants that will generate an out-of-frame change, and this is then further subdivided into the proportion predicted to cause a +1 or +2 frameshift. The selected oligonucleotides above were synthesised by Invitrogen with a 5' T7 promoter (taatacgactcactata) and 3' overlapping Universal CRISPR segment (gttttagagctagaa).

SUPPLEMENTARY METHODS

Protein modeling – We modeled the flip isoform of the human homomeric GluA1 (hGluA1) receptor in the closed *apo* conformation (*resting*), the glutamate-bound, open-channel conformation (*active*), and the glutamate-bound, closed-channel desensitized conformation (*desensitized*). As templates for human GluA1 in these conformations, the following structures of the homomeric GluA2 receptor were used: Homomeric GluA2 in complex with the competitive antagonist ZK (PDB ID: 3RGK)[6] for the resting conformation, homomeric GluA2 with the auxiliary subunit gamma-2 in complex with glutamate and cyclothiazide (PDB ID: 5WEO)[7] for the open conformation, and the structure of homomeric GluA2 with the auxiliary subunit GSG1L in complex with the agonist *L*-quisqualate (PDB ID: 5VHZ)[8] for the desensitized conformation. Alignment of the rat GluA2 template structures to the hGluA1 sequence was aided by sequence alignment of the human and rat iGluA1 and GluA2 sequences (Figure S4). Homology modeling was performed using modeling software MODELLER (Sali and Blundell, 1993). For each confirmation of hGluA1, a total of 100 models were created and the best models were chosen based on Discrete Optimized Protein Energy (DOPE) scores calculated in MODELLER.

SUPPLEMENTARY REFERENCES

1. Sobreira, N., Schiettecatte, F., Valle, D., and Hamosh, A. (2015). GeneMatcher: a matching tool for connecting investigators with an interest in the same gene. Hum Mutat 36, 928-930.
2. de Ligt, J., Willemsen, M.H., van Bon, B.W., Kleefstra, T., Yntema, H.G., Kroes, T., Vulto-van Silfhout, A.T., Koolen, D.A., de Vries, P., Gilissen, C., et al. (2012). Diagnostic exome sequencing in persons with severe intellectual disability. The New England journal of medicine 367, 1921-1929.
3. Geisheker, M.R., Heymann, G., Wang, T., Coe, B.P., Turner, T.N., Stessman, H.A.F., Hoekzema, K., Kvarnung, M., Shaw, M., Friend, K., et al. (2017). Hotspots of missense mutation identify neurodevelopmental disorder genes and functional domains. Nat Neurosci 20, 1043-1051.
4. Guo, H., Duyzend, M.H., Coe, B.P., Baker, C., Hoekzema, K., Gerdts, J., Turner, T.N., Zody, M.C., Beighley, J.S., Murali, S.C., et al. (2019). Genome sequencing identifies multiple deleterious variants in autism Individuals with more severe phenotypes. Genetics in medicine : official journal of the American College of Medical Genetics 21, 1611-1620.
5. Landrum, M.J., Lee, J.M., Benson, M., Brown, G.R., Chao, C., Chitipiralla, S., Gu, B., Hart, J., Hoffman, D., Jang, W., et al. (2018). ClinVar: improving access to variant interpretations and supporting evidence. Nucleic Acids Res 46, D1062-D1067.
6. Sobolevsky, A.I., Rosconi, M.P., and Gouaux, E. (2009). X-ray structure, symmetry and mechanism of an AMPA-subtype glutamate receptor. Nature 462, 745-756.
7. Twomey, E.C., Yelshanskaya, M.V., Grassucci, R.A., Frank, J., and Sobolevsky, A.I. (2017). Channel opening and gating mechanism in AMPA-subtype glutamate receptors. Nature 549, 60-65.
8. Twomey, E.C., Yelshanskaya, M.V., Grassucci, R.A., Frank, J., and Sobolevsky, A.I. (2017). Structural Bases of Desensitization in AMPA Receptor-Auxiliary Subunit Complexes. Neuron 94, 569-580 e565.
